# Between communism and capitalism: long-term inequality in Poland, 1892–2015

**DOI:** 10.1007/s10887-021-09190-1

**Published:** 2021-06-02

**Authors:** Paweł Bukowski, Filip Novokmet

**Affiliations:** 1 Centre for Economic Performance, London School of Economics and Political Science, Houghton Street, London, WC2A 2AE United Kingdom; 2 Institute of Economics, The Polish Academy of Sciences, Warsaw, Poland; 3 Institute for Macroeconomics and Econometrics, University of Bonn, Kaiserplatz 7-9, 53113 Bonn, Germany

**Keywords:** Income inequality, Transformation, Poland, D31, E01, J3, N34

## Abstract

We construct the first consistent series on the long-term distribution of income in Poland by combining tax, household survey and national accounts data. We document a U-shaped evolution of inequalities from the end of the nineteenth century until today: (1) inequality was high before WWII; (2) abruptly fell after the introduction of communism in 1947 and stagnated at low levels during the whole communist period; (3) experienced a sharp rise with the return to capitalism in 1989. We find that official survey-based measures strongly under-estimate the rise in inequality since 1989. Our results highlight the prominent role of capital income in driving the U-shaped evolution of top income shares. The unique inequality history of Poland speaks to the central role of institutions and policies in shaping inequality in the long run.

**Supplementary Information:**

The online version contains supplementary material available at 10.1007/s10887-021-09190-1.

## Introduction

Our understanding of the evolution of inequality and its determinants depends on the available empirical evidence. As we have obtained new evidence, charting inequality further back in time, the old paradigms have been challenged and new ones developed (Atkinson & Piketty, 2007, 2010; Kuznets, 1953; Piketty, 2001, 2014). Although numerous countries have been extensively studied, Poland has been missing from the picture. The episodes of state formation, war, socialism, transition into capitalism, and integration into the EU make Poland a particularly compelling case for studying the determinants and effects of income inequalities. More recently, Poland’s profound transformation from communism to a market economy happened in less than one generation, and the accompanying economic growth has been the fastest in Europe (Piątkowski, 2018), but little is known about which income groups have benefited from it. How do inequalities evolve in such fast-changing societies and what is the relative role of technological change versus transition policies and emerging institutions? The Polish economy has been also deeply transformed by the new wave of globalization. This is the only major European country which has recently experienced a substantial re/industrialization and a growing share in the World’s GDP (Baldwin, 2016). But we know little about the distributional effects of these processes.

This paper is a first comprehensive attempt to examine the long-run evolution of inequality in Poland in order to shed light on the determinants and implications of inequality. We combine tax, survey and national accounts data to provide consistent series on the long-term distribution of national income in Poland. First, we construct top income shares for the whole period from the end of the nineteenth century until today. Next, we combine household surveys and income tax data in order to provide more reliable estimates of the full income distribution in Poland from 1983 until 2015. More precisely, we use tax data on high-income taxpayers to correct the top of the survey distribution. We thus produce the first homogeneous series that offer a possibility to compare the level and evolution of income inequality in Poland both through time and across countries.^1^

**Fig. 1:**
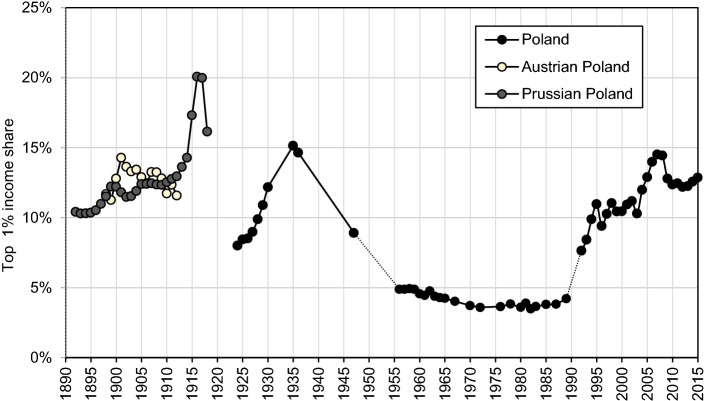
Top 1% income share in Poland, 1892–2015. *Source*: Authors’ computation based on income tax statistics. Distribution of fiscal income among tax units. Note: the Prussian Poland is the Province of Posen and West Prussia, the Austrian Poland is Galicia. For 1925–1937 Poland is the Second Polish Republic (with 1918–1939 borders), for 1945–1989 Poland is the Polish People’s Repulbic (with post-1945 borders), for 1992–2015 Poland is the Third Polish Republic.

Figure 1 summarizes our main results on the long-run income inequality in Poland.^2^ We interpret these results through the lenses of theories analysing the determinants of income inequality and the implications for economic development (Galor & Moav, 2004; Kuznets, 1955; Piketty, 2014). Table 1 provides a concise overview of Polish history since the nineteenth century and we follow the outlined periodization in our analysis throughout the paper.^3^

**Table 1:** The Polish history since the 18th Century

1772–1918	The Partitions of Poland between Austria (Galicia), Prussia (the Province of Posen and West Prussia), and Russia (the Congress Kingdom). As a result, Poland was removed from the map of Europe for 123 years. The occupying Empires imposed their own institutions
1914–1918	World War I – the occupying Empires fought on opposite sides, leading to massive destruction on Polish lands
1918–1939	Interwar Poland – the country was re-created and gained full independence. It drifted from a democratic parliamentary republic towards an authoritarian presidential republic. Predominantly an agricultural economy, with sluggish economic growth
1939–1945	World War II – Poland was occupied by Germany and Soviet Union and experienced the biggest relative war losses. Approximately 17% of the 1939 population were killed
1947–1989	Communism – Soviet Communist system with a centrally planned economy was introduced. Almost a complete elimination of private capital income, through nationalisation or expropriation. Industrialization and a strong economic growth during the first three decades, followed by a series of major economic and political crises in the 1980s
1989	Capitalism – a market-based economy with parliamentary democracy was re-established. In 2004 Poland joined the European Union. A period of very strong economic growth, re-industrialization and integration into the global economy

Top income shares in Poland followed a U-shaped evolution from 1892 until today. We find that top income share followed different trajectories in the Prussian and Austrian partitions, a steady rise in the former contrasting with the stagnation in the latter,^4^ which we link with the different political institutions and economic structures. The destruction of the First World War and a set of post-war shocks and redistributive polices led to a sharp reduction in top income shares. During the 1920s, however, top income shares recovered from this low-point and the Great Depression resulted in further concentration of income at the top. We document a high concentration of capital income at the top of the distribution throughout the first half of the twentieth century.

The introduction of communism led to a dramatic fall in top income shares. As documented now in many countries, the post-WWII downward trend in inequality was induced by the fall in capital income concentration. Communism signified comparatively greater shock to capital incomes relative to other countries, by literally eliminating private capital income with nationalisations and expropriations, while in addition it implied strong reduction of top labour incomes. During the remaining four decades of the communist rule, top income shares displayed notable stability at these—to some extent artificially—lower levels.^5^

**Fig. 2:**
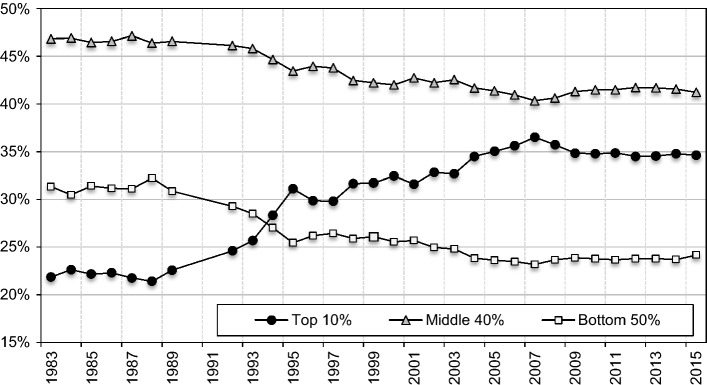
Income shares in Poland, 1983–2015. *Source*: Authors’ computation. Distribution of pre-tax national income (before taxes and transfers, except pensions and unemployment insurance) among equal-split adults

We analyse the transition from communism to a market economy by constructing the full income distribution (1983–2015) from combined tax and survey data. Figure 2 presents our series on the income distribution. We show that within one generation, Poland has moved from being one of the most egalitarian to one of the most unequal countries in Europe. Inequality experienced a substantial and steady rise after the fall of communism, which was driven by a sharp increase in the income shares of the top groups within the top decile. Over the whole 1989–2015 period, real income rose for nearly all income groups, but the top 1% captured almost twice as large a portion of the total income growth as the bottom 50% (24% versus 13%). The middle 40% and bottom 50% income shares experienced a similar evolution characterized by a stable fall from 1989 until the mid-2000s, and a stagnation afterwards. These results remain in contrast to the official survey-based measures, which substantially underestimate the rise of inequality since the end of communism, primarily by underestimating the top of the income distribution.^6^

The highest increase took place immediately at the onset of the transition in the early 1990s and was induced both by the rise of top labour and capital incomes. However, the strong rise in inequality in the 2000s, after Poland joined the European Union, was driven solely by the increase in top capital incomes, which are dominant sources of income for the top percentile group. Today, Polish top income shares are at the level of most unequal European countries, most notably Germany and the United Kingdom, but still substantially below those documented in Russia (Novokmet et al., 2018).

The new long-run inequality series present a critical input to investigate the determinants and implications of inequality. Overall, we point to the central role of country-specific institutions and policies in shaping the U-shaped evolution of inequality in Poland in the long run. The long-run evolution of top income shares presents suggestive evidence against the ‘natural’ decline of inequality along the development path. That is, the secular fall of inequality after WW2 in Poland was not an outcome of natural economic forces innate to the development process, nor its rise after 1989 can be explained by the new ‘technological revolution’. Although a closer look at certain episodes in Polish history underlines the importance of economic forces, in particular, technological change and globalisation, we argue that their distributional impact depends on a broader institutional setting (e.g., labour market institutions such as unions, minimum wage, corporate governance; provision of public education; government transfers and safety nets, or social norms towards inequality). Our series also shed new light on the role of inequality for growth in Poland and other post-communist countries. We highlight several ways in which institutional systems have mattered regarding the way inequality affected development through accumulation and efficiency channels.

The paper is organised as follows. Section 2 discusses the literature on the interplay between inequality and development through the lens of the Polish distributional history. Section 3 describes data sources and methodology. Section 4 presents the trends of the top income shares since the end of the nineteenth century until the end of communism. Section 5 presents the evolution and composition of the full income distribution since 1983 until 2015 and discusses the potential mechanisms at play. Section 6 compares the estimates for Poland with other countries. Finally, Sect. 7 concludes. Additional analysis is provided in the Appendix. The details of the data and estimation are discussed in the Online Appendix.

## Inequality and development: through the Polish lens

This paper lies at the intersection of different strands of literature looking at the interplay between inequality and growth. In what follows, we present those theoretical strands through the lens of Polish distributional history.

### Determinants of inequality

This paper shows the evolution of inequality in Poland in the long run and its determinants. Here our work directly builds on the top income literature. Kuznets (1953) first constructed top income shares for the US, which served as the empirical basis of the inverse-U curve, according to which inequality first increases and then declines as a country develops (Kuznets, 1955). A huge (‘Kuznetsian’) literature subsequently emerged investigating the distributional effects of economic development. One of its features is that the evolution of inequality along the development path is primarily driven by economic forces.^7^

Economists have generally discussed the distributional impact of growth through the distributional effects of factor accumulation and technical change (e.g., Bourguinon 2005). The instance of the ‘skill-biased’ technological change has figured prominently in debates discussing the recent changes in earning inequality. Analysed in the ‘demand and supply of skills’ framework, it can generate a Kuznets-type process: inequality initially rises as technological advances (also augmented by globalization) increase the demand for skilled workers, but then falls as the rest of the workforce acquire new skills and enter high-productive sectors (the ‘race between technology and education’; Galor & Tsiddon, 1997; Goldin & Katz, 2008; Tinbergen, 1974, etc.).^8^ The cyclical evolution of inequality is thus sometimes seen as inherent to the development process, when growth is driven by consecutive skill- or capital-biased technical changes.

Piketty (2001, 2006, 2014) has challenged the optimistic message inherent in the Kuznets’ process. He argues that the ‘great levelling’ of the twentieth century was a historically unique episode and that there is no spontaneous fall in inequality. Instead, he has emphasized the important role of policies and institutions in shaping inequality in the long run. For example, the recent rise of inequality in countries such as the US is largely a consequence of changes in policies and labour market institutions, such as lower tax progressivity, deregulation, erosion of unions or decline in minimum wage (Levy & Temin, 2007; Piketty & Saez, 2003, etc.).^9^

Overall, it has been recognized that to assess the complex effects of development on distribution, it is necessary to study the development process of individual countries. This allows us to assess the role of country-specific institutional contexts and policies in shaping inequality (e.g. Kanbur, 2000; Piketty, 2006). Furthermore, the comparative historical account provides a unique perspective from which to assess the relative importance of different factors in determining inequality. This paper is a step in this direction. We next discuss what the Polish experience tells us about the determinants of inequality in light of these theories.^10^

The unique Polish distributional history highlights the central role of institutions and public policies in shaping inequality in the long run. We find that the U-shape development of inequality in Poland was critically shaped by the changes in the institutional structures, as most clearly manifested in unparalleled changes in the labour market and capital ownership arrangements which have accompanied the rise and fall of communism (Sects. 4.3 and 5.2). This presents suggestive evidence against the ‘natural’ decline of inequality along the development path as envisaged by the Kuznets process. Moreover, a broad synchronisation of top income shares in communist and non-communist countries in the long run suggests that institutional and political factors played a decisive role both in reducing inequality in the first half of the twentieth century and in inducing an upward turn in inequality since the 1980s (Sect. 6; Atkinson, 2015; Atkinson & Piketty, 2007; Piketty, 2001).^11^ In this respect, the evolution of inequality in Poland may be seen as an extreme version of the inequality dynamic observed in Western countries.

This is not to say that economic forces do not matter as determinants of inequality. We indeed show that the models emphasizing the importance of market forces, in particular of technological change and capital accumulation, are useful for understanding Polish inequalities in particular moments of the country’s history. However, we argue that their distributional impact depends on a broader country-specific institutional and social context. This interaction is visible during two periods of Polish distributional history—industrialization in pre-WW2 Poland and the transformation to capitalism after 1989.

Using regional data for interwar Poland, we find patterns broadly consistent with the predictions of the upswing of the Kuznets curve (Appendix 1 and Sect. 4.2), which suggests that productivity advances shifted economic activity to cities and led to an increase in inequality. Yet, different institutional endowments inherited from the Partitions period (1772–1918) mattered for the distributional impact of the ‘first’ industrialization. For example, the legacy of ‘agrarian capitalism’ amplified inequalities in the case of former Prussian counties (Appendix 1 and Sect. 4.1). More generally, this suggests that neglecting country-specific institutions and policies presents the main obstacle in accounting for deviations from the Kuznets curve in empirical research (Kanbur, 2000; Williamson, 1991). The distributional effects of ‘classical’ industrialization cannot thus be generalized without considering a wider context (such as the general level of nutrition and hygiene, public infrastructure and education, the balance of political power, social legislation and safety nets, etc.) (Sects. 4.2. and 4.3). As a revealing case in point, the distributional impact of ‘socialist industrialization’ in Poland critically depended on its distinct institutional and socio-political features, in particular, the unique pattern of capital ownership or social policies aimed at elimination of mass poverty and improvements in healthcare (Sects. 2.2 and 4.3).

We devote special attention to the analysis of inequality determinants after the fall of communism, in particular, the role of institutional reforms, technological change and globalisation. Following the canonical ‘demand and supply of skills’ framework, we highlight the role of the demand for higher-educated workers in driving an increase of earnings inequality after 1989 (Appendix 2). We also show that a rise of top income shares, driven largely by top business incomes, is closely linked with the evolution of the capital share and the export share of GDP (Sect. 5.2). But here as well, we show that the country-specific institutional setting critically mediated the effect of market forces. This is visible in divergent inequality trajectories in Poland and Russia during the transition to a market economy, which we link with the different transition strategies (Keane & Prasad, 2002; Novokmet et al., 2018). In particular, we provide suggestive evidence for the role of minimum wage and privatization in driving the difference in the rise of inequality between the two countries (see Sect. 5.2).

### The effects of inequality on growth

The theoretical literature has broadly examined the general effects of inequality on economic growth through: i) factor accumulation (investment in physical and human capital), and ii) efficiency in using factors and technology (Weil, 2013, ch.13).^12^

*Factor accumulation channel*. The ‘classical’ approach posits that inequality is beneficial for economic growth as it shifts resources to rich individuals, who have higher marginal propensity to save, thereby leading to higher aggregate savings and higher physical capital accumulation, and hence growth (Bourguinon, 1981; Kaldor, 1957; Keynes, 1920; Lewis, 1954, etc.). The ‘modern’ approach, first advanced by Galor and Zeira (1993), argues instead that in the presence of credit market imperfections, inequality may adversely affect human capital formation and economic development. Galor and Moav (2004) have reconciled these views in the unified theory of the dynamic effects of inequality on development*.* In the unified framework, the way inequality affects growth depends on the relative return to human and physical capital. In the early stages of the development, when the physical capital is crucial for growth, the beneficial impact of inequality through the saving channel dominates the negative impact of inequality on human capital formation. And vice versa in the modern period when human capital is the key for development. Our series offers a possibility to assess this framework for the development process of the ex-communist countries.

According to Galor and Moav (2004), physical capital accumulation was key during the take-off from Malthusian stagnation (i.e. the Industrial Revolution; Galor, 2005; Galor & Weil, 2000). The partitioned Poland underwent a phase of classical industrialization from the mid-nineteenth century until WW1. This may be primarily seen in the rise of industry in the Congress Kingdom (e.g. textiles in Łódź, manufacturing in Warsaw; Wandycz, 1974), but also in the ‘agrarian capitalism’ in Prussian Poland. The presence of the ‘classical channel’, according to which inequality ensured necessary capital accumulation, may only be indirectly inferred from higher concentration of capital income at the top (see Sect. 4.1). Correspondingly, a shift of the political power from capital to labour after the unification of Poland in 1918 may have possibly discouraged accumulation and precipitated the sluggish interwar growth.^13^ Overall, despite occurrences of industrial development, Poland was still in the first half of the 20th c. predominantly an agricultural economy. Levels of industrialization and urbanization were among the lowest in Europe (Buyst & Franaszek, 2010, T. 9.1; Malanima, 2010) and the slow economic growth implied no convergence with Western Europe (see Figs. 10 and 13).

After WW2 Poland experienced three decades of strong economic growth. The introduction of communism brought a massive accumulation of physical capital (Feiwel 1981). However, due to comprehensive nationalization of the means of production, the positive effects of inequality on aggregate saving operated through a more pronounced functional, rather than personal, inequality.^14^ Higher profits, and hence higher investment, were in part ensured at the expense of the lower wage fund (and sacrifices in consumption). The beneficial effects of (functional) inequality on growth were operative because the aggregate productivity of physical capital is generally independent of its distribution (Galor, 2011; Galor & Moav, 2004). The rapid industrialization of Poland accentuated the demand for human capital due to capital-skills complementarity (Galor & Moav, 2004)*.* In this respect, communists fostered investments in human capital, such as school construction projects, development of specialized vocational schooling, or promoted female labour participation.^15^ In addition, communist reforms contributed to sweeping away feudal growth-inhibiting institutions—pervasive in Poland until well up WW2—and inducing structural change (see Sect. 4.3; Galor & Moav, 2006; Milanović, 2019; Piątkowski, 2018).

The fall of communism and the transition to a market economy amplified the complexity of the inequality-growth nexus, entailing a change in the relative returns and the way inequality affects growth. Lower inequality at the outset of the transition, with binding credit constraints, was conducive to the large expansion of tertiary education documented, which has been critical for the country to successfully adapt to the global environment and to approach the technological frontier (Galor & Weil, 2000; Goldin & Katz, 2008; Schultz, 1975).

On the other hand, the transition to a market economy entailed a renewed role of physical capital accumulation as the engine of growth. Actually, capital deepening has been one of the key contributors of labour productivity growth in Poland since the mid-1990s (see Gradzewicz et al., 2014; Rincon-Aznar et al., 2014). Indeed, Baldwin (2016) calls it re-industrialization and puts Poland as part of the global ‘Industrializing Six’. Thus, more pronounced top-end inequality in Poland, as evidenced in this paper, might have been growth-enhancing if top income groups had higher saving rates. However, opening to international capital flows might have reduced the beneficial effects of (interpersonal) inequality on growth through the saving channel.^16^

*Efficiency channel*. Inequality may stimulate growth due to investment indivisibilities, that is, when set-up costs of new businesses are high and capital markets are highly imperfect (e.g., Aghion et al., 1999; Banerjee & Newman, 1993). Indeed, this reasoning has been often used to point to the beneficial effects of inequality for the transition countries. Privatization of state-owned enterprises was intended to channel resources to the highest-value users to improve efficiency and strengthen profit incentives (Roland, 2000). Relatedly, it has been often argued that low inequality during communism adversely affected work incentives and effort. In this respect, a decentralization of the wage setting process after communism and the resulting earning decompression—especially in the upper part of the distribution (see Sect. 4.3 and Fig. 5)—may have improved incentives and stimulated growth.^17^

However, an important takeaway from the ‘modern’ approach is that there is a need for sustained redistributive policies (especially in the presence of credit market imperfections in investment in education; Aghion et al., 1999). Indeed, recent empirical research has found that (non-extreme) redistribution is not harmful for growth (e.g., Ostry et al., 2014), particularly if it promotes productive policies, such as investment in education, healthcare or infrastructure (Lindert, 2004). In contrast, higher inequality may hurt growth through the political channel exactly by impeding implementation of these kind of policies (e.g. through lobbying or corruption) and generally through socio-political instability (e.g., Alesina & Perotti, 1996; Easterly, 2001; Galor et al., 2009). Indications of the operating political channel may be inferred from the recent sharp political polarisation of Polish society (Lindner et al., 2020).

## Data and methodology

We combine tax, survey and national accounts data to construct our measures of long-run income distribution. First, we construct new consistent series on the entire income distribution for the 1983–2015 period. More precisely, we correct the top of the household income survey distribution with administrative data on high-income taxpayers. The methodology follows the Distributional National Accounts (DINA) guidelines (Alvaredo et al., 2016). For the earlier period, we focus on top income shares due to the absence of viable household survey data. We combine income tax and national accounts data to construct top income shares for the whole period from the end of the nineteenth century until today. Online Appendixes OA.1–OA.3 discuss the data and methodology in detail.

### Data sources

*Income Tax Statistics in Poland.* We first present data sources on income tax statistics, which present the central block for the construction of top income shares and the distributional national accounts. The first modern income tax in the Polish lands was established by the Prussian (1891) and Austrian Empires (1898) during the Partitions of Poland. Both Prussian and Austrian income tax statistics provide tabulations of income taxpayers in a regional breakdown, which has allowed us to construct top income shares for provinces with significant Polish population (the Province of Posen and West Prussia in Prussia; Galicia in Imperial Austria). There is no tax data for the Russian Partition (the Congress Kingdom), as comprehensive income tax was never introduced in Imperial Russia.

In 1924, the newly independent Poland introduced a unified progressive income tax for its whole territory. Detailed interwar income tax statistics were published separately for unearned income (*fundowany*) and earned income (*niefundowany*), organised by a large number of income brackets containing the number of taxpayers in each bracket and their corresponding tax obligation.

The communist government was established in 1947, so the interwar income tax system was still in use for several years after WWII, and the income tax tabulations are available for 1945–7. However, with the waves of nationalisation and the elimination of the private sector in the late 1940s, the personal income tax de-facto disappeared along with tax statistics. Instead, we combine different data sources to construct top income shares during the socialist period. As our starting point, we look at the detailed earnings statistics, published annually since 1956. The statistics covered all workers in the socialised sector, which accounted for the greatest part of the labour force (Atkinson, 2008; Atkinson & Micklewright, 1992). Published tabulations range workers in a large number of brackets according to the size of their gross earnings, providing, as a result, a detailed insight into both the upper and lower tail of the earnings distribution.^18^ Next, we assume that top earnings in the earnings statistics are representative of the right-end tail of the income distribution^19^ (more precisely, we focus on brackets covering 5% of individuals with highest earnings), while the remaining adult population is bulked into the bottom bracket (the bottom 95%). We attribute the remaining income from self-employment and social transfers to the bottom bracket.

For the post-communist era, income tax data come from the annual reports on the settlement of the personal income tax (PIT). The tax administration has published annual income tax tabulations since 1992. Tabulations are organised by income ranges that correspond to tax brackets as defined by the progressive tax schedule, with each bracket containing the number of taxpayers, their total income, deductions and the corresponding tax obligation. Due to the limited progressive structure of Polish PIT, the number of brackets in published statistics has been relatively small (generally equal to three for the post-communist period), with more than 90% of taxpayers located in the bottom bracket. As a result, although a great majority of the population is subject to PIT, the available income tax tabulations cannot be used to recover the entire income distribution. Instead, we use information on high-income taxpayers in combination with the household survey data.

*Household income surveys*. A Household Budget Survey (HBS) has been regularly conducted in Poland since 1957. Until 1972, it covered only employees in the socialized sector, excluding agriculture. Thereafter, it was conducted for four types of households: worker, mixed, farmer, and pensioner households (Atkinson & Micklewright, 1992, pp. 258–263). The survey underwent important changes after 1990, becoming fully representative of the population (Kordos et al 2002; Keane & Prasad, 2002; Milanović 1999).

For the 1980s, we use the Polish Household Budget Survey (HBS) data from Atkinson and Micklewright (1992, Tables PI1 and PI2). The authors provide tabulations of the individual distribution of household income per capita by combining the distribution of income for four types of households from the official HBS reports.^20^ The tabulations are organized by eight income groups, providing in each case the number of individuals and the mean income. For the 1992–2004 period, we use harmonised HBS microdata from the Luxembourg Income Study (LIS). Finally, for the period 2005–2015, we use the EU Statistics on Income and Living Conditions (EU-SILC). We harmonize the definition of income and the unit of analysis across the surveys.

*National accounts data*. We construct the total income and population controls based on the definition of income and the tax unit in the tax code. For the 1992–2015 period, we use National Accounts data published by the Central Statistical Office of Poland (*GUS*). For the interwar period we rely on historical series published by Kalecki and Landau (1934) and series constructed by the Maddison project (Bolt et al., 2018). The data for the period before World War I comes from the Prussian and Austrian censuses, yearbooks and various monographies.

### Methodology

There have been long-standing attempts to combine various data sources in order to produce consistent series on the distribution of national income over time and across countries. One of the pioneering works was done on Poland by Jan Wiśniewski (1934), who combined numerous data sources, such as income tax data, occupational ‘social tables’, and census data, in order to estimate the income distribution in Poland in 1929. In recent years, the large body of empirical work on the income distribution has estimated long-run series of top income shares, by combining income tax tabulations with national account totals for the population and the income, and using Pareto interpolation techniques. The methodology was pioneered by Kuznets (1953) and recently advanced by Piketty (2001, 2014), Atkinson and Piketty (2007, 2010), among others. We produce top income shares in Poland for the whole period from 1892 until 2015. All details are provided in Online Appendixes OA.1–OA.3.

Although tax data has proven to be especially useful to chart the long-term dynamics at the upper end of the distribution, they are silent on the remaining part of the distribution. On the other hand, it is well documented that household income surveys underestimate top incomes, which can critically impact overall distributional measures and misread the general trends in the income distribution (Atkinson et al., 2011; Piketty & Saez, 2003). Consequently, there have been various attempts to combine administrative tax and survey data to obtain more reliable estimates of the income distribution (e.g. Burkhauser et al., 2016; Piketty et al., 2018). Major progress in this direction has been made by the WID.world project. We follow its Distributional National Accounts guidelines (Alvaredo et al., 2016; Piketty et al., 2018) in the construction of comprehensive measures of income distribution in Poland.

The general methodology we use to combine survey and fiscal data consists of two basic steps. In the first step we use the raw survey tabulations and generalized Pareto interpolation techniques (Blanchet et al., 2017) to estimate series on the distribution of survey income by generalized percentiles (g-percentiles). In the second step, we use tax data on high-income taxpayers to correct upwards the survey series and obtain corrected estimates of the distribution of fiscal income. We assume that survey data provide a reasonable description of the income distribution below the 85th percentile. On the other hand, we take that tax data is accurate above the percentile corresponding to the first available income threshold in tax tabulations (generally corresponding to the 95th percentile). We then apply the piecewise-linear correction factors *f(p)* between these percentiles.^21^

In our accompanying work in progress (Bukowski et al., 2021) we show that the above methodology produced series on income inequality remarkably similar to those based on the registry database of the universe of individual-level tax returns for the period 2004–2017 (see also Kośny, 2019 for Lower Silesia).

*Unit of observation*. The unit of observation is the individual aged 20 or more. Household income in the survey is equally split between adults who belong to the same household. We should bear in mind that when combining survey and tax data we make implicit assumptions that high-income individuals in tax data are either singles, or that spouses have reported equal income. Since the option of joint filing for couples is widely used (the tax unit in the tax code is individual), it is reasonable to assume that eligible high-income taxpayers file a joint declaration by equally splitting their income in order to reduce their tax obligation. According to the PIT reports, the majority of eligible taxpayers used the joint taxation.

A further issue is that the homogeneity over time can be impaired by changes in the tax unit in the tax code. While the data for the communist and post-communist period relates to individual units, the tax unit in Austria, Prussia, and in interwar Poland was the household, with the total income of household members ascribed to the head of the household.^22^ The total number of households is estimated as all adult above 20 years of age minus the number of married females. The corresponding data is taken from population censuses and annual figures from the statistics on the movement of the population, and linearly interpolated for missing years.

*Definition of Income*. We focus on the distribution of pre-tax income, which refers to the sum of all income flows going to labour and capital, plus pensions but before other taxes and transfers. This income concept corresponds to the concept of fiscal income reported in the income tax statistics (Alvaredo et al., 2016; Piketty et al., 2018).^23^

Taxable income in both Austria and Prussia, as well as in the interwar period was quite broad and allowed very few exemptions. The post-communist tax data include income from employment, pensions, income from non-agricultural business activity and special agricultural activity, income from self-employment, rental income and income from other sources.^24^ We account for the changes in the tax law, which modify the definition of income. There were no major reforms of the tax system during the interwar period. However, the post-communist tax law has been amended several times since 1992. Most importantly, since 2004 income from non-agricultural business activity (further referred to as business income) can be taxed separately using a newly introduced flat tax. See Online Appendix OA.1.7. for all details of how we combine statistics on business income taxed at linear rates and income taxed using the progressive scale.

As noted, when joining the survey and tax data, we produce the distribution of fiscal income. A distinction needs to be made between fiscal income and national income, which is defined as GDP minus consumption of fixed capital plus net foreign income (SNA 2008). A major difference is due to the fact that the national income includes in addition tax-exempt capital income, such as undistributed corporate profits or imputed homeowners’ rents. We do not impute these items due to data availability, but, in general, it has been found that the fiscal correction (using income tax data) accounts for the bulk of upward correction of raw survey inequality, and further adjustment for the distribution of tax-exempt capital income has been shown to be of limited importance (e.g. Novokmet et al., 2018). Most importantly, correcting income distribution by imputing corporate retained earnings is less important in Poland, because business income is predominantly taxed according to the pass-through concept and hence attributed to individuals (Alstadsæter et al., 2017; Kopczuk, 2012).^25^ Alstadsæter et al. (2017, T.1) show that Poland has by far the highest share of employment in pass-through entities in Europe. Finally, in order to allow an international comparison, we scale fiscal income distribution up to the national income totals by proportionally upgrading thresholds and average incomes for each percentile of the fiscal income distribution.

## Top income shares in Poland 1892–1989

This section presents the evolution of top income shares in Poland from 1892 until 1989. Over this period, Poland experienced dramatic economic and political transformations. Until the 1980s, the real income per adult in Poland was around half of the income in Western Europe (Fig. 13), but the gap widened during the last decade of the communist period.

### Partitioned Poland and World War I

The three Partitions displayed different levels of economic development as well as specific institutions and different social conditions.^26^ Only Prussia and Austria introduced a comprehensive income tax in the Polish lands. We construct top income shares for the Austrian and Prussian partitions. Unfortunately, we omit the Russian partition as there are no comprehensive tax sources available. However, in Online Appendix OA1.3, we discuss alternative estimates of inequality in the Congress Kingdom.

Figure 1 shows the top 1% income shares in Poland from 1892 until today (see Online Appendix Tables OA6-OA9). We start with the Prussian Partition. Following a moderate rise from the 1890s until 1914, the top percentile increased sharply by 7 percentage points after the outbreak of World War I. The overall rise in top income shares was mostly driven by very high-income shares (see Online Appendix Figures OA3 and OA4, Table OA6) and almost exclusively due to a rise of very high incomes in rural areas, while shares of urban incomes remained stable throughout the whole period under consideration (Fig. 15). We believe that an explanation for the documented rise of top income shares in the Prussian partition should be sought in the growth of capitalist agriculture and the development of related industries (such as distilleries, sugar refineries or machines for agriculture, etc.) (Dumke, 1991; Grant, 2005). Diffusion of advanced farming techniques, related to an adoption of capital-intensive industrial crops such as sugar beet, contributed to a spectacular improvement in productivity in the Prussian partition between 1882 and 1907, surpassing that in the rest of Germany (see Online Appendix Figure OA5, Grant, 2002). Productivity advances were dominantly captured by the top of the distribution because of relatively high land inequality—an outcome of the Prussian land reforms, which favoured larger estates at the cost of smallholdings (Eddie, 2013; Grant, 2005; Mieszczankowski, 1960). Large estates were the driving force behind the structural transformation of agriculture in East Elbia, in what has often come to be generalised as the ‘Prussian’ road to industrialisation (Lenin, 1908). However, the causation might have run the other way, from inequality to growth, notably by alleviating accumulation constraints in line with the “classical channel” (see Sect. 2).^27^

Top income shares in the Prussian partition soared during World War I (Fig. 1 and Online Appendix Figures OA3-OA4, Table OA6). The economic environment favoured the capital owners, especially due to the wartime demand for armaments and food. The Allied blockade was the root cause of the German food problem, as it caused a plunge in food imports (Hardach, 1977; Ritschl, 2005). Food shortages led to a surge in prices, bringing, in turn, extraordinary profits to agricultural producers, which were proportionally more concentrated in Prussian Poland.

The Austrian Partition (also known as Galicia) was economically the least developed of the three ‘partitioned’ Polish regions.^28^ Top income shares in Galicia showed no clear trend in the two decades preceding WW1 (Fig. 1). The top 1% income share increased by almost 3 percentage points in the short period from 1898 until 1901, when it peaked at 14.3%. Afterwards we observe a falling trend during the years preceding WW1. We believe that top incomes in Galicia were mainly an urban phenomenon, given the backwardness of its agriculture and the notoriously poor living standards of the rural population—overwhelmingly living at the bare subsistence level (‘*Galician poverty*’; Bujak, 1908; Szczepanowski, 1888). As we look below, the rural–urban income gap figured prominently during the interwar period. Among the interwar Polish counties, those located in Galicia were characterized by the highest correlation between urbanisation ratio and top 1% income shares (see Appendix 1). Further, modest industrialization in Galicia since the end of the nineteenth century had an important resource-export dimension (crude oil and salt mining; Frank, 2005), possibly entailing a relatively larger importance of non-tradable services in cities (e.g. commerce or personal services; Gollin et al., 2016).^29^

The positive relationship between inequality and growth may be postulated both for Austrian and Russian industrialization (Table 3 shows the positive role of urbanisation and industrialization for counties in the former Russian partition during the interwar period; see Appendix 1). Conceivably, inequality tendencies were further strengthened as the accelerating rate of capital accumulation was accompanied by the ‘surplus’ of unskilled labour amid a strong population increase during the Post-Malthusian phase (further reinforced after the serf emancipation in Austria in 1848 and especially in Russia in 1864).^30^

### Interwar Poland

The unification of Poland in 1918 is one of the pivotal events in Polish history. Poland was established on the world map after 123 years under foreign dominions. However, this century-long dream had to be realised in quite a tumultuous atmosphere of economic crisis, broken international trade, and recovery from the massive destruction and human losses of the Great War.

Our starting point in the interwar period (1924) coincides with the lowest documented point in top income shares during the existence of interwar Poland, with the share of the top percentile slightly above 8% (Fig. 1). There are several arguments in favour of the lower top shares in the first half of the 1920s. First, Poland was among the countries that suffered the greatest losses during the First World War, both in the number of human casualties as well as in the extent of physical destruction (Landau, 1968). The deleterious effects of shocks to capital income in the interwar period are now well documented as the main reason behind the secular fall in top incomes initiated after WWI (Piketty, 2001; 2003).^31^ Next, the risk of political radicalization and the communist upheaval pressured the new leadership of Poland into passing new social legislation, such as the eight-hour working day, trade unions or the right to strike (Derengowski, 1930; Sztrum de Sztrem, 1922; Wolf, 2007). Further, various anti-capital policies were introduced, including a sharp increase in tax progressivity and heavier taxation of capital than labour (including a one-time capital levy).^32^ Industrial capital tied up in export sectors especially suffered from the loss of the large and protected Russian market and the Polish-German trade war of 1925. The currency stabilization in 1923 further negatively affected the international competitiveness of Polish products, while another great shock was hyperinflation.^33^ Generally speaking, the post-war situation worldwide signified altogether a new page in the distributional history in comparison to the pre-WW1 political and social setting (e.g. Keynes, 1920; Piketty, 2014; Scheidel, 2017; Scheve & Stasavage, 2016). Thinking in terms of predictions of the ‘physical capital regime’ in the unified framework of Galor and Moav (2004), it is interesting to ponder to what extent the distributional conflict accompanied by a shift of the political power from capital to labour discouraged capital accumulation and thus precipitated the sluggish interwar growth (Fig. 13).^34^

The following six years, however, saw a continuous rise in the top percentile share and reached almost 11% in 1930. The economy eventually stabilised in 1926, and the country experienced three years of steady growth, halted only by the advent of the Great Depression in 1929. The economic recovery brought better prospects for top incomes, which experienced a strong increase in the late 1920s.^35^

However, when the tax data become available in 1935, the series on top income shares re-emerged at 15%, which corresponds to its secular peak in the time of peace. All top income groups saw rising shares in this period, suggesting a rising dispersion between the top and the rest of the distribution (e.g. P0-99, Online Appendix Table OA7). Accordingly, it is plausible that this development indicates a deteriorating position of Polish farmers relative to other social groups. Almost two-thirds of the population was made of small farmers and it was agriculture that was most adversely affected by the Great Depression, in the first place due to a strong fall in agricultural prices (Landau, 1963). In contrast, the fall in industrial prices was much less steep due to rapid cartelization, which safeguarded industrial profits at the top.^36^ The deflationary trend was, on the other hand, beneficial to high-salaried employees that were able to keep their jobs due to rigid salaries, making this group a relative winner behind this development (Kalecki & Landau, 1935; Landau, 1933, Fig. 16).

**Fig. 3:**
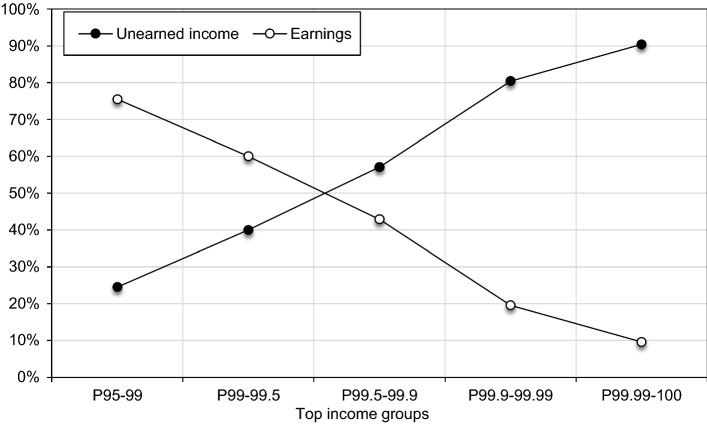
The composition of the top 5%, Poland in 1929. *Source*: Authors’ computation based on income tax statistics and Wiśniewski (1934)

The Great Depression led to a differential income fall for different top groups (see Fig. 16). The top 0.1% saw a proportionally stronger fall at the start of the crisis (1929–1931) than the lower groups in the top percentile. Yet, in 1935 (unfortunately, there is no data for three years after 1931) we find that top groups had managed to retain their relative standing. Plausibly, the rapid cartelization should be identified as the main tool allowing top incomes to steer through the crisis successfully, and the main beneficiaries should be searched for among the capital income recipients. Figure 3 shows the composition of top income groups in 1929 between earnings and other sources of income (defined in the tax statistics as ‘unearned’ income, roughly corresponding to the broad definition of capital income). Groups below the top percentile, such as the top 5–1%, were mainly composed of earnings. The importance of earnings, however, decreases with income rank. For the top 0.1% group, unearned income made up as much as 80% of the total income.

**Fig. 4:**
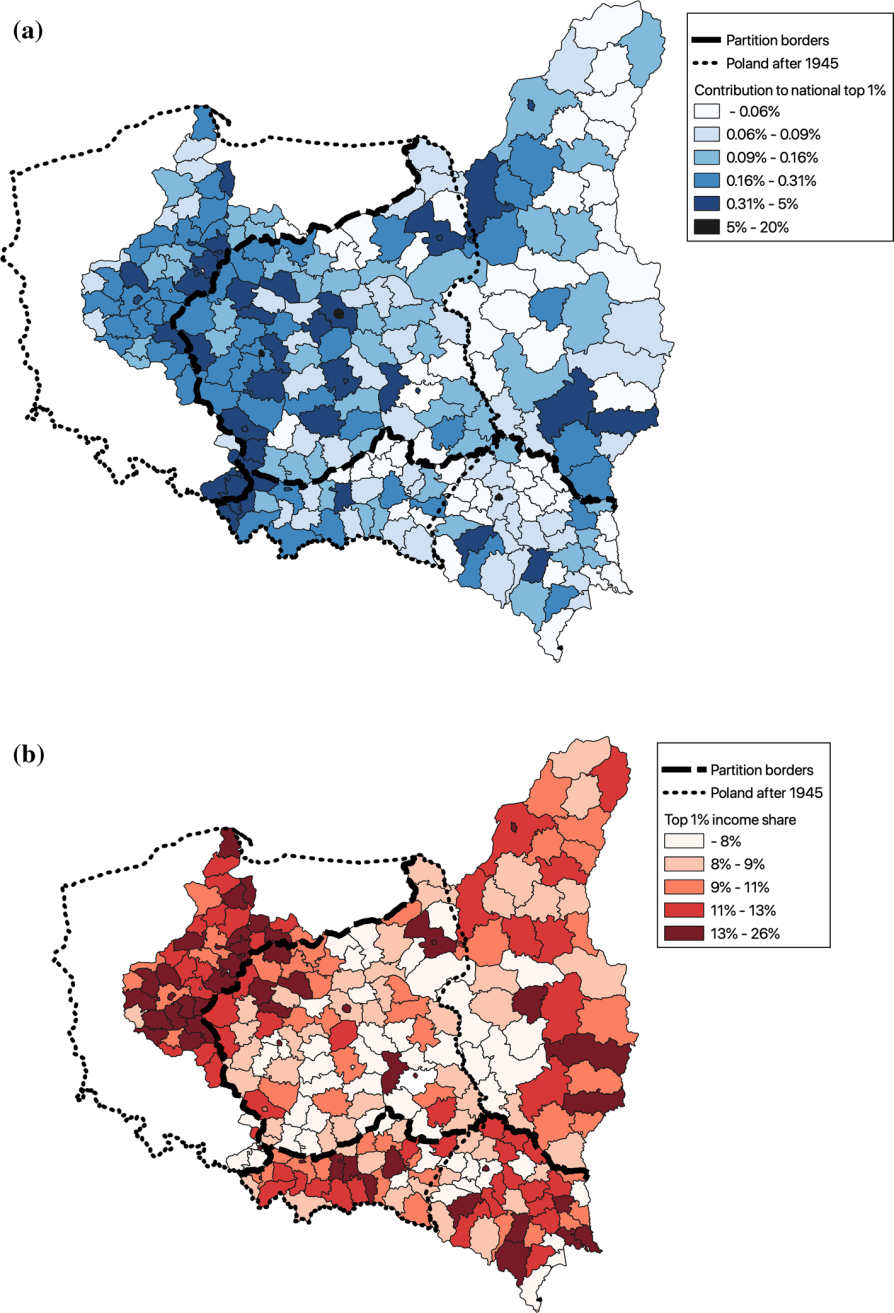
**a** County-level contribution to the aggregate top 1% income, 1927. **b** County-level top 1% income share, 1927. *Source*: Authors’ computation (see Online Appendix 1.5)

Further insights could be obtained by looking at the spatial distribution of county-level top income shares in Poland.^37^ Figure 4a presents the map of Polish counties in 1927 and the contribution of each county to the aggregate national top 1% income share. We find that the most significant contributor to the national top percentile is the size of the local economy (e.g. Warsaw, Łódź and Lviv contributed with around 40% of all top percentile constituents) (see more details in Appendix 1). Figure 4b displays county-level top 1% income shares (calculated using the county control population and total county income). The dashed line marks the former borders between the partitions. The geographic distribution of top income shares has a donut-shape, with high levels at the edges of interwar Poland and relatively low in the centre. The largest inequalities are in the former Prussian Partition (the west) and the eastern parts of the former Russian Partition (the east). The picture is less clear for the former Austrian Partition (the south and south-eastern parts), where there are no clusters of counties with high top income shares.^38^ Appendix 1 looks at the determinants of the county-level top 1% income share. We find that the county-level top 1% income share is positively associated with urbanization, employment share in industry and agriculture, and land inequality. However, we document heterogeneity in the importance of these factors across the former partitions of Poland (e.g. the importance of land inequality and the agricultural employment in the former Prussian partition is indicative of the ‘agrarian capitalism’). We also analyze a joint distribution of income and inequality for the rural and urban counties and find patterns broadly consistent with the predictions of the upward part of the Kuznets curve.

### Communist Poland

In theory, the distribution of income under a socialist state should be based on the rule “from each according to his ability, to each according to his labour” (Atkinson & Micklewright, 1992; Marx, 1875). The rule does not imply an inequality-free society, as long as production concerns are pressing. In practice, however, the introduction of communism in Poland and other socialist countries led to a dramatic reduction in income inequality. The communist reforms signified an unprecedented change in the institutional framework, as manifested in the abolition of the private ownership of the means of production and the comprehensive reorganization of labor market with the direct bureaucratic control of employment and wages. The communist accession to power was further accompanied by the comprehensive redistribution of income and wealth, as well as the promotion of egalitarian ideology (e.g. Bergson, 1944; Kornai, 1992).

The post-war development of top income shares clearly reveals a secular fall in inequality. Right after the Second World War, in 1946–7, we find top income shares at the level of 9%—a significant fall from the level of 14.6% in 1936 (Fig. 1). In order to understand this decline, one needs again to ascertain a development at the ‘bottom’ of the distribution. The post-WW2 years saw a relative improvement in the living conditions of the rural population in comparison to the devastating experience of the Great Depression. This came about in the first place through rising prices of agricultural products, large land redistribution, debt release and new social legislation, such as the increased availability of education in the countryside (Landau & Tomaszewski, 1985). These reforms also contributed to sweeping away feudal growth-inhibiting institutions, pervasive in Poland until well into WW2, and induced structural change (Milanović, 2019; Piątkowski, 2018; see also Galor & Moav, 2006; Galor et al., 2009).^39^

However, a decline in top concentration also ensued as communists strengthened their rule in the country, which led to an almost complete expropriation of capital income by the state.^40^ The most radical legislation in the direction of nationalisation was passed in 1947. In the succeeding years, during the so-called Battle for Trade (*Bitwa o handel*), even the majority of small shops and crafts were nationalised. The next decisive episode was the currency reform in 1950 that virtually confiscated all personal financial wealth.

As a result, labour income and the wage setting process become the main determinants of the interpersonal income distribution in a socialist society. Indeed, Atkinson and Micklewright (1992, p. 128) document that in socialist Poland “the changes [in income distribution] over time mirror those in the earnings distribution”.^41^ The wage setting process was largely centralized, with a limited role for incentive schemes. The wage structure across occupations/positions was used as a policy tool, for instance, to provide incentives for people to invest in particular skills, to stimulate the economy by widening earning differentials or to calm social dissatisfaction by narrowing them (Atkinson & Micklewright, 1992; Flakierski, 1986). In addition, bonuses were given to establishments whose performance was higher than expected. In practice, however, the wage levels were often more dependent on the political power of workers, managers and industry groups than on productivity (Brus, 1974).

**Fig. 5:**
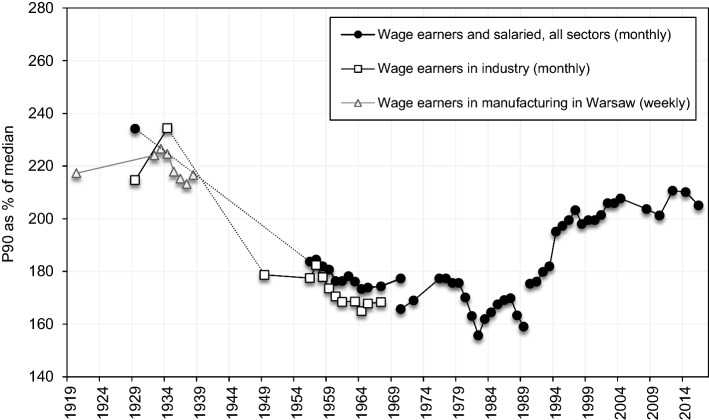
The upper part of the earnings distribution in Poland (90th percentile as proportion of median), 1919–2015. *Source*: Own construction: 1921–1949 and 2008–2016, and for manual workers 1956–1965; other years: Atkinson, 2008; Atkinson & Micklewright, 1992; Rutkowski, 2001; (see Online Appendix OA.3). Note: 1955–1989: monthly wage for employees in socialized sector (1956–1970: gross monthly wage; 1970–1988: net monthly wage), 1992–2016: gross monthly wage; the estimate for all workers in 1929 excludes employed in agriculture

Figure 1 depicts the evolution of top 1 percent income share during the communist period (see Online Appendix OA.1.6 for more details on the methodology). The inequalities slightly trended downward from 4.9% in 1956 to 3.4% in 1988, and the average level in this period is roughly half of the total top income shares in 1946 or 1992. Figure 5 presents the upper part of the earnings distribution, showing the evolution of the 90th percentile (expressed as a proportion to median) from the late 1920s until today. The evolution of wage ratios is more volatile than the top income share, yet the relative levels and trend are similar. It can be clearly seen that the upper tail inequality was substantially lower during communism than in the interwar period. The decline is further corroborated by the newly collected evidence on the wage distribution of manual workers. The figure shows a considerable upper tail inequality for manual industry workers in interwar Poland (the same is documented for manual workers in Warsaw) and a sharp decline after WWII.

Kalecki (1964) and Beskid (1964) show that earnings compression was primarily caused by a decline in premium between white-collar and manual workers. The fast industrialisation and urbanisation significantly improved living conditions of low and middle-income manual workers. But it should be remembered that there was a general reduction in skill differential, regardless of “collar denomination”.^42^ Figure 5 conveys that a part of the decline in earnings inequality is to be attributed to the wage compression among the manual workers.

The Communist government used institutional factors, such as unionisation or centrally determined wages and prices, to control real wages. During the early years of communism, exceptional power was given to the high-level managers, which increased within-firm wage dispersion (Brus, 1974). Popular dissatisfaction was growing and culminated in massive and violent protests of workers in Poznań in 1956. The Party reacted by expanding and giving semi-independence to unions, resulting in a decline of the wage decile ratio after 1956. Notably, the P95/P50 ratio falls more abruptly than the P90/P50 (Online Appendix Figure OA10), possibly owing to the decline in the power of high-level management. Giving too much power to unions, however, was also threatening the position of the Communist government. The “thaw” of 1956 ended in the early 1960s, when the Party turned towards a more centralised economy and scrapped the independence of workers’ bodies, leading to a period of modest growth in wage dispersion.

Low labour productivity was a plague of the socialist economy in the 1960s (Flakierski, 1986). The Party reacted in the early 1970s, with a set of limited marketization reforms, which would increase worker incentives by directly linking their wages with output. It is hard to assess whether this policy had any effect on productivity, as it was also a period of foreign loan-financed consumption growth. What can be seen, however, is that wage inequality entered a decade of continuous growth, driven by the growth of within-industry dispersion and to a lesser extent by a shift in industry composition (Flakierski, 1986).

A marked tension between efficiency and equity concerns, as a reoccurring feature of the socialist economy, became again pronounced in the early 1980s. The previous decade’s precedence given to efficiency was followed by the pronounced egalitarian demands of the “Solidarność” movement. A remarkable fall in the wage ratios should be in part attributed to government policies in response to the demands of the Gdańsk Agreement (Atkinson & Micklewright, 1992; Flakierski, 1986).^43^ However, this “honeymoon of egalitarianism”, in the words of Flakierski (1991, p. 99), was short-lived and the dispersion increased again after 1982.^44^

Next, we briefly discuss specific ways how inequality impacted growth through factor accumulation during socialist Poland (Sect. 2.2.). Poland experienced stronger economic growth in the three post-war decades (Fig. 13). It was higher functional inequality^45^ that sustained socialist industrialization. Higher profits, and hence higher investment, were in part ensured at the expense of the lower wage fund.^46^ At the same time, it may be surmised that substantial efforts to end mass (rural) poverty and improvements in healthcare ensured that hard work was performed during industrialization, and that malnutrition did not adversely affect the return to capital (Fogel, 1994; also Voigtländer & Voth, 2006). In addition, the rapid industrialization accentuated the demand for human capital due to capital-skills complementarity (Galor & Moav, 2004). Communists fostered investments in human capital in response, such as huge school construction projects, development of specialized vocational schooling, or promoted female labour participation.

However, there are qualifications to the above-mentioned features of communist development. It is now clear that a pronounced emphasis on physical capital accumulation, as practiced in communist countries (‘capital fundamentalism’), subsided over time (for alternative reasons see Allen, 2003; Easterly & Fischer, 1995; or Weitzman, 1970) and higher (forced) investment ceased to translate into higher growth (due to various ‘barriers and ceilings’ as posited by Kalecki).^47^ Further, it may be conjectured that centrally-determined wage control suppressed returns on education, which led to suboptimal individual investment in education.

Finally, we must consider the meaning of monetary inequality during communism, as summarized by our measures. Given the potential distributional implications of well-known non-monetary features of the socialist economy—such as shortages and queueing, widespread consumer price subsidies and price controls, extensive social benefits in kind, or various non-wage benefits of the communist elite, among others (Bergson, 1984; Atkinson & Micklewright, 1992, c.6)—it may be asked to what extent monetary inequality reflected the true inequality of living standards. Most importantly, the varying importance of non-monetary aspects of inequality could bias inequality comparisons over time and across countries.

Notwithstanding the importance of the distortions mentioned, the consensus reached by authorities has been that money incomes were the most important welfare dimension in the socialist economies (Atkinson & Micklewrigth, 1992; Bergson, 1944, 1984; Milanović, 1998; Rutkowski, 1996, etc.) and that monetary indicators may be taken as indicative of the broad development in living standards. We do recognize them as imperfect, but as pointed out by Bergson (1984, p. 1057) “the ideal is hardly realized anywhere”. Moreover, biases tended to balance on average, leaving the overall inequality largely unchanged (Milanović, 1998).^48^ Specifically, when it comes to (questioning) the reality of the increase of inequality in Poland during the transition, Rutkowski concludes that “biases seem to be of the second order and do not alter the main results” (1996, p. 91; Atkinson, 2008). In Online Appendix OA 1.6.2 we discuss in length the limitations and different interpretations of the official data on income distribution during the socialist period.

## The full distribution of income in the post-communist Poland

### The evolution of income inequality, 1983–2015

In this section we present the evolution of the full income distribution in Poland during the transition from communism to a market economy. This was a period of strong economic growth, re-industrialization and integration into the global economy. Poland has narrowed the income gap with Western Europe (Fig. 13), and today is considered by the World Bank as a high-income country.

Our new series on the evolution of income inequality in Poland show that official survey-based measures strongly underestimate the level of income inequality in Poland. In the same manner, our results suggest a notably higher increase in income inequality in Poland since the end of communism until today. The largest increase in income inequality occurred in the early 1990s, particularly between 1993 and 1995. The top 10 percent income share increased from levels around 22–23% in the 1980s to 25% in 1992–1993, and then jumped to 30% by 1995 (Fig. 2). This rise was accompanied by a fall in income shares of the middle 40 and of the bottom 50 percent. These groups experienced a roughly commensurate fall in income shares of around 5 percentage points between 1989 and 1995. Subsequently, we observe a steady rise in inequality, especially between 2003 and 2008, which has also been induced by the rising share of the top decile.

**Fig. 6:**
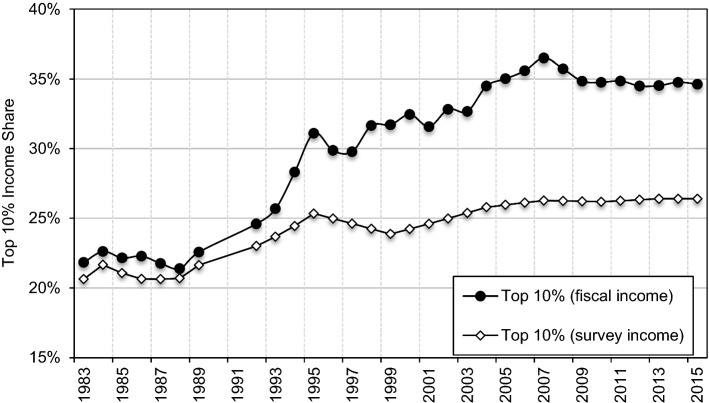
Top 10% Incomes Share in Poland, 1983–2015. *Source*: Authors’ computation. Survey estimates based on HBS (see Sect. 3). Distribution of pre-tax national income (before taxes and transfers, except pensions and unemployment insurance) among equal-split adults

It is important to note that this rise has been altogether overlooked by the official survey-based measures (Fig. 6). Between 1989 and 2015, the top 10 percent income share rose from levels slightly above 20% in the 1980s up to 35% in 2015, as opposed to around the 26% suggested by surveys. In the same period, the top 1 percent income share more than tripled, rising from around 4% to 13%, as opposed to the 6% suggested by surveys (Online Appendix Figure OA13).^49^ Overall, these results show the importance of correcting the upper end of the distribution in the survey data.

We next consider distributional effects of the transition in Poland by looking at the growth experience of different income groups. Over the 1989–2015 period, average real national income per adult has increased by 73%, or at about 2.1% per year. Overall, there has been a notable increase in the living standards of the Polish population since the fall of communism (especially when the grave stagnation of the 1980s is taken into account). However, this growth has not been equally shared. Table 2 shows that real incomes of the top 10 percent increased by 190% (or 4.2% per year) and of the top percentile by 458% (or 6.8% per year). On the other hand, the income growth of the bottom 90 percent has been much more modest: the bottom 50 percent experienced a 31% increase (1% per year) and the middle 40 percent a 47% increase (1.5% per year) in their real income. The finding that real incomes of the Polish bottom 50 percent have increased, but at the relatively lower rates, is consistent with the finding of Milanović and Ersado (2012) that in the former transition countries growth has been disequalizing in relative terms but increased absolute incomes of the bottom deciles. Table 2 also shows that the top 1 percent has captured almost twice as large a portion of the total income growth as compared to the bottom 50 percent group (24% versus 13%, respectively).

**Table 2:** Income growth and inequality in Poland 1989–2015

Income group (distribution of pre-tax national income) (%)	Average annual real growth rate 1989–2015 (%)	Total cumulated real growth 1989–2015 (%)	Share in total macro growth 1989–2016 (%)
Full Population	**2.1**	**73**	**100**
Bottom 50%	**1.0**	**31**	**13**
Middle 40%	**1.5**	**47**	**30**
Top 10%	**4.2**	**190**	**57**
Top 1%	6.8	458	24
Top 0.1%	9.7	1019	9
Top 0.01%	13.0	2273	3

Distribution of pre-tax national income (before taxes and transfers, except pensions and unemployment insurance) among equal-split adults. *Source:* Authors’ computation (see Sect. 3)

### Accounting for the rise of inequality after 1989

In this section, we discuss (in chronological order) the most important forces driving the rise of inequality after 1989. The ‘return’ to a market economy may be seen as a reversal of the process of communist equalisation, manifested in comprehensive labour market liberalization and the privatization process. Economic forces consequently assumed a more important role in determining inequality, yet their distributional impact was critically mediated in a complex interaction with the specific institutional and political context. We show that a technology-driven demand for an educated workforce and globalisation contributed to the growing earning and income inequalities. However, an even stronger rise in inequality during the transition was precluded by extensive social transfers, higher minimum wage or more gradual privatization.

Rising earnings dispersion has been commonly identified as the main cause of rising income inequality in Central Eastern European countries during the first years of transition (Flemming & Micklewright, 2000; Milanović, 1998; Mitra & Yemtsov, 2006). The rise of earnings dispersion in Poland can be clearly seen in Fig. 5 above. The rising educational premium, triggered by the decentralisation of the wage setting process, has been usually singled out as the main cause of rising wage inequality in Poland (e.g. Keane & Prasad, 2006; Letki et al., 2014; Rutkowski, 2001).^50^ In Appendix 2, we look jointly at the trends in the relative returns to education and the supply of educated workers, and infer about trends in the relative demand for educated workers (Goldin & Katz, 2008). Indeed, the university wage premium sharply increased from 35% in 1986 to 107% in 2007, which, together with the continual increase in the supply, implied a strong growth in the demand for educated workers, especially from emerging sectors such as financial, business and IT services. However, after the mid-2000s the supply growth outpaced the demand shifts and the wage premium declined to 78% in 2016. Consequently, wage inequality stagnated, although income inequality continued to grow (see Fig. 5, and Online Appendix Figures OA9-OA12).

Overall, a strong increase of educational attainments in Poland has often been singled out as the critical factor for the successful Polish transition. Consistent with the ‘modern channel’, lower inequality at the outset of the transition, with binding credit constraints, was conducive to the documented large expansion of tertiary education, which has been critical for the country to successfully adapt to the global environment and to approach the technological frontier (Galor & Weil, 2000; Piątkowski, 2018).

More extensive and well-targeted social transfers toward low-income groups and a generous minimum wage have often been seen as the most important mechanism in precluding a sharp rise in inequality in Poland during the transition (Keane & Prasad, 2002; Mitra & Yemtsov, 2006).^51^ In this respect, the contrasting development of the bottom 50% of income shares in Poland and Russia is particularly striking (Fig. 20). The bottom 50% share was around 30% of national income in both countries in the 1980s. But, while the bottom 50% share in Russia more than halved between 1991 and 1996, its Polish counterpart experienced a relatively moderate decline during the same period. This development mirrors a divergent development of institutional and political factors during respective transitions in Poland and Russia, in particular, of transfer payments and minimum wage (see Fig. 19 and Online Appendix Figure OA15). Moreover, Keane and Prasad (2002) have argued that these social policies ensured social stability and provided the general political support for the market reforms and enterprise restructuring in Poland.^52^ Further, the different privatization strategies pursued—‘big-bang’ in Russia versus more gradual in Poland—have often been highlighted as an important source of the documented divergence of income and wealth inequality trajectories in two countries.^53^

**Fig. 7:**
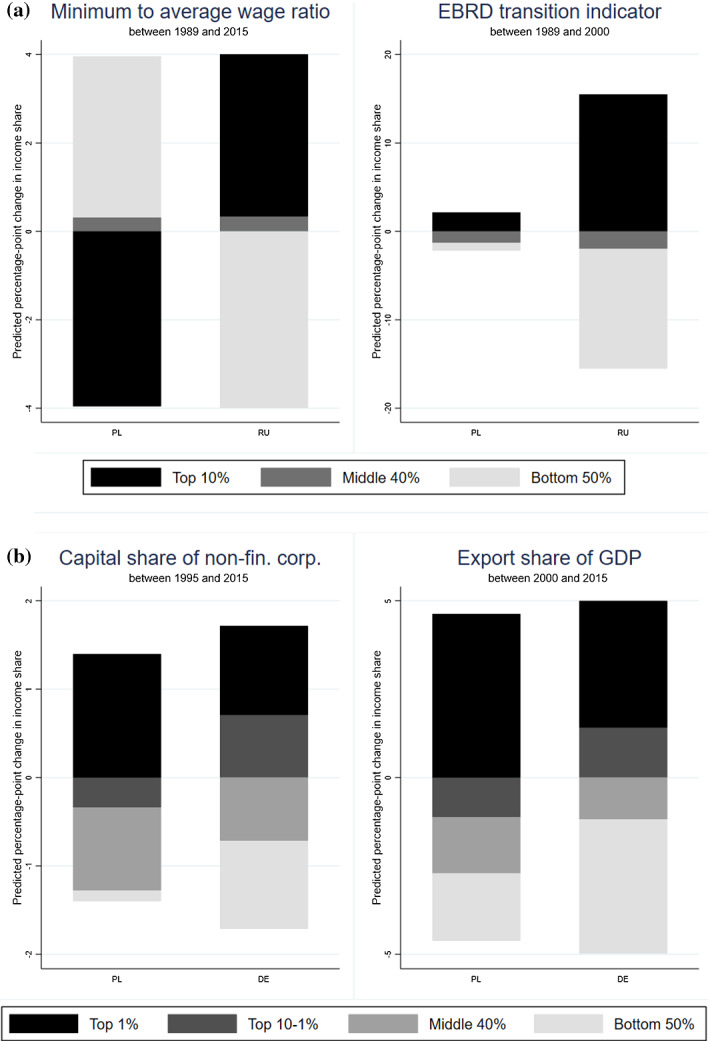
**a** The predicted (non-causal) effect of the cumulative change in minimum wage and institutional transition on income shares in Poland and Russia. **b** The predicted (non-causal) effect of the cumulative change capital share and export share on income shares in Poland and Germany. *Source*: EBRD transition indicator: EBRD. Minimum wage ratio, capital share and export share: OECD. Income share: authors’ computation and WID. Distribution of national income among equal-split adults. Note: the upper left (right) panel shows the estimated non-causal change in income shares due to the actual change in minimum wage to average wage ratio (EBRD transition indicator) between 1989 and 2015 (2000). The bottom left (right) panel shows the estimated non-causal change in income shares due to the actual change in capital share (export share) between 1995 (2000) and 2015. The estimates are constructed from country-specific correlation coefficients between the analysed factor and each income share, estimated from a panel fixed effect country-level regression (PL, CZ, HU, RU (upper panel), DE (bottom panel)), which includes a full set of year fixed effects. The bars are calculated by multiplying the estimated coefficients for each income share and country by the actual change in the independent variable. Online Appendix Figure OA18 presents the results for Czech Republic and Hungary

We provide suggestive evidence for the differential role of minimum wage and privatization for the evolution of inequality in Poland and Russia. We construct a panel of four countries (Poland, Russia, Hungary and Czech Republic) from the early 1990s until 2015. Next, we regress income shares (top 10%, middle 40% and bottom 50%) on minimum to average wage ratio or the EBRD privatization indicator, and a set of country fixed effects and year fixed effects, which together capture all country-specific time-invariant determinants of inequality and all common annual shocks to income shares. Figure 7a presents the estimated non-causal change in income shares due to the actual change in the minimum to average wage ratio (the privatization indicator) between 1989 and 2015 (2000)^54^ (i.e. we estimate the effect of 1 pp increase of the independent variable and multiply it by the change of this variable between 1989 and 2015/2000). For the sake of brevity, we report only results for Poland and Russia (see Online Appendix Figure OA18 for the results for Czech Republic and Hungary). The results show that the actual changes in minimum wage in these two countries have opposite non-causal predictions for the evolution of inequality. The rise of minimum wage in Poland, *ceteris paribus*, is associated with the rise of bottom 50% by 3.6 pp, middle 40% by 0.4 pp and analogous fall of top 10% by 4 pp. In Russia, the predicted changes are opposite and show a 4 pp fall of bottom 50%. The predicted change in income shares due to the actual changes in privatization are also in line with our expectations. Strikingly, the rise of the indicator between 1989 and 2000 predicts a rise of top 10% by 2.1 pp in Poland, but more than 15 pp in Russia, and a fall of bottom 50% by 0.9 pp and 13.5 pp respectively. Overall, a markedly different transition experience in Poland and Russia suggests that there was no predetermined trajectory of inequalities during the transition. It clearly shows that policies and institutions play an important role in shaping inequality.

**Fig. 8:**
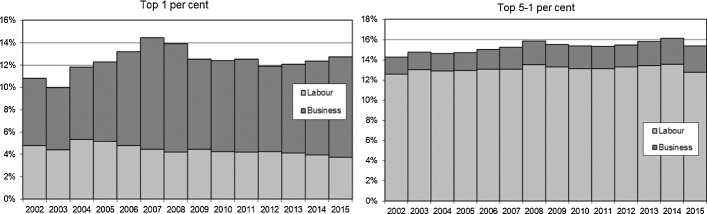
Top 1 and Top 5–1% income decomposition between business and labour income, 2002–2015. *Source*: Authors’ computation based on income tax statistics; Distribution of fiscal income among tax units. Note: labour income includes income from employment, pensions, as well as other non-business income sources

The recent rise in inequality in Poland has been driven again by the increase in top income shares. The top 10 percent share has steadily increased since the early 2000s and reached levels of around 35% by 2015 (Fig. 2). To understand better this development, we turn to the income composition for top groups. Figure 8 shows that higher top income groups have been mostly composed of business income, while earnings have dominated for lower top groups constituting the top decile, such as for the ‘next’ 4%. Business income combines both the capital and the labour component (Kopczuk & Zwick, 2020),^55^ but in the Polish context, it is plausible that business income at the top reflects in large part the return to capital (Alstadsæter et al., 2017; Kopczuk, 2012; see Sect. 3).

A robust rise in top business incomes after 2004 was the main reason behind the structural rise in top income shares.^56^ This also calls for a more detailed study of the effect of factor shares (Fig. 18). The period after the EU accession has been associated with capital deepening (Gradzewicz et al., 2014) and rising capital share (Growiec, 2012). With the top 1 percent income group holding almost two-thirds of the total fiscal business income, any notable change in the functional distribution towards capital could result in rising top concentration (e.g. Atkinson, 2009). This observation is confirmed by a regression of income shares on capital share. We construct a panel of four countries (Poland, Germany, Hungary and Czech Republic) for the period between 1995 and 2015, and regress income shares on capital share,^57^ and a set of country fixed effects and year fixed effects. The left side of Fig. 7b reports the estimated non-causal change in income shares, which is attributed to the actual change in capital share between 1995 and 2015. For the sake of brevity, we report only results for Poland and Germany. If the correlations were causal, *ceteris paribus*, the rise of capital share in Poland would imply the rise of top 1% by 1.4 pp and the fall of all other income groups.

The rise of capital share has often been attributed to the new globalization phase and increasing participation in international trade. We point to three broad channels concerning how globalization might have potentially induced the rising capital share in Poland.^58^ The first channel is the capital-augmenting technological change, which entered Poland through strong foreign direct investment (FDI) after the EU accession (Olszewski, 2009). The second channel is a trade-induced shift towards capital-intensive sectors. Traditional labour-intensive industries, such as mining or textile manufacturing, have been in decline due to the increasing trade competition, especially after China joined the WTO in 2001 (Balsvik et al., 2015; Growiec, 2012). Finally, the rising market power of multinational companies could have also contributed to the falling labour share (Autor et al., 2020; Bell et al., 2018; Benmelech et al., 2018). We provide suggestive evidence for the role of globalisation in the right side of Fig. 7b. We employ a similar method as in the case of capital share, except we regress income shares on the export share of GDP and a full set of country and year fixed effects. The actual rise of export share between 2000 and 2015 predicts (in a non-causal fashion) a strong and statistically significant increase of the top 1% by 4.6 pp, accompanied by a fall of all other income groups.

The distributional effects of globalization and technological change can also explain a decline in the income share of the middle 40% (Fig. 2), which can be attributed to the relative standing of the Polish middle class. It has been documented that middle-skilled jobs in developed economies are more likely to have been automatized, leading to so called ‘job polarization’ (Autor et al., 2006). At the same time, employment in manufacturing—a traditional sector of the middle class—has been in decline due to offshoring and trade competition with developing countries (Autor et al., 2016). The relative fall in the importance of the middle class, which is the ‘back-bone’ of democracy, might be related to the recent rise of populism across the CEE and internationally (Easterly, 2001; Lindner et al., 2020, etc.).

## International comparison

**Fig. 9:**
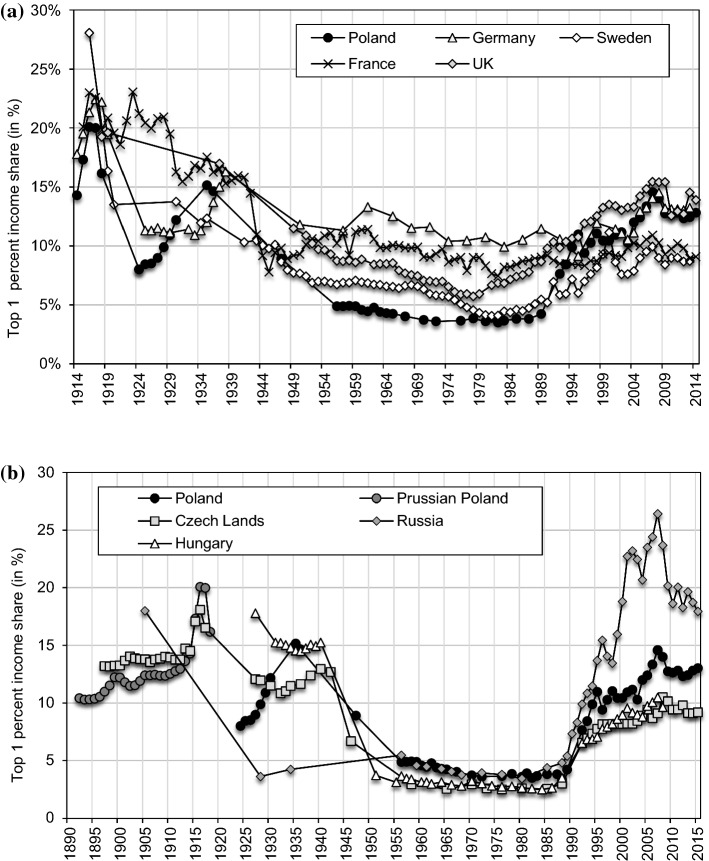
**a** Top 1% in Poland, Germany, France and Sweden, 1914–2014. *Source*: Poland: authors' computation based on income tax statistics. Distribution of fiscal income among tax units. Other countries: WID.world. Note: 1914–1919 for Poland refers to the Prussian Poland. **b** Top 1% in former communist countries, 1890–2015. *Source*: Poland: authors' computation based on income tax statistics. Distribution of fiscal income among tax units. Other countries: Mavridis & Mosberger, 2017; Novokmet et al. 2018, Novokmet, 2018, WID.world.

**Fig. 10:**
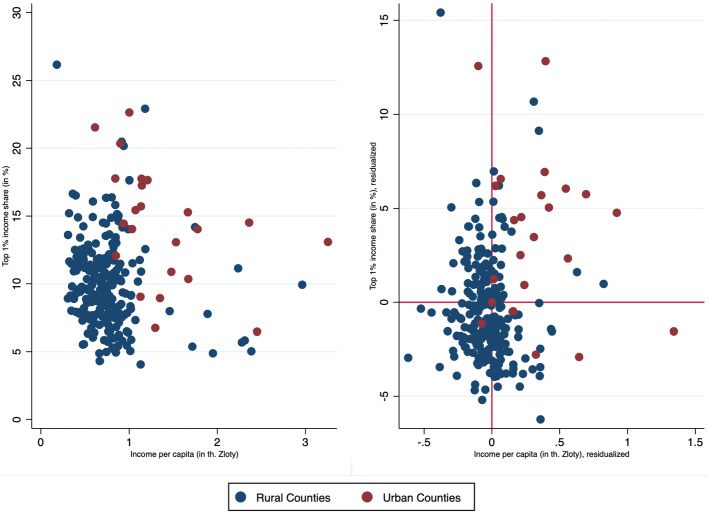
County-level income per capita and the top 1% income share, 1927. *Note*: The right panel shows residuals from the regressions of Top 1% income share or income per capita on a set of 17 regional fixed effects. Rural (urban) counties have urbanization rate below (above) 38%. Source, see Online Appendix OA1.5

**Fig. 13:**
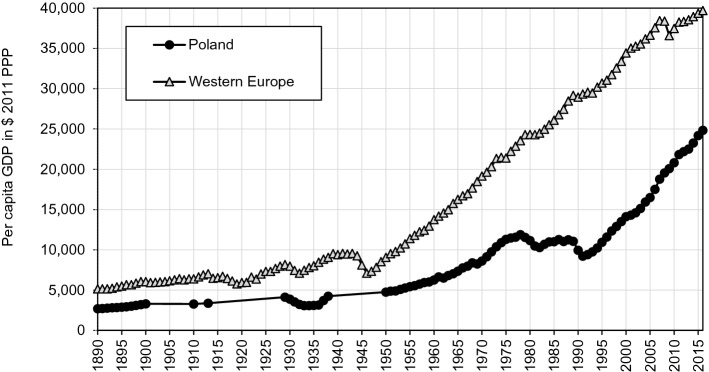
Real GDP per capita in Poland and Western Europe, 1890–2016. *Source*: Authors’ computation based on Bolt et al. (2018). Western Europe is the unweighted average of Germany, France and UK

**Fig. 15:**
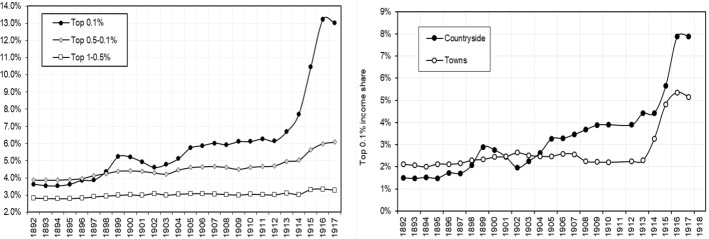
Top income shares in the Province of Posen, 1892–1917. The left panel shows the decomposition of the top 1 percent income share. The right panel presents the breakdown of the top 0.1 percent income share between countryside and towns. *Source*: Authors’ computation based on income tax statistics

**Fig. 16:**
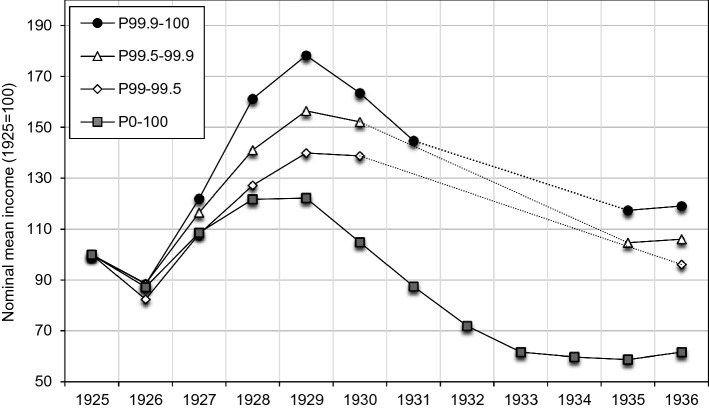
Evolution of average income of groups within the top percentile, 1925–1936. *Source*: Authors’ computations based on the income tax data

**Fig. 18:**
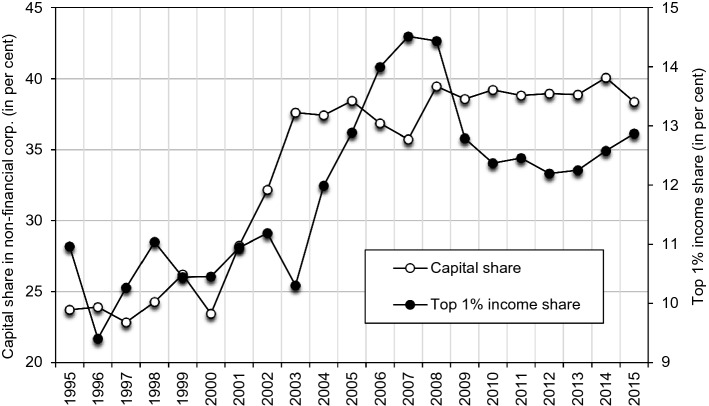
The evolution of capital share in gross value added of non-financial corporations and the top 1% income share, Poland 1995–2013. *Source*: Capital share: Polish National Accounts; Eurostat. Top 1% income share: authors’ computation based on income tax statistics. Distribution of fiscal income among tax units Note: capital income is calculated as 80 per cent of gross operating surplus in non-financial corporations. Capital share is the proportion of thus obtained capital income in factor-cost gross value added of non-financial corporations. We take 80 percent of gross operating surplus since Polish National Accounts place unincorporated enterprises with more than 10 employees in non-financial sector, and we assume that the part of its operating surplus should be attributed to labour income of owners and household members. The obtained estimates of capital share in GVA are consistent with those of Karabarbounis and Neiman (2014)

**Fig. 19:**
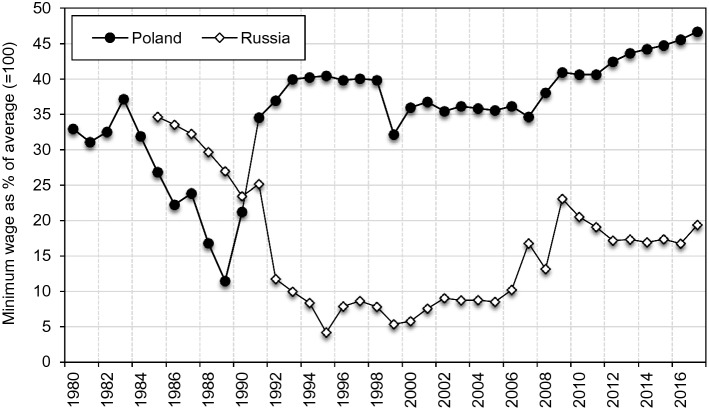
Minimum wage to average wage ratio in Poland and Russia, 1980–2017. *Source*: Minimum wage to average wage ratio: OECD and author’s computation from Statistical yearbook of Poland and Statistical yearbook of Russia

We next turn to international comparisons. First, we compare the long-run evolution of income inequality in Poland and Western European countries. Figure 9a shows the evolution of the top 1% share in Poland, Germany, France, Sweden and the UK between 1914 and 2014. A broad U-shaped pattern can be observed in all countries throughout the twentieth century, but it has been especially pronounced in Poland. The period between 1914 and 1945 initiated a secular downward inequality trend worldwide due to wars, and political and economic shocks. While WW1 itself was advantageous to top incomes in presented countries, the immediate post-war development hit top incomes hard, with a particularly pronounced decline in Poland. It is now well documented that the evolution of top income shares in developed countries during the first half of the twentieth century reveals the fate of top capital incomes (Atkinson & Piketty, 2007; Atkinson et al., 2011).

While inequality stood at low levels in all countries in the three post-WW2 decades, the introduction of communism dramatically reduced top income shares in Poland and kept them below the levels observed in western European countries. With the fall of communism, the top percentile share substantially increased in Poland between 1989 and 2015, to reach the levels characteristic for more unequal European countries, notably Germany and the UK, and placed Poland significantly above estimates for the group of Continental, Nordic and Southern European countries (e.g. Atkinson et al., 2011). It is interesting to point to the recent similar evolution of the top percentile share in Poland and Germany.^59^

**Fig. 20:**
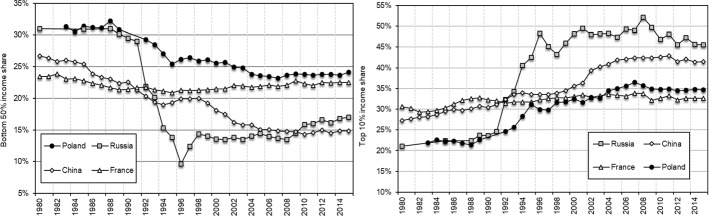
Bottom 50 (left) and 10 (right) per cent in Poland, China, France, and Russia, 1980–2015. *Source* for Poland: Authors’ computation (see Sect. 3). Distribution of pre-tax national income (before taxes and transfers, except pensions and unemployment insurance) among equal-split adults. Other countries: WID. world

Next, it is of particular interest to compare the experience of Poland to that of other ex-communist countries in Eastern Europe. Figure 9b shows that all countries for which we have historical inequality series—Poland, Hungary, the Czech Republic and Russia—have displayed a marked U-shaped evolution of top income shares in the long-run (Mavridis & Mosberger, 2017; Novokmet, 2018; Novokmet et al., 2018). It can be seen that the introduction of communism sharply reduced top income shares in all countries. However, the return to the market economy saw quite divergent development of inequality in Russia in comparison to countries in Central Eastern Europe. The top percentile share in Russia surged to levels of around 20%, while in the latter countries it stabilised at levels of between 9% and 14%—with Poland at the upper end of the spectrum and the Czech Republic and Hungary at the lower end.^60^ Similar conclusions about the post-communist distributional dynamics emerge from a comparison of the evolution of the bottom 50 percent and the top 10 percent income share in Poland and Russia (Fig. 20). As discussed above, a divergent evolution of income inequality in Poland and Russia is observed both at the lower and upper parts of the distribution, which we link to different institutional and political factors during countries’ respective transitions.

Overall, the Polish experience in the international perspective points to the central role of policies and institutions in shaping inequality in the long run, as made clear by the unparalleled changes in the labour market and capital ownership arrangements which have accompanied the rise and fall of communism. To a certain extent, the evolution of inequality in Poland may be seen as an extreme version of inequality developments in Western countries. In the latter, authors such as Piketty (2001, 2014) or Atkinson (2015) have attributed the key role to fiscal institutions and redistributive policies (such as changing patterns of progressive taxation and social expenditure; see also Lindert, 2004) for the long-run evolution of income inequality. A broad synchronisation of top income shares in communist and non-communist countries in the long run presents suggestive evidence against the ‘natural’ decline of inequality along the development path. Furthermore, divergent transition strategies and inequality trajectories in Poland and Russia support the importance of institutions and policies as drivers of inequality (Keane & Prasad, 2002; Novokmet et al., 2018). Finally, the comparative historical account provides a unique perspective to investigate the relative importance of different factors in determining inequality.

## Conclusion

This paper has combined tax, survey and national accounts data to provide the first consistent series on the long-term income distribution in Poland. We draw a U-shaped evolution of income inequality in Poland throughout the twentieth century.

This study shows that the evolution of inequality is critically shaped by the inextricable workings of economic, social and political forces. The Polish inequality history clearly suggests that that the secular fall in inequality in the first half of the twentieth century was not a necessary feature of the development process, but equally that the rise of inequality is not inevitable. Dramatic changes in top income shares during the rise and fall of communism in Eastern Europe indicate the central role of institutions and policies in shaping long-term inequality patterns.

We also point to the role of economic forces (e.g. technological change and globalization) as determinants of inequality but argue that their distributional impact depended on a wider country-specific institutional and socio-political context. These processes have also recently contributed to the growing importance of capital in the economy, suggesting that the future of inequalities in Poland is likely to be linked with the prominent role of capital income at the top. Yet, our paper shows that these trends are not inevitable and the future inequality dynamics in Poland will crucially depend on institutional and policy choices.

Rising income inequality in Poland has important political and social implications. It shows that the official numbers on the average GDP growth might have little in common with the actual lived experience of most people. Those “left behind” are often missing from the public discourse, which further fuels the illusion of inclusive growth and limits the demand for social policies. As a result, inequality stands today at the heart of the democratic debate in Poland and worldwide, which is evident in the recent populist backlash in Poland and internationally. The issue of distribution of gains from economic growth has thus become crucial for sustaining the long-run development. We hope that our work will be a contribution to how to approach these imminent challenges.

Finally, this paper shows promising avenues for future research. Most importantly, our work presents the central building block for constructing comprehensive Distributional National Accounts for Poland (Bukowski et al., 2021). We emphasize several extensions directly building on our work. First, it is important to produce both the pre-tax and post-tax income distribution, to assess the redistributive role of the government. Next, the new distributional statistics should also provide insights into the distribution of different socio-economic dimensions such as gender, age, region, etc. As pointed out in the paper, it would be especially useful to provide the breakdown by labour and capital income for the whole income distribution. This could shed light on the role of different economic mechanisms in the inequality dynamics, such as international trade or automation. Lastly, the high concentration of business income in Poland calls for further research on the relative importance of human vs. non-human capital at the top of the income distribution, as well as its future implications for growth and inequality (Kopczuk & Zwick, 2020; Piketty, 2014; Smith et al., 2019). Relatedly, there is a need to look at income and wealth distribution together (Kuhn et al., 2018; Piketty et al., 2018).

### Supplementary Information

Below is the link to the electronic supplementary material.

Supplementary file1 (PDF 1945 kb)

## Figures and Tables

**Fig. 11:**
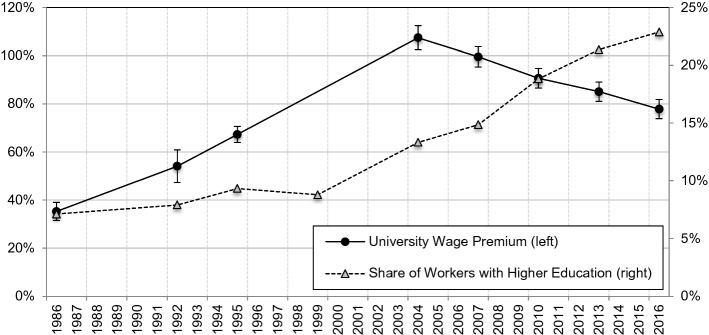
The returns to higher education and the supply of university graduates, 1986–2016. *Source*: own calculation based on the LIS data. *Note*: The sample consists of adult male employed workers. The university wage premium is the coefficient on a college dummy from the regression of a natural logarithm of annual labour income on the dummy and a second order polynomial of potential experience. The bars denote the 95% confidence intervals. We exclude 1999 because the data does not allow us to separate wage income from total income. The share of workers with higher education is a population-weighted average of the college dummy

**Fig. 12:**
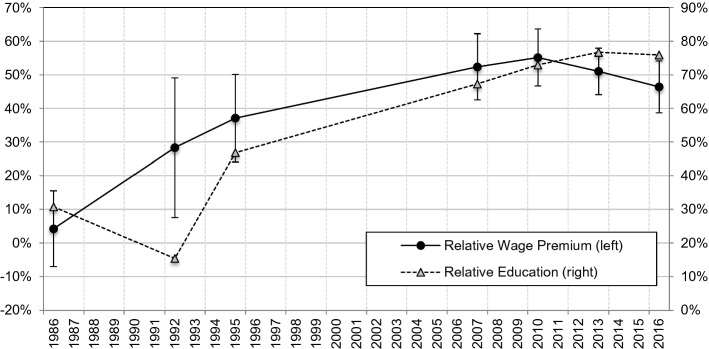
Wage premium and relative education in the finance sector, 1986–2016. *Source*: Own calculation based on the LIS data. *Note*: The sample consists of adult male employed workers. The relative wage premium is the coefficient on a finance intermediation industry dummy from the regression of a natural logarithm of annual labour income on the dummy. The bars denote the 95% confidence intervals. We exclude 1999 and 2004 because the data does not allow us to separate financial intermediaries from business services. The relative education is a population-weighted average of the college dummy among male workers employed in the financial sector

**Fig. 14:**
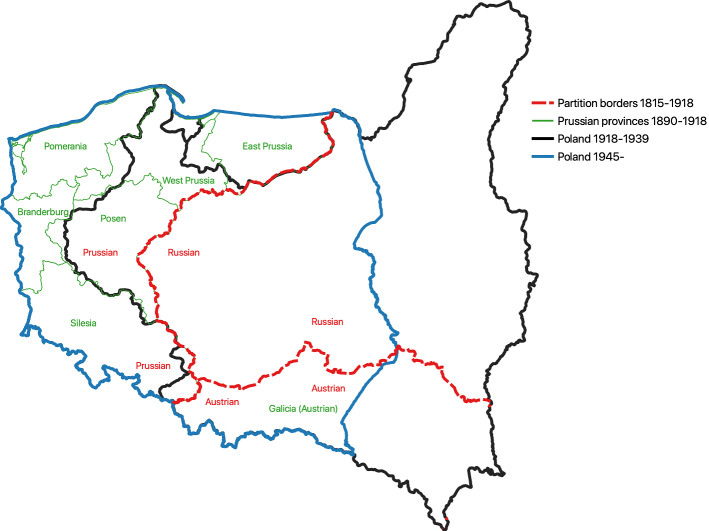
The borders of Poland and the Partitions

**Fig. 17:**
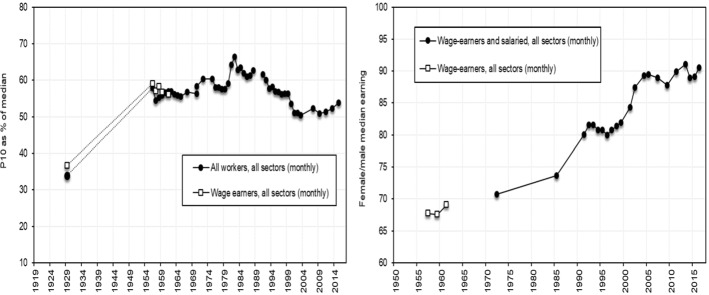
Long-run evolution of the bottom part of the earnings distribution (left) and the development of the gender earnings gap (right), 1929–2015. *Source*: Own construction from sources in Fig. 5; 1972 and 1985 from Atkinson & Micklewright, 1992, T.PE3

## Appendix 1: Interwar county-level analysis

In this appendix, we shed light on the determinants of income inequalities in interwar Poland and the consequences for the aggregate top incomes. First, we analyse the determinants of the county-level income inequalities. Second, we study a connection between the national-level top income and the local-level top income share.

### Looking for the Kuznets curve in interwar Poland

The natural starting point is to look at the relationship between inequality and economic development. Simon Kuznets—born in 1901 in Pińsk, a city located in the independent Poland after 1918 (today in Belarus)—formulated the famous inverted-U curve, according to which inequality rises in early phases of economic development but falls eventually as the growth advances (Kuznets, 1955). We test this prediction using self-constructed cross-sectional data on counties located in Poland in 1927. In particular, we regress the top 1% income share on a second order polynomial of income per capita, a set of socio-economic variables and 17 regional fixed effects. The Online Appendix OA1.5 provides the details on the data construction and variables.

Table 3, Column 1 shows that there is no relationship between the top 1% income share and income per capita in a simple univariate regression. This could be due to unobservable differences between regions (*województwo*). Column 2 controls for them by adding regional fixed effect, consequently the coefficients become significant and imply a U-shaped relationship between the level of economic development and the top 1% income share. This effect can be influenced by omitted variables. The inclusion of additional covariates might be misleading, however, if the variables are potential outcomes of economic development. If this is the case, one might switch off a potential channel through which income per capita affects inequalities. Keeping these concerns in mind, in Columns 3 and 4, we control for urbanization rate, literacy rate, the Gini coefficient of land ownership, the share of agriculture workers in total employment and the share of industrial workers in total employment. The coefficients on the polynomial of income per capita have now larger magnitude and are strongly significant,^61^ but still showing a U-shaped relationship. On the other hand, inequality is positively related to urbanization, land inequality and employment share in agriculture and industry.

**Table 3:** County-level regressions of the top 1% income share, 1927

Partition:	Top 1% Income Share (in %)								
	All			Prussian		Russian		Austrian	
	(1)	(2)	(3)	(4)	(5)	(6)	(7)	(8)	(9)
Income per capita	− 0.20	− 0.94	− 4.95***	3.25	0.45	− 1.29	− 5.82*	− 12.99***	− 4.13
	(0.89)	(1.28)	(1.22)	(2.49)	(3.67)	(3.98)	(3.12)	(3.88)	(2.94)
Income per capita^2^	0.08	1.68*	2.57***	− 0.95	0.23	1.82	2.90	25.61***	− 1.02
	(0.83)	(1.01)	(0.81)	(1.46)	(1.99)	(4.19)	(2.56)	(8.87)	(5.76)
Urbanization	–	–	1.75***	–	2.33***	–	1.27**	–	2.63***
			(0.32)		(0.51)		(0.60)		(0.60)
Literacy rate	–	–	− 0.11	–	− 1.32	–	− 1.05	–	0.03
			(0.51)		(4.51)		(1.28)		(0.49)
Land Gini	–	–	− 0.14	–	0.83*	–	− 0.84	–	− 0.46
			(0.31)		(0.47)		(0.61)		(0.38)
Share of agr. workers	–	–	2.14***	–	1.38**	–	3.28***	–	− 0.26
			(0.39)		(0.64)		(0.86)		(1.06)
Share of ind. workers	–	–	2.24***	–	0.60	–	3.31***	–	2.00***
			(0.54)		(0.94)		(1.06)		(0.70)
Constant	10.44***	9.94***	10.74***	13.54***	12.68*	9.89***	10.75***	14.11***	9.77***
	(0.22)	(0.85)	(0.77)	(0.67)	(6.62)	(0.90)	(1.09)	(1.44)	(0.86)
R-squared	0.00	0.24	0.48	0.41	0.62	0.09	0.40	0.08	0.56
Counties	256	256	256	57	57	125	125	74	74
Regional FE	No	Yes	Yes	Yes	Yes	Yes	Yes	Yes	Yes

The table shows correlations from a regression of county-level top 1% income share on a second order polynomial of income per capita, urbanization rate (share of population living in cities), share of agriculture workers in total employment, share of industrial workers in total employment, literacy rate (share of people who can read and write), Gini coefficient of land ownership. In addition, in columns 2 to 8 seventeen regional fixed effects (*województwo*) are included. See Online Appendix OA1.5 for more on data sources. Robust standard errors in the parentheses. *** denotes significance at the 0.1% level, ** at the 1% level and * at the 5% level

Columns 4 to 9 report the regressions for each of the former partitions separately. The effect of income per capita is heterogeneous: insignificant in the former Prussia and Russia, and U-shaped in the former Austria. Controlling for other covariates renders insignificant relationships. In all partitions, we report a positive and significant coefficient on urbanisation rate. The positive and significant coefficients on Gini of land distribution and the employment share in agriculture in the case of former Prussian counties are consistent with the primary role of ‘agrarian capitalism’ (as opposed to industry) in shaping inequalities. On the other hand, in the former Austrian counties, the coefficient on the employment share in industry is significant and positive, while land inequality and employment in agriculture are irrelevant. Similarly, in the former Russian partition industrialization and urbanization are positively associated with inequalities. Interestingly though, like in the Prussian partition, the share of workers employed in agriculture has also a positive, significant and high coefficient.

Simple cross-sectional regressions of top income shares on income per capita do not confirm the predictions of Kuznets (1955). If anything, we document a U-shaped relationship between inequality and economic progress. On the other hand, the positive effects of urbanisation and the industry share of workers are consistent with the Kuznets’ predictions. These results are generally in line with the more recent literature (for a review, see Bourguignon, 2005; Modalsi 2018). Our approach alleviates some concerns from the cross-country regressions, as the analysed counties were part of independent Poland in 1927 and thus were affected by the same nation-level economic and political shocks. Nevertheless, the cross-sectional nature of our data does not allow us to fully control for the fixed county characteristics.

Finally, can we conclude that the Kuznets model does not apply to Interwar Poland? First, it should be kept in mind that the rise of inequality in the Kuznets curve stems partially from a growing gap in income per capita *between* urban and rural areas (or sectors). Focusing on *within*-county inequalities might therefore miss this dimension. One prediction of the Kuznets’ stylized two sector model is that, in the initial phase of economic development, economic activity shifts to cities (due to higher productivity), which benefits owners of capital and induces migration from rural areas. Thus, both a shift of income in favour of capital owners and the increased supply of unskilled workers aggravate inequality in the urban sector. Consequently, both inequality and income per capita should be higher in cities. On the other hand, the paradigm model assumes that rural areas are relatively poorer and less (or at least as) unequal. The left panel of Fig. 10 presents a scatter plot of income per capita and top 1% income share for two groups of counties: with urbanization rate below and above 38% (90th percentile).^62^ The urban counties are on average richer than the rural ones (1,384 vs. 754 zł.), and they also tend to be more unequal (14.3% vs. 10%). This could be due to historical differences in levels of inequality, prosperity and urbanization rates across regions. The right panel of Fig. 10 shows the county-level top 1% income share and income per capita relative to the region’s mean (*województwo*). Most of the urban counties are located in the upper-right quarter of the figure, that is, they have relatively higher income and inequality compared to nearby rural counties.

Overall, a more nuanced look at the relationship between inequality, economic development and urbanisation reveals patterns broadly consistent with the predictions of the upward part of the Kuznets curve. This may suggest that Poland in the interwar period was undertaking a modest structural change. However, further work is needed in this direction and a particular consideration should be given to the specific historical context of Poland.

Several qualifications are in order in this respect. Importantly, it needs to be recognized that the actual historical setting did not necessarily conform to the main assumptions of the Kuznets model (e.g. see Kaelble and Thomas 1991). First, it is not clear that the agricultural sector was always backward and less productive and that modern growth is necessarily associated with the shift of economic activity away from agriculture. Economic development in the Prussian partition is a clear example of where ‘modernization’ is rather related to the emergence of productive (‘commercial’) agriculture (e.g. the ‘Prussian road to industrialization’ (Lenin, 1908); for example, most rural counties on the left panel of Fig. 10 with above-average income per capita (e.g. above 1 th. zł.) are in the former Prussian partition). Second, the intra-sectoral inequality might be different than assumed in the Kuznets’ setting. Namely, one of the model’s stylized facts is that the agricultural sector is relatively more equal, yet very high inequality is documented in the rural areas in the Prussian (and partly in the Russian) partition because of very high land concentration (see Sect. 4.1). Finally, and most importantly, the ability of alternative inequality indices to capture the Kuznets process needs to be borne in mind (see generally Anand & Kanbur, 1993). Kaelble and Thomas (1991, p. 14) point out in this respect that top shares might not be an ideal measure to identify distributional effects of development.

### Contribution to the national top 1% income

In the second part of this appendix, we use the cross-sectional county-level data to analyse determinants of contribution to the aggregate national top 1% income. First, we explore the relationship between the county-level contribution to the national top 1% income and the county-level total income. Table 4, Column (1) below, shows a positive and significant correlation—standard deviation increase in the total income is associated, on average, with 1.4 pp increase in the contribution (the unconditional mean is 0.39%). As a next step, we introduce to the regression county-level population and the county-level top 1% income share (Columns (2) and (3)). The coefficient on the population size is basically zero, which is not surprising given that the number of rich is more likely to be a function of the total income. The effect of the top 1% income share is 0.3 pp and highly significant. Controlling, in addition, for urbanization rate, literacy rate, Gini of land ownership, the share of workers in agriculture and industry, and 17 regional fixed effects does not significantly alter the results (Column (4)). Overall, we find that the most significant contributor to the national top percentile is the size of the local economy (e.g. Warsaw, Łódź and Lviv contribute with around 40% of all top percentile constituents). Interestingly, the results show that a strong connection between the national inequalities are within-counties inequalities. This opens up questions about the nature of this relationship.

**Table 4:** County-level regressions of the contribution to the national top 1% income, 1927

	Contribution to the national top 1% income (in %)			
	(1)	(2)	(3)	(4)
Total income	1.41***	1.47***	1.46***	1.5***
	(0.09)	(0.14)	(0.13)	(0.13)
Population	–	− 0.07	− 0.08	− 0.2
		(0.14)	(0.04)	(0.13)
Top 1% income share	–	–	0.3**	0.25***
			(0.1)	(0.07)
Urbanization	–	–	–	0.32**
				(0.12)
Literacy rate	–	–	–	− 0.05
				(0.06)
Land Gini	–	–	–	0.04
				(0.07)
Share of agriculture workers	–	–	–	0.09
				(0.06)
Share of industrial workers	–	–	–	− 0.18**
				(0.06)
Constant	0.39***	0.39***	0.39***	0.4**
	(0.09)	(0.05)	(0.03)	(0.12)
R-squared	0.83	0.83	0.87	0.9
Counties	256	256	256	256
Regional FE	No	No	No	Yes

The table shows correlations from a regression of county-level contribution to the national top 1% income on total income, population size, within-county top 1% income share, urbanization rate (share of population living in cities), share of agriculture workers in total employment, share of industrial workers in total employment, literacy rate (share of people who can read and write), Gini coefficient of land ownership. In addition, in column 4 seventeen regional fixed effects (*województwo*) are included. See Online Appendix OA1.5 for more on data sources. Robust standard errors in the parentheses. *** denotes significance at the 0.1% level, ** at the 1% level and * at the 5% level

## Appendix 2: The supply and demand of skills after the fall of communism

Following the classic 'demand and supply of skills' framework (e.g. Goldin & Katz, 2008), we assess the role of the demand for and the supply of university graduates in driving the rise on earnings inequality after the transition to capitalism. In particular, we jointly analyse trends in the relative returns to education and the supply of educated workforce (observable from the data) and conclude about trends in the demand for educated workers. To this end, we use representative individual-level data from the Household Budget Surveys from the Luxemburg Income Study (LIS) available from 1986 until 2016. We estimate a Mincerian regression of the logarithm of annual wages on a college dummy and a second-order polynomial of potential work experience. Because information on hours worked and type of employment is not available, we limit the sample to employed adult males only, who, compared to females, are more likely to work full-time. The measure of the supply of education—the share of workers with higher education—is calculated from the same sample.

Figure 11 presents the male university wage premium (relative to primary education) and the share of the male workforce with higher education. The wage premium rose continuously between 1986 and 2004 from 35 to 107%, and the share of university workers almost doubled from 7 to 13%. Such co-movement is possible if the rise of the supply was over-balanced by the even stronger rise of the demand for educated workers. In the period after 2004, however, the premium fell by 30 percentage points and the supply grew by 10 percentage points, implying that the demand for educated workers stagnated or rose at a smaller pace than the supply. The conclusions remain the same if one looks at the returns to an additional year of education and the average years of schooling (results available upon request).

The growth of the demand for educated workers during the 1990s and the early 2000s coincides with the parallel growth of income and wage inequalities (see Figs. 2 and 5). This is hardly surprising, as most of the hypotheses link the post-transition rise of inequality with the structural changes on the labour market, such as liberalization, privatization, financialization and technological change (Brzezinski et al., 2013; Keane & Prasad, 2002, 2006; Rutkowski, 1996, 2001). It has been stressed that the development of financial, business and IT services (fuelled by the inflow of FDI), contributed to the strong demand for educated workers. The sector of financial intermediaries serves as a telling example. It was of marginal importance during communism, with the employment of around 0.2% of the male workforce in 1986. The transition induced a rapid development of the financial sector: the employment share tripled by 1995 and continued to grow, reaching nearly 1.6% of the total male employment in 2016. This growth was strongly biased towards university graduates. The black dashed line in Fig. 12 shows that at the outset of communism, less than one-third of male workers in finance had a university degree. The share grew rapidly after the transition and today it has the level of 80%. The growing demand for financial specialists was clearly reflected in the rise of relative wages paid in this sector (compared to other sectors). As marked by the black solid line, during communism there was no monetary advantage for those working in finance, but the wage premium rose to 30% in 1992 and continued to grow until 2010 when it reached its peak at 55%. When the supply increase outweighed the demand shifts, the wage premium fell between 2010–16 by 9 percentage points.

The situation on the labour market changed after 2004, when the supply growth outpaced the demand and wage inequality stagnated (Magda et al., 2020). However, our series show that income inequality continued to grow, and we relate this development to the growing share of income accrued to business and capital owners. Therefore, one must look beyond labour income to understand the recent rise of income inequality.

## Appendix 3: The role of migration

This appendix explores the possible effect of migration on the evolution of inequality in Poland. Migration might influence income inequality through changes in the composition of population (‘quantities’) and/or through changes in the renumeration of factors (‘prices’) (e.g. Card, 2009). In the case of top income shares, an outflow of low-skilled workers will likely lower inequality, because there are fewer non-migrant low-skilled individuals in the country and their wages should rise. The effect of an outflow of high-skilled workers, on the other hand, is ambiguous. The composition effect reduces inequality, but it might be counterbalanced by the rise in wages of non-migrant high-skilled workers (but could reduce the income of capital owners). In what follows, we use this framework to qualitatively assess the likely effect of major migration episodes in Poland since the end of the nineteenth century on the domestic distribution of income.

The Austrian and Prussian partitions differed with respect to the selectivity of outmigration (Zubrzycki, 1953). The rural poverty in the former led to an economic emigration of poor farmers and agriculture workers, many of them moving permanently to North and South America and temporarily (as seasonal workers) to Prussia. Overall, nearly 1 million people left Galicia between 1890 and 1913, which constituted almost one-sixth of the population in 1890. The composition effect and the potential rise in wages of non-emigrants should lower inequality. This could be, however, counterbalanced by changes in the structure of land ownership. The outflow of small landholders decreased the demand for land and increased the supply, possibly facilitating the concentration of land ownership. Yet, the data from the land censuses show instead progressing parcellation (Mieszczankowski, 1960).

The economic situation in the Prussian partition was better; however, the active policy of Germanization led to a sizeable political emigration of relatively more prosperous industrial and white-collar workers. Nearly 650 thousand emigrated between 1890 and 1913—around 14% of the 1890 population. Although the compositional effect of the political emigration of the middle class should increase inequality (as the income distribution becomes more polarized), the overall effect is uncertain sine the price effects can operate in both directions.

The biggest wave of interwar migration took place during the 1920s and was limited to impoverished farmers and agricultural workers. The Polish Statistical Yearbook (1939) and Zubrzycki (1953) report that between 1919 and 1930, 1.3 million people permanently emigrated from Poland (5.3% of the 1921 population), whereas in the next decade the number fell to 300 thousand (less than 1% of the 1931 population). The global economic crisis of the early 1930s disincentivised economic migration, and between 1931 and 1935 more people immigrated to than emigrated from Poland (i.e. the net migration was + 3 thousand).

The negative selection of emigrants during the 1920s and the fall of volume after 1930 could possibly contribute to the growing top income shares throughout the interwar period. If the migration patterns were the primary force, we should observe a structural rise in the level of inequality after 1930. However, during the emigration wave, the rise of the top 1% income share 1924–1930 was bigger (4.1 pp) than in the subsequent period 1931–1935 (2.5 pp), when the emigration essentially stopped (see Fig. 1).

Similarly, as in the pre-WW1 Austrian partition, the stronger rise in inequality during the emigration wave could be related to the changes in the price of land. The emigrants were mainly recruited from impoverished farmers, possibly leading to a fall in land prices and a growing land inequality. Contrary to this, Mieszczankowski (1960) shows a growing parcellation of land ownership structure throughout the interwar period.

There were two waves of emigration during communism. First, as a result of the rise of anti-Semitism at the end of the 1960s, a big portion of the Polish-Jewish intelligentsia left or was expelled from the country. It was politically and culturally a very significant loss, yet numerically the outflow was too small (20–30 thousand) to visibly influence the distribution of income at that time (Wolak, 2004). Also, we would expect that the administrative, cultural and academic positions vacated by the Jews were quickly filled by the Poles, which would prevent any significant changes in the top income shares.

During the 1980s, more than 1 million people (3% of the 1980 population) emigrated, as a result of the major economic and political crisis (Burrell, 2013; Krywult-Albanska 2011; Pleskot, 2015). In terms of demographic structure, the emigrants were mostly young, well-educated and from urban centres, leading to a significant “brain drain”. Notably, 14% of people from medical and IT professions left the country (Okólski, 1994). While it is hard to quantify the impact of this outflow on inequality, the composition effect of the hollowing out of the middle- and upper-middle strata is, in general, to increase economic disparities. On the other hand, the price effects were likely to be muted because of centralized wage-setting. Despite these, however, during the concerned period we observe stable and low levels of inequality.

The most recent wave of migration took place after Poland joined the European Union in 2004. Approximately 1.5 million left the country (4% of the 2004 population), settling mostly in the UK, Germany and the Netherlands (GUS 2018). Dustmann at el. (2015) show that medium- and high-skill workers had relatively largest outmigration rates, yet this migration had only a small positive effect on (non-emigrant) wages for these groups and an insignificant effect on (non-emigrant) wages of low-skilled workers. The composition effects were ambiguous and the price effect was likely to be negligible. Nevertheless, we believe that the overall effect of migration on inequality was limited, because the wage percentile ratios show relative stability in the considered period and top income shares are mostly driven by the rise of business and capital income. It could be still the case, however, that the migration has influenced the relative price of capital to labour and contributed to the growing capital share (Golin and Lange 2013; Quispe-Agnoli & Zavodny, 2002). Investigating this channel is a promising avenue for future research.

In the last decade, Poland has experienced a massive economic immigration from Ukraine, which intensified after the Ukrainian Revolution of 2014. In 2013, 218 thousand (seasonal and permanent) work permits were issued to Ukrainian citizens; in 2017, the number rose to 1.7 million (Jaroszewicz, 2018). Most of the immigrants were employed in low-skilled jobs, possibly contributing to a rise in inequality (Brunarska et al., 2016). Our series stop in 2015, so we do not capture these recent trends.

## Footnotes

1 Our work is a part of the broader project of incorporating distributional statistics in the national accounts (Alvaredo et al., 2016; Garbinti et al., 2018; Piketty et al., 2018; Stiglitz et al., 2009).

2 Figure 13 shows the long run evolution of real GDP per capita since the end of the nineteenth century compared to Western Europe.

3 Poland is not a well-defined entity given the frequent border changes, and for each period we look at Poland in its contemporaneous borders. Figure 14 depicts the territorial changes in Poland since the nineteenth century and shows locations of the main regions discussed throughout the paper.

4 Using cross-regional data from 1927, in Appendix 1 we also point to the persistent legacy of the Partitions’ institutional and legal systems for the level of inequality in interwar Poland.

5 Despite the specific dimensions of inequality during communism, in Online Appendix OA1.6 we argue that money incomes were the most important welfare dimension in socialist Poland.

6 Our work thus sheds a new light on the distributional impact of the post-communist growth and presents a major contribution to the rich literature on the development of inequality in Poland after the fall of communism (Atkinson & Micklewright, 1992; Brzezinski et al., 2013; Keane & Prasad, 2002; Mitra & Yemtsov, 2006; Rutkowski, 1996; Szulc, 2000, etc.).

7 Kuznets (1955) initially proposed a mechanism of the sectoral shift of the population from the traditional agricultural sector to the modern (urban/industrial) sector to explain the development of inequality during the development process (both its rise and decline, e.g. see formalization in Anand & Kanbur, 1993). See Appendix 1 for more details.

8 An equivalent effect is generated by the capital-biased technical change, in which case capital-owners initially reap the productivity gains at the technological frontier (or both, for example, entrepreneurs combine high skills and capital; see Krussel et al., 2000). However, consistent with the Kuznets’ process, inequality may decline eventually, for example, the growth ‘trickles down’ as technology also creates new demand for labor (Acemoglu & Restrepo, 2018).

9 Moreover, Piketty (2014) has recently offered a more sombre view, according to which unchecked economic forces of capital accumulation would adversely affect inequality (notably through high wealth inequality, with well-known *r* > *g* relationship).

10 We provide suggestive evidence that population movements throughout the twentieth and twenty-first centuries are unlikely to be a major factor affecting the evolution of inequality in Poland (see Appendix 3).

11 Factors such as steeply progressive taxation of income and inheritance, the rise of the welfare state and government transfers, strong antitrust regulation and industrial de-concentration, or partial nationalisations, among others, played a decisive role in reducing inequality in Western countries. Correspondingly, a reversal of these factors played the main role in aggravating inequality since the 1980s (Atkinson, 2015; Piketty, 2014). In former communist countries, these policy shifts were more extreme: first by bringing to an end private property and compressing income inequality to an extremely low level; later by being a defining feature of the transition to a market economy.

12 The empirical literature frequently analysed this relationship by looking at the reduced-form approach between inequality and subsequent growth in the cross-country regression analysis (Barro, 2000; Deininger & Squire, 1996; Forbes, 2000; Perotti, 1996, etc.). However, the evidence remains inconclusive (see Banerjee & Duflo, 2003; Kanbur, 2000 for an overview; also Voitchovsky, 2005). It has become increasingly recognized that the focus on the pure reduced form is inadequate and instead it is more promising to take the structural approach, which focuses on specific testable channels through which inequality affects growth.

13 Alternatively, equality might be beneficial for industrialization through higher domestic demand (Murphy et al., 1989; Rosenstein-Rodan, 1943).

14 Instead, interpersonal inequality was dramatically reduced (see Sect. 4.3). Note, however, that the functional (inequality among factor shares in the total income) and personal distribution tended to coincide in societies characterized by a sharp income-source distinction among socio-economic classes, for example, as in the UK in the 18th/19th century. Once income-source heterogeneity underlies different saving behavior, only capitalists save, and their savings drive aggregate savings and growth (Bertola, 2000; Bourguinon, 2005; Lewis, 1954).

15 However, see Sect. 4.4. for qualifications to the mentioned features of the communist development for the growth-inequality link.

16 Actually, faced with low domestic capital stock, Poland and other ex-communist countries aimed to stimulate domestic savings and attract FDI by reducing tax (PIT and CIT) rates. Furthermore, the reduction in tax rates (which could have improved investment incentives and lower distortions) was possible due to lower initial inequality and a consequent lower pressure for redistribution (Alesina & Rodrik, 1994; Persson & Tabellini, 1994).

17 On the other hand, positive effects on profit incentives from privatization could be offset by adverse effects of higher inequality through rent-seeking and corruption amid a weak legislative and institutional framework (Stiglitz, 2012; see Stiglitz, 2002 specifically for the Russian transition). Similarly, the positive effects from improved work incentives may be offset if fairness considerations adversely affect effort at the lower end of the distribution (e.g. see Akerlof and Yellen, 1990). We actually find relatively modest increase in dispersion of the lower half of the earning distribution in Poland after communism (see Fig. 17). For example, a relatively higher minimum wage (Fig. 19) could be seen as ensuring ‘fair’ remuneration at the bottom.

18 We have checked the representativeness of top wages in the enterprise survey by comparing it with the statistics of the ‘equalizing tax’ (*podatek wyrównawczy*), which we found in the Archives of Modern Records (*Archiwum Akt Nowych*) in Warsaw (subject to the tax were individuals earning 3.5 times the average wage in the 1970s, 2 times the average wage in the 1980s). The comparison suggests a strong consistency between sources (see Online Appendix OA.1.6).

19 The rationale behind this assumption is that since the private capital income played a marginal role in the Polish socialist economy, top earnings provide a reasonably good approximation of top incomes.

20 The official HBS reports during the period of socialist Poland give separate presentations for four household types and do not provide distributional data for all households.

21 More precisely, the upgrade factor between survey and fiscal income is 1 for P85 and rises linearly to the observed factor in the tax and survey data. We also apply linear correction factors between empirical thresholds from the tax tabulations.

22 This makes a discontinuity in the series after 1947; however, Atkinson et al. (2011) suggest that the potential bias should not affect the general trend in top income shares.

23 This income concept has been typically labeled as ‘gross income’, which refers to income before all personal deductions and personal income taxes (Atkinson & Piketty, 2007, 2010).

24 Capital income in the form of dividends and interests is taxed separately at source and thus not included in the data.

25 The ‘pass-through’ approach of taxing business income is applied to unincorporated entities, where business income is attributed to the owner who is taxed under the personal income tax.

26 For example, in 1870, out of 16.8 million people, 38% lived in the Russian partition, 36% in the Austrian partition and 26% in the German partition (Landau & Tomaszewski 1985, T. 1.3). The best economic situation was in the Prussian partition, where the authorities carried out many reforms, including the abolition of serfdom. In the Province of Posen and Western Prussia, the capital-intensive agriculture, rather than industry, was the main driver of the economic progress. Silesia, in turn, relied on heavy industry. In the Russian partition, it was also industry that developed the most, with several clusters of textile, metallurgical and mining industries. The delayed abolition of serfdom reforms during the second half of the nineteenth century contributed to the relative backwardness of the agriculture in the Congress Kingdom. However, the worst economic situation was in the Austrian part. Before the end of the nineteenth century, Galicia had not been industrialized, and agriculture was under invested in and parceled (Davies, 2005; Wandycz, 1974). Emigration patterns in the Prussian and Austrian partitions were different, and this could possibly contribute to the divergent trajectories of top income shares in these two regions—see Appendix 3 for a detailed discussion.

27 A promising way forward to investigate this link for the Prussian industrialization is to conceive both directions, as argued by Allen (2009) for the British Industrial revolution. He has argued that technical progress, as a prime mover, was pro-rich and aggravated income distribution (‘Engels’ pause), but the resulting higher inequality allowed the rich to accumulate capital and sustain industrialization.

28 The area was fully incorporated into Poland in 1918, but only the western part remained in the country after WWII.

29 The predominance of employment income in Online Appendix Figure OA6 might suggest that employees in towns, such as in banks or imperial administration, were an important component of top income groups in Galicia. Similarly, top incomes presumably included modest business activities in cities, carried on predominantly by Jews engaged in commerce, handicraft and smaller-scale industry (restrictions on the economic activity of Jews in Austria were largely removed in the 1860s; McCagg, 1989; Wandycz, 1974). Note that we cannot ascertain income sources of particular top groups, as there is no source breakdown for specific tax brackets, but only the income decomposition of those subject to the income tax (approximately the top 2–3 per cent income group).

30 Consistent with the predicted features of the post-Malthusian phase (Galor, 2005; Galor & Weil, 2000), there was in Poland a coexistence of a rapid increase in the population and rising per capita incomes. For example, the annual population growth rate doubled from 0.7% in 1820–1850 to 1.5% in 1870–1890 (Bolt et al., 2018).

31 The Polish-Soviet war of 1919–1920 further disrupted the industrial production and broke the supply chains (Landau & Tomaszewski, 1984).

32 For example, the top marginal rate of the income tax had equaled 5% in pre-WW1 Austria and 4% in Prussia (while in Russia the income tax had never been introduced). In contrast, the top marginal rate in interwar Poland was 40% on unearned income and 25% on earned income. In addition, the cabinet of the Prime Minister Władysław Grabski (1923–1925) launched a whole set of stabilization policies placing the largest burden of their financing on the wealthy.

33 However, there has been surprisingly little research done on the distributional effects of the Polish hyperinflation, and its effects remain ambiguous (see Van Thadden, 1995).

34 For example, through the profit squeeze (excessive wage increase) and increased social spending as debated for Weimar Germany in the “Borchardt controversy” (see Broadberry & Ritschl, 1995). See also Bengtsson et al. (2020) on the role of the shifting power balance for functional inequality.

35 While the economic growth saw an improvement of conditions for all income groups, the rich benefited proportionally more (see Fig. 16; Landau & Tomaszewski, 1985, p. 81). One important external event was the strike of British miners in 1926, leading to a rise in coal prices, which stimulated Polish coal exports.

36 Rough estimates indicate that cartels controlled more than a half of the industrial output in the 1930s (Landau, 1978).

37 The details of the data and methodology can be found in Online Appendix OA1.5.

38 Although, at a first glance, the map looks like a reverse of the previous one, the empirical analysis presented in Appendix 1 shows a strong and positive association between the county-level contribution to the national top 1% income and the within-county top 1% income share. Moreover, this relationship cannot be explained by the potential confounding effect of population size, economy size, urbanization, literacy, land inequality or employment structure.

39 Galor and Moav (2006) have argued that in currently developed countries the rising importance of human capital led to a decline of class politics by aligning the interests of capitalists and workers against those of landowners (see also Galor et al., 2009). To a certain extent, communism acted in less developed countries such as Poland as a ‘functional equivalent’ to the bourgeoisie in developed countries in promoting educational reforms and inducing structural change (from breaking the power of landed elites, to promoting gender equality, etc.; e.g. Milanović, 2019).

40 Actually, the tax data for the immediate post-WW2 years do not point to a dramatic deconcentration *within* the top groups (see Online Appendix Figure OA8). But it is indisputable that a fall in top income concentration occurred eventually as the Communists strengthened their rule in the country. Unsurprisingly, 1947 is the last year for which tax data is available.

41 Social benefits assumed a somewhat greater role in socialist countries than in contemporary capitalist countries, which exerted moderating effects on income inequality. Higher social benefits were generally thought to be at the expense of the smaller wage fund (Atkinson & Micklewright, 1992). This exerted a moderating effect due to a more equal distribution of social benefits (e.g. see Milanović, 1998, Fig. 2.1.).

42 See Online Appendix Figure OA11 for the long-run development of the ‘skill premium’ (also Kalecki, 1964).

43 Flakierski (1991, p. 96) described Solidarity’s income policies as “one of the most egalitarian programmes ever defined in a socialist country”. However, he also points out that the drop in wage ratios could be a result of the poor data quality and collection, inevitable during the economic turmoil during these years. With the looming economic crisis, efficiency concerns resurged, and the communist government directly acted to increase dispersion after 1982.

44 The 1980s was also a period of intensified emigration from Poland. We argue in Appendix 3 that this flow most likely created an upward pressure on inequality.

45 This may be concluded from the relative size of the so-called “surplus”: (1) productivity grew faster than real wages in socialist countries; (2) constantly high shares of gross investment in GDP; (3) a relatively higher ‘share of society’ (most importantly, profits of socialized enterprises, and direct and indirect taxes) versus the ‘share of population’ in the statistics of the primary distribution of the national product (Krejči, 1982). See also Feiwel (1981, T.5).

46 There is a resonance of the ‘classical’ argumentation with the positive effects of inequality to development in the Soviet Industrialization debate, e.g., see Allen (2003). Furthermore, the reduction in wage inequality (see Fig. 5) aimed to allow additional accumulation funds (placing the largest burden on high earnings; Adam, 1984).

47 For example, it is widely accepted that a sharp increase in the investment rate under Gierek in the 1970s was too excessive and accompanied by large inefficiencies and misallocations of resources.

48 Milanović (1998, p. 15) has aptly summarized the main offsetting forces by pointing out that direct subsidies favouring the poor ‘pulled’ inequality down, while non-wage privileges favouring the rich ‘pulled’ inequality up. Unfortunately, it is very difficult to quantify a potential bias caused by various non-monetary dimensions of dispersion (such as *nomenkaltura*’s preferential access to closed shops and services, high-quality housing and holiday homes, etc. (Mathews, 1978); for ingenious attempts at correction see Morrisson, 1984; Atkinson & Micklewright, 1992). Rutkowski, in consequence, has altogether downplayed claims that: “actual inequality was higher than the measured one…is based more on conventional wisdom than on reliable research findings” (1996, p. 91; Atkinson, 2008, p. 323).

49 Similar conclusions can be drawn from the comparison of the Gini coefficient (Online Appendix Figure OA14).

50 Additional evidence of the rising returns to education could be inferred from the rising relative wage earned by more skilled occupations, such as in the financial sector (Online Appendix Figure OA12). Actually, the only dimension of inequality which has not seen a widening of dispersion since the 1990s is gender inequality. In Poland, the earning gap between males and females has been significantly reduced since the fall of communism. While in the 1980s the median wage of women was around 75 per cent of median for men, today the ratio has been around 90 per cent (Fig. 17).

51 In particular, the extensive transfer system aimed to compensate potential ‘losers’ from the market reform, providing on larger scale an access to early retirement to older workers and unemployed, generous unemployment benefits, disability benefits, etc. (Brzeziński et al., 2013, p. 92; Lindert, 2004).

52 Online Appendix Figure OA15 shows that the share of transfers in the total household income from HBS sharply increased after the transition.

53 The other factors might have mattered as well. The abundance of natural resource rents in Russia (their lack in Poland) has plausibly contributed to markedly higher concentration levels in Russia. On the other hand, it has been often argued that an institutional framework, more favourable to the rule of law and to building market institutions, has emerged in Poland as a result of the prospective EU accession (Berglof & Bolton, 2002).

54 The EBRD privatization indicator hardly changes after 2000; therefore, we limit the analysis only to the 1990s.

55 For example, the tax statistics mingle here both the owners of large unincorporated businesses and self-employed individuals mostly deriving income from their human capital.

56 One should bear in mind that since 2004 business income can be taxed separately using a flat tax rate, which was beneficial for high-income individuals and might have induced increased reporting of business income to tax administration. On the one hand, the rise of top business incomes might have been an outcome of lower tax avoidance and evasion (Kopczuk, 2012), on the other, an outcome of shifting of high earnings to business income (e.g. Gordon & Slemrod, 2000). Although the reform probably did have material effects, we believe that it is not the whole story. In the on-going work, we use the registry database on individual-level tax returns in an attempt to empirically disentangle the rise of business income due to the real activity and ‘reporting’ effects (Bukowski et al., 2021).

57 We follow Karabarounis and Neiman (2014) and use capital share of non-financial corporations constructed using OECD data.

58 Another channel could be migration. In particular, abolishing restrictions on migration after Poland joined the EU in 2004 sparked a substantial emigration wave from the country, possibly inducing changes in the composition of population and wages. In Appendix 3, we, nevertheless, provide suggestive evidence that migration was not of primary importance for the evolution of inequality after 2004.

59 The economies in Central Europe are especially sensitive to economic developments in Germany, which is their largest trading partner and a direct investor (Germany is by far the most important of Poland’s trading partners). For instance, an increasing participation of Poland in German-led global value chains (GVC) might have also contributed to the synchronization of top shares in two countries (Online Appendix Figure OA17). This is visible in Fig. 7b, which shows similar predicted non-causal changes in income shares due to the actual changes in capital share and export share in Poland and Germany (the methodology is described in Sect. 5.2). Nevertheless, the co-movement of Polish and German top shares might weaken in the future, if the importance of the domestic market in Poland continues to grow. A decoupling of GDP growth rates between these two countries is already visible in the period after 2015.

60 We speculate that higher income concentration in Poland than in other former socialist countries in the EU could be linked to relatively higher number of affluent entrepreneurs, plausibly benefiting from the larger domestic market.
