# Supplementary material for: Between communism and capitalism: long-term inequality in Poland, 1892–2015
Source: J Econ Growth (Boston). 2021 Jun 2;26(2):187–239. doi: 10.1007/s10887-021-09190-1 (PMC8550671; doi:10.1007/s10887-021-09190-1)
Supplement: Supplementary file 1 — Supplementary file1 (PDF 1945 kb) [file 10887_2021_9190_MOESM1_ESM.pdf]

Between Communism and Capitalism:
Long-Term Inequality in Poland, 1892-2015

Paweł Bukowski and Filip Novokmet

ONLINE APPENDIX

Online Appendix OA1:
Top Income Shares, 1892-2015; Methodology and Data Sources

Table OA1 provides overview of the main data sources used for the construction of the top income shares for each period of our series. The three main sources are the Ministry of Finance's PIT tabulations (1924-1947, 1992-2015), the earnings surveys (1955-1989) and the statistical yearbooks reporting tax data (1892-1917). The number of available brackets changes through time, however the top brackets capture always at most top 2% of population, and most of the time less than top 1%.

Table OA1: Overview of the data sources

Years	Source	No. of Brackets	Fraction of taxpayers in the top bracket
2009-2015	PIT tabulations	2	1.3% (2009); 2,3% (2015)
1995-2008	PIT tabulations	3	1.1% (1995); 1.2% (2008)
1994	PIT tabulations	3	1.3%
1992-1993	PIT tabulations	7	0.1%>
1988-1989	Earnings Survey	9	1.0%
1985-1987	Earnings Survey	13	0.3%
1983	Earnings Survey	8	1.0%
1970-1982	Earnings Survey	7	0.1%< (1970); 0.4% (1982)
1963-1967	Earnings Survey	4	0.7%
1955-1962	Earnings Survey	6	0.1%< (1955); 0.4% (1962)
1947	PIT tabulations	40	0.1%>
1946	PIT tabulations	43	0.1%>
1935, 1938	Statistical Yearbook	8	0.1%>
1936	PIT tabulations	73	0.1%>
1930-1931	PIT tabulations	14	0.1%>
1925-1929	PIT tabulations	73	0.1%>
1924	PIT tabulations	49	0.1%>
Prussia: 1892-1917	Statistical Yearbook	6	0.1%> (1892); 0.1%> (1910)
Austria: 1898-1912	Statistical Yearbook	8	0.1%>

Note: the fraction in the last column is related to the total control population. Prussia is the Province of Posen and West Prussia; Austria is the Austrian Partition.

OA.1.1. The Prussian Partition 1890-1918

Data. Prussia assumes a special place in the analysis of historical distributional patterns, primarily due to the early introduction of the comprehensive income tax in the nineteenth century, which was accompanied by regular annual publications of the detailed statistics. Most importantly, this coincided with the industrialization and the structural transformation of the country's economy, the emergence of the modern economic growth and the eventual rise of Germany to the global economic pre-eminence.[1] For the same reason, the Prussian income tax data offer invaluable research opportunities to study the long-term evolution of inequality in Poland.

Data for Prussian Poland come from the annual Statistics of income tax assessment (*Statistik der preußischen Einkommensteuer-Veranlagung*). We use available tabulations for provinces (*Provinzen*) and districts (*Regierungbezirke*) to construct top income shares for Prussian provinces with predominantly (or significant) Polish population, which formed after WWI the Second Polish Republic (1918-1939). Top income shares can be constructed for the provinces of Posen and West Prussia. The province of Silesia, however, should be distinguished from the first two provinces, as Germans accounted there for the predominant part of the population in the pre-WW1 period, and only the district Oppeln (*Opole*) joined the interwar Poland (as Upper Silesia). The region itself did not form a part of Polish-Lithuanian Commonwealth (moreover, it became a part of Prussia only after Frederick the Great had taken it from Habsburgs during the so-called Silesian Wars) and it was included in Poland after the Second World War. Parts of the Prussian provinces of Pomerania and East Prussia are today within the Polish borders (the other parts of the former Prussia are in Germany, Russia and Lithuania), but we do not investigate them separately as these were not generally identified as the 'historic Polish lands',[2] and use them in analysis for comparative purposes.

Published tabulations are ranged according to brackets of gross income, giving for each bracket the number of taxpayers and the corresponding tax obligation. Statistics at the level of districts provide almost seventy brackets, which can be aggregated to the provincial level. In addition, there are separate reports for the number of taxpayers in towns and in the countryside at the provincial and the district level (these were ranged by six brackets). However, the sources of income are not available at the bracket level, but only in total for all taxpayers.

Population Control. The tax unit in Prussia was household, defined as the married couple with dependants. The total number of households in provinces is estimated from the Population Census (*Die Volkszählung im deutschen Reich*) and the Statistical Yearbook (*Statistisches Handbuch für den Preussischen Staat*; *Statistisches Jahrbuch für das deutsche Reich*).

[1] The use of the Prussian data has been used for coining path breaking theories in the development economics concerned with the interaction of inequality and economic growth, or the often-termed literature in the Kuznetsian tradition. The Prussian income data actually served as the basis for the Kuznets' inverse-U evolution of inequality during the economic development. They present an unambiguous evidence of the rising inequality during the industrialization phase of the country in the second half of the nineteenth century until the First World War, as well as the ensuing fall afterwards (Prokopovitch 1926; Kuznets 1955; Müller and Geisenberger 1972; Keaelble 1986; Dumke 1991).

[2] For example, Prussians never included them in widely used term of 'our Polish provinces' (Davies 2005).

Income Control. The income control totals for provinces in Prussian Poland have been obtained by estimating the income of those exempt from the income tax ('non-filers') (e.g., Prokopovitch 1926). The statistics provide both the total number of taxpayers (filers) and non-filers for each province and district. With the reported total income of taxpayers, it remains to estimate the total income of non-filers. We assume that non-filers in each province had the same average income as in Prussia on the whole. The figures for Prussia are obtained from Hoffman and Müller (1959, Tab. 35), who estimated them based on Statistische Reichsamt (1932). The latter also provides the income of tax exempt at the provincial level for 1900, 1907 and 1913. The available estimates for these years are very close to those obtained by the above method. Hoffman and Müller (1959) do not cover the 1914-1918 period, so we take the average income of non-filers in Prussia from Dell (2008), who followed the methodology of the former authors.

OA.1.2. The Austrian Partition 1890-1914

Data. Top income shares in Galicia are constructed from income tax statistics for Imperial Austria found in Statistical Yearbooks of Imperial Austria (*Österreichisches Statistisches Handbuch für die im Reichsrathe vertretenen Königreiche und Lände*) as well from Annual Report of Ministry of Finance (*Mitteilungen des K. K. Finanzministeriums*). After the income tax was introduced in 1898, the fiscal administration was publishing tabulations of income taxpayers in each province of Cisleithania. Income definition was quite broad allowing very few exemptions. It defined income from following sources: land, buildings, business and self-employment, capital and other sources. Capital gains were not taxed. Tax unit was a family with the total income of family members ascribed to the head of a family.

Population Control. The tax unit in Imperial Austria was household, defined as the married couple with children. The total number of households in Galicia is estimated as the number of adults (above 18 years of age) minus the number of married females. The data come from the Austro-Hungarian censuses of 1890, 1900 and 1910 *(Die Ergebnisse der Volkszählung in den im Reichsrathe vertretenen Königreichen und Ländern).*

Income Control. The control total for income for Galicia during the Habsburg era is derived as follows. We take as our starting point Schulze's (2007) estimates of regional GDP in Austria-Hungary. Schulze provides estimates for 1870, 1880, 1890, 1900 and 1910, expressed in 1990 Geary-Khamis international dollars. To convert estimates for Galicia into current Austro-Hungarian crowns, we take the following steps. First, we convert these estimates to 1913 crowns by applying the exchange rate used by Schulze (namely 3.36 GK dollars per crown; see Schulze 1997, p. 14). To obtain GDP for the years between 1890, 1900 and 1910, we apply real growth rates of GDP for Galicia taken from Ciccareli and Missiaia (2014). Next, nominal values were obtained by using regional living cost indices in Austria-Hungary estimated by Cvrcek (2013). Finally, we take 60 per cent of nominal GDP as our total control income.

OA.1.3. The Russian Partition

The modern income tax did not exist in the Russian Empire Consequently, there is no comprehensive information on income distribution for the Russian partition until the unification of the country in 1918. The Tsarist tax administration provided estimates of the income distribution for 1905 in preparation for the eventual introduction of an income tax, which, as said, was never introduced (see Novokmet et al 2018 for

the application). However, these tabulations do not provide regional perspective (Gregory 1982, Lindert and Nafziger 2012).

Załęski (1901) in his statistical description of the Congress Kingdom attempts to estimate the distribution of non-wage income using auxiliary data on the distribution of land (for farmers) and firm size (for entrepreneurs). He defines three ad-hoc income groups based on the size of arable land and the number of employed workers (i.e., bigger firms and landholdings are associated with higher income). In 1901, over 31% of non-wage earners and their families were classified as "poor", 67% as "middle", and 2% as "rich". Importantly, Załęski conducts a similar exercise for the whole Prussia and shows that 28% of non-wage earners and their families were "poor", 69% were "middle" and 3% were "rich". Keeping in mind that the top income shares for the Prussian partition were lower than in the whole Prussia (see Section 4.1), these back-of-envelope estimates suggest that the level of inequality in the Prussian ad Russian parts of Poland were relatively similar.

One can obtain additional insight about inequalities in the Russian partition from the regional data from 1927 discussed in Section 3.2. We construct aggregated top 1% income share for each former partition from the county-level data (we exclude Silesia from the analysis). The former Russian partition shows the lowest level of the top 1% income share (12.7%), placing it below the former Prussian (14.8%) and the Austrian (15.5%) partitions. It should be also noted that the top income shares in the Russian partition, compared to the other parts, possibly experienced a stronger decline after 1918. The social legislation introduced in the early 1920 disproportionally benefited industrial workers, who were concentrated in the industrial clusters located of the former Russian partition (Sztrum de Sztrem 1922; Derengowski 1930; Wolf 2007).

In terms of land inequality, the Gini of land ownership (we draw the data from the tax report in 1927) is also the lowest in the former Russian partition (0.5), below the former Austrian (0.54) and especially Prussian (0.68) partitions. We interpret these numbers as the lower bounds of the levels of land inequality during the 19th century, since the concentration of land ownership across all partitions declined since the beginning of the 20th century until the 1920s (Mieszczankowski 1960).

The within-partition, cross-county dispersion in top 1% income share is visibly higher in the former Russian partition. The eastern parts stood out in term of high-top income shares, which could be linked with a traditional presence of land magnates, a social class of big and wealthy landowners of noble origin. The western counties of the former Russian partition (the central regions of the Interwar Poland), in turn, had relatively lower inequalities, but high mean income (see Figure 6).

OA.1.4. Interwar Poland 1918-1939

Data. The tax data come from the official publications of interwar Ministry of Finance, the Central Statistical Office of Poland, as well as Ministry's archives in *Archiwum Akt Nowych* in Warsaw. For more details see Table OA2 below:

Table OA2: Data sources for the period 1918-1939

Source Name:	Years available:	Publisher and Comments
Rocznik Ministerstwa Skarbu 1928 (Yearbook of the Ministry of Finance 1928)	1924, 1925, 1926	The Ministry of Treasure / The Ministry of Finance
Rocznik Ministerstwa Skarbu 1927-1930 (Yearbook of the Ministry of Finance 1927-1930)	1925,1926,1927,1928	The Ministry of Treasure / The Ministry of Finance
Statystyka Podatków Bezpośrednich, Opłat Stemplowych i Danin Pośrednich 1931	1925,1926,1927,1928	The Ministry of Treasure / The Ministry of Finance
Statystyka Wymiaru Państwowego Podatku Dochodowego za Rok Podatkowy 1927	1927	The Central Statistical Office of Poland
Witold Berhnard, "Obciążenie Państwowemi Podatkami Bezpośredniemi", Kwartalnik Statystyczny VIII (4) 1931, p.901-919	1929	The Central Statistical Office of Poland
Statystyka Skarbowa 1933	1929, 1930	The Central Statistical Office of Poland; the data do not separate legal and psychical persons
Statystyka Wymiaru Państwowego Podatku Dochodowego za Rok Podatkowy 1936	1936	The Central Statistical Office of Poland
Ministry's archives in Archiwum Akt Nowych in Warsaw	1929, 1936, 1938	Incomplete, only earnings.

The tax code defined two types of income: unearned (*fundowany*) and earned (*niefundowany*). The unearned category included income earned by either legal or psychical person, whose economic activity is independent, for instance, capitalists, entrepreneurs, self-employed, artisans, farmers or petty bourgeoisie. A broad range of activities was taxed this way, including income from agriculture, forestry, land and real estate rents, business activities, capital income (e.g., interests, dividends), royalties. Non-monetary income, such as natural consumption or imputed rents of owner-occupiers, was not subject to taxation. Earned income was attributed to employees or retired physical persons. Importantly, state workers and state pensioners do not appear in the tax statistics, even though they were liable to PIT.

For psychical persons only, annual income above 1500zł for unearned income and 2500zł for earned income had to be reported. These two types of incomes were subject to different tax schemes, and the tax statistics provide separate tabulations for each type. However, this implies that psychical persons who earned both unearned and earned incomes were reported twice. Similarly, a person was reported multiple times if her/his earned income came from more than one employer in different tax catchment areas. To our best knowledge, it is impossible to separate these individuals. Therefore, we assume 'Ricardian' system of distinct classes, that is, that of zero overlap between unearned and earned taxpayers at the top

and we do not account for the multiple employer problem (we believe that the latter should not be an important issue).

The tax code used two definitions of the tax unit: household and individual. An individual reported income from self-employment, wages or pensions. All other types of income were reported at the household level. This creates a problem to arrive at a clear definition of the tax unit for unearned income, since it could be either household or individual. In the case of earned income, it was always individual.[3]

PIT covered a very broad range of economic activities, excluding only incomes from inheritance, capital gains, income of non-profit oriented entities, lotteries and other minor sources. A taxpayer could deduct from the total income: paid interests on loans, rents and permanent financial obligations originating from the legal requirements, social security (up to 300zł), insurance benefits (up to 300zł per individual or 600zł per household) and other taxes. In the case of unearned business income*,* the actual income was reported by taxpayers with accounting books. For others, the administrations imputed income based on a set of payer's characteristics.

Tabulations are ranged according to income before deductions and taxes.[4] For each income bracket there is information on the number of taxpayers and the total tax paid. All incomes within a bracket paid a fixed amount of tax. We estimate the total income in each bracket by assuming Pareto distribution (see below). The relatively high exemption levels in this period entailed that only a small proportion of the population was subject to the personal income tax and consequently appeared in the tax statistics. Thus, when analysing the interwar tax statistics, we effectively deal with the top tail of the income distribution. It has been found that this distributional form conforms quite well to the top of the income distribution (e.g. see Atkinson and Piketty 2007). However, since brackets' width is quite narrow, our estimates do not depend on particular distributional assumption. As a result, the total bracket income is robust to different distributional assumptions (for example, the resulting income is practically the same if, rather than assuming the Pareto form, we assume that incomes within income brackets are uniformly distributed). This is further corroborated by years when both the number of taxpayers and their corresponding income in brackets is reported in published tabulations (e.g. in 1946/7).

For the years 1925 and 1926, the tax published tax statistics do not separate physical and legal persons reporting unearned income. We estimate the number of physical persons in each bracket of unearned tax schedule in 1925 and 1926 by taking the proportion of physical persons in all 'unearned taxpayers' observed in 1927 (note that ranges of reported income classes were unchanged throughout the years). In general, the proportion of legal persons in total unearned taxpayers is very small, corresponding to less than 1% of all unearned taxpayers (0.8% in 1927, 0.7% in 1928 and 1929), but these are dominantly concentrated at the very top of the income distribution. However, the proportion of legal persons is quite stable throughout the

[3] Wiśniewski (1934) unfortunately does not discuss this issue in detail. Using the census data, for unearned income, he estimated the control population assuming that the tax unit are: agriculture holdings, for-profit entrepreneurs, self-employed, petty bourgeoisie. For earned income he distinguished between agricultural workers and other workers.

[4] One potential issue is that the tabulations contain only taxpayers that paid personal income tax. Even if an individual's income was above the minimum filing threshold, after deductions her income could fall below the threshold and did not appear in the statistics. However, since we look at fractiles whose thresholds are much higher than the minimum filing threshold, this does not lead to underestimation of top income shares.

years. For example, when the proportion of physical persons in the total taxpayers in 1926 is taken to correspond that observed in 1927, the top 1 per cent (the top 0.1 per cent) share is 11.68% (3.56%). When the proportion from 1928 is taken instead, the top share is 11.78% (3.65%). Even when we apply the proportion documented a decade later, in 1936, our estimates are not significantly affected (11.57% (3.49%)). These margins of error seem reasonable enough to use our estimates for 1925 and 1926 without raising too much unease.

The same approach is taken for 1930 and 1935, for which equally the statistics on unearned income does not distinguish between personal and legal persons. We take the proportions of physical persons in all unearned taxpayers documented in 1929 and 1936, respectively. Unfortunately for these years, the statistics on earned income is also lacking. However, as unearned income accounts for the predominant part of income at the very top (for example, it made almost 90% of income for the top 0.1 per cent and above; e.g. see Figure OA7) and rises with income rank, we provide the estimate for the top 0.1 per cent and the groups above by simply taking the number of taxpayers reporting earned income in 1929 and 1936 in the corresponding top brackets. In addition, as top earnings exhibited certain rigidity during the depression, it is reasonable to assume that the 'crisis years'—1930 and 1935—displayed a similar earnings distribution at the very top as observed for the immediate neighbouring years for which the statistics are available.

Population Control. As mentioned above, the tax unit in interwar Poland was both a household and an individual depending on the income source obtained. Namely, someone obtaining employment income was individually taxed, while for other sources incomes of all family members were combined and attributed to the 'head of family'. We take as our population control a hybrid construct defined as the total number of adults minus the number of married women not employed or self-employed. Our definition thus treats working females as separate tax units, but note that most of them were actually not married (according to 1931 census, less than 15% of employed females outside agriculture were married (Maly Rocznik 1939, p. 260, Tab. 5)), and therefore the total reference roughly corresponds to the total number of married couples plus singles.

The number of adults is taken from population censuses (and annual figures from the statistics on the Movement of the Population), while the number of non-working females is equally found in censuses and linearly interpolated for in-between years.

Income Control. To arrive at the total control for income, we take the estimate of Kalecki and Landau (1934) for 1929 as our starting point. This estimate has remained the main reference point for all subsequent estimation of national income in interwar Poland up to present day. Kalecki and Landau's (K&L) estimate is gross of depreciation, roughly corresponding to GDP and based on the expenditure approach. K&L extended their calculations only for 1933 (Kalecki and Landau 1935), so we have relied on studies of Klarner (1937) and Petyniak-Sanecki (1939) for other years in the 1929-1936 period for which the tax data is available. The latter authors followed closely the methodological approach used by K&L (Landau 1976, pp. 110-1).

However, no subsequent study focused on the years before 1929. We adopted the following approach to estimate total income in 1927 and 1928. K&L provide indices of the real development of the national income for the period 1927-1934 (1935, Tab. 116).[5] We take K&L's GDP for 1929 and apply the

[5] The methodology was developed within the Institute for the Study of Business Cycles and Price. These series are not based on comprehensive estimates of consumption and investment as for the 1929 and 1933 (see Kalecki and Landau 1935).

corresponding growth rates to obtain real GDP figures in 1927 and 1928. We checked the K&L indices by comparing them with the real GDP growth rates in Maddison (2001) (available for 1929 to 1938; from Laski (1956)) and find quite close development. This should come as no surprise since Maddison takes the estimates of the Institute of Economic Sciences of the Polish Academy of Sciences, which are based on the work of K&L. Finally, to obtain the nominal level, we use the average of the wholesale price index and the retail price index (*Maly Rocznik Statystyczny* for 1933, Tab. 1, p. 93).

The next step in using the 'top-bottom' approach for the total income control consists in subtracting from GDP items not included in personal income such as the consumption of fixed capital, public sector income, retained earnings of corporate sector, or non-taxable personal income. Due to the lack of detailed historical national accounts, especially with respect to the income method, the usual practice for estimating personal income has been to assume some fixed fraction of GDP (Atkinson and Piketty 2007, 2010). Wisniewski (1934) in the study on income distribution in Poland in 1929 estimated the total taxable income as equalling 82 per cent of the K&L national aggregate. However, Wisniewski's total income does not only add the income below the minimum exemption level (that is, the income of non-filers) to the total reported income of filers, but he 'corrects' the tax data through the whole distribution by using alternative sources (such as the distribution of agricultural holdings from the land tax in order to account for the assumed misreporting of income derived from the land). Consequently, we take a smaller proportion of GDP than Wisniewski did, namely 75 per cent of GDP.

For 1925 and 1926 we exploit the available estimates of national income. These are net of depreciation, so we assumed that the total control for income equals 80 per cent of national income. Following Kazimerz Secomski, the consumption of fixed capital is taken as 5 per cent of GDP (Landau 1976, p. 110). Landau (1976) reports dozens of national income estimates of various authors for the 1923-5 period. The range of estimates is quite large. Those that explicitly refer to 1925[6] are in a range between 15 and 20 million zl (in 1927 parity), and as a middle ground, we take 17,5 mil zloty as an estimate of national income. The year 1926 was the last year of post-inflation depression (Landau and Tomaszewski 1985) and we assume no real growth between the years.

One should note that in 1927 there was a change in parity of zloty to gold franc, with 1 zloty of 1924 worth 1.72 zloty of 1927. However, the tax statistics for 1924-6 was published from 1927 onwards, and taxpayers in the mentioned years are ranged according to the brackets denominated according to the new parity. Consequently, when estimating the total control income for 1924-6 one needs to convert available estimates of national income from 1924 parity to 1927 parity.

Estimation of income in tax brackets. For most of the years in the interwar period, only the number of taxpayers in specific brackets of gross income is reported without providing their corresponding income. We estimate income in each bracket by assuming that top incomes follow Pareto distribution.

Pareto cumulative distribution function $F(y)$ for income y is:

$$1 - F(y) = (k/y)^{\alpha}$$

[6] As stated by Landau (1976, p. 105): „In many cases, it is also difficult to determine precisely for which year the estimate was made. We know that they relate to the years 1923-1925."

where $1 - F(y)$ is the proportion of tax units with income above y. Parameters k and a are given; k presents the minimum income to which the Pareto distribution is applicable ($k > 0$), and a presents the slope of distribution ($a > 1$) (Cowell 2011).

In order to estimate amounts in bracket (s, t), it is assumed that income in each bracket is distributed according to Pareto law. Let p present the proportion of tax units above s and q the proportion of tax units above t, then:

$$p = (k/s)^{\alpha}$$
$$q = (k/t)^{\alpha}$$

From these equations, we obtain parameters a and k:

$$a = log\,[(p/q)]/log\,[t/s]$$
$$k = sp^{(\frac{1}{a})}$$

We estimate a and k for each bracket. Finally, the income in bracket (s, t) is estimated as

$$Y = N \int_s^t ydF(y)$$

where N is the total number of tax units. However, this method cannot be applied to the top bracket. We assume that Pareto coefficient in the top bracket to be the same as the bracket immediately below it.

OA.1.5. The county-level inequality in Interwar Poland

Data. The 1927 Income Tax Statistics published by Ministry of Finance provides the detailed tabulations of earned and unearned income for each tax office in Interwar Poland. In rural areas, there was usually one office per county, whereas in urban areas there were usually more than one, in which case we aggregate the data to the county level. In three cases, Gniezno, Inowroclaw and Lublin, the tax office catchment areas were larger than the corresponding county and covered the rural and urban counties. We merge these counties to match the tax office.

Since the data on population and income controls is from the 1931 census, we match the 1927 tax offices with the 1931 counties. It was straightforward for the majority of cases, except those counties where borders changed, or which were liquidated between 1927 and 1931. In these cases, we assign a tax office to a 1931 county, which received the largest portion of a 1927 county. We drop Konstantynów and Królewska Huta counties, as it was impossible to determine a corresponding 1931 county.

Similarly, as in the aggregate tax tables, the highest bracket for unearned income is open. To determine income in the top open bracket, we assume that in each county two top brackets follow the Pareto distribution. Finally, to obtain the total amount of income, we assume that unearned and earned income taxpayers are different individuals and merge their number for each bracket. The width of earned income brackets is usually narrower than of unearned income, so it is straightforward to adjust them when combining the two schedules.

Population Control. Similarly, as for the country-level analysis, we take as our population control the total number of adults minus the number of married women not employed or self-employed. The county-level data comes from the 1931 census.

Income Control. We construct control income for each county consistently the reported taxable income from the 1927 Income Tax Statistics. We separately estimate the earned income of agriculture and non-agriculture workers; the exempted unearned income of independent in agriculture and non-agriculture activities.

For earned income of non-agriculture workers, the data on the voivodeship-level total compensation of industrial workers in nine industries (mineral, metal, electro technical, chemical, textile, paper, tannery, wood and food) come from the 1931 Industry Statistics (*Statystyka Przemysłowa 1931*). The county-level data on the number of workers in fifteen industries and non-manufacturing sectors come from the 1931 census of population. To obtain the total earned non-agriculture income for each county, we calculate the average compensation for each voivodship-industry cell and multiply it by the county-level number of workers in the corresponding industry (*Drugi Powszechny Spis Ludności z Dn. 9.XII 1931 r.*). For the industries not covered by the 1931 Industry Statistics, we use the average voivodeship-level compensation; for domestic servants, we use one-third of the average; for public administration workers we use 2/3 of the average, and for the remaining workers we assume 1/2 of the average. In other words, we assume that all workers in voivodeship-industry cells earn the same average compensation. To obtain the total earned non-agriculture income we add the estimated earned income of industry workers, domestic servants, public administration workers and others. In addition, we increase the total amount by 50% to match the country total.

To obtain earned income of agriculture workers, we calculate the average income of agriculture workers and the average income of agriculture 'white collar' workers (*dozorca*) for each voivodeship using use the data from Gerlicz (1929). As the original data is in the quintals of rye, we use *The Statistics of Prices 1929* (*Statystyka Cen, 1927-37*) to translate these amounts into the Polish Zloty. Next, for each county, we multiply the number of agricultural manual workers by the voivodeship average and the number of agriculture 'white collar' workers by the voivodeship average for 'white collar' occupations. To obtain the total earned income of agriculture workers we sum up the total income of ordinary and 'white collar' workers.

Next, we estimate exempted unearned non-agriculture income. The 1931 census provides the county-level number of non-agriculture independents, which we multiply by one-third of the average unearned taxed income. Unfortunately, to the best of our knowledge, there is no available separate data on the income of independents in non-agricultural sectors.

Finally, we calculate exempted unearned agriculture income. First, based on the estimates from the Puławy Institute and Wisniewski (1934) we assume that all landholdings smaller than 5ha did not pay the income tax. The total number of these landholdings is taken from the 1927 land tax. Second, we assume that only in certain counties landholdings between 5-10ha paid the income tax. In particular, we take the number of landholdings from the 5-10ha band in the 1927 land tax, if it is smaller than the difference between the

hypothetical number of exempted agriculture independent[7] and the number of landholdings smaller than 5ha. Otherwise, we use the difference. Finally, we multiply the number of landholdings in each band by 2/3 of the average voivodeship-level agriculture income reported by the Puławy Institute. We take the fraction of the income because the estimates are believed to be upward biased (Wisniewski 1934).

To obtain the total county-level control income, we sum up the earned agriculture income, the earned non-agriculture income, the exempted unearned agriculture income, the exempted unearned non-agriculture income and the unearned taxed income.

OA.1.6. Communist Poland 1945-1989

Data. The design of income tax during the first years after the end of WW2 kept broad contours of the interwar legislation. The major change, however, was to exempt earned and agriculture incomes, and tax only non-agriculture unearned income. In addition, the socialised sector was not a subject of taxation, and the law set a relatively high income threshold. Consequently, with continuous government's attempts to limit private entrepreneurship, the income tax *de facto* lost its economic importance.

Tabulations of taxpayers obtaining unearned income are available for three years in the late 1940s: for 1945, 1946 and 1947. Unfortunately, there are no corresponding tabulations for earnings. But in order to provide an indication of the post-war development of top income shares, we combine the income tax statistics on unearned income for 1947 with the earnings data from employer survey in 1949. Obviously, the critical assumption has been that earnings distribution remained stable between 1947 and 1949.

Earnings survey provides tabulations of employees in industry and construction, respectively, ranged according to monthly earnings. Separate reports are given for manual and white-collar workers, ranged separately for technicians and office workers. We merge respective tabulations according to worker's qualification, and then of all workers in industry and construction. The resulting joint distribution accounts for roughly 70 per cent of employees covered by social insurance in firms with more than 5 employees (exclusive of agriculture). We assume that the remaining 30 per cent of employees (e.g., in telecommunication, wholesale or retail trade, accommodation) is distributed in the same manner as those in (combined) industry and construction. On the other hand, it is assumed that employees in firms with less than 5 employees, or employees in agriculture as well as in those not covered by social insurance, do not end up in higher earnings brackets, and thus do not make up top income groups. We adjust earnings bands in 1949 to the price level of 1947 by using the available retail price index.[8] Annual earnings were obtained by multiplying bracket middle point by twelve. For the earnings in the open top bracket, we assumed that two top brackets follow Pareto distribution. Finally, as in the interwar period, we assumed no overlap between individuals obtaining unearned income and earnings.

In order to construct top income estimates for the 1956-1990 period (Figure 2) we use enterprise wage surveys, which covered employees in the socialized sector (for sources and details see the Online Appendix

[7] The number of hypothetical exempted agriculture independents is calculated using the 1937 tax data, which reported the number of taxpayers and income for the total unearned sector and for unearned agriculture. We relate two ratios, the agriculture taxpayers/agriculture income and total taxpayers/total income. Next, we apply this relationship to the 1927 tax data to obtain the number of agriculture taxpayers (which is not reported). Finally, we subtract this value from the total number of smallholdings from the 1927 land tax.

[8] However, as we are aware, this is available only for Warsaw.

OA.3). The statistics covered all workers in the socialised sector, which accounted for the greatest part of the labour force (Atkinson and Micklewright 1992, Atkinson 2008). Published tabulations range workers in the large number of brackets according to the size of their gross earnings. Atkinson and Micklewright (1992, p. 257) point out that the definition of gross earnings "include basic pay, overtime, compensation for hazardous work conditions, additional payments related to job tenure or the holding of a managerial position, profit shares, bonuses and premia". We assume that only wage earners constituted top income groups in this period, since unearned income was to the greatest extent expropriated by the state after a thorough nationalisation wave and the land reform in the late 1940s, coupled with the currency reform in 1950. The remaining non-wage private income was largely concentrated in the small-scale agriculture, characterised by the low productivity and the small earning potential, and thus plausibly did not contribute to top incomes. We impute non-wage income as follows. Income of self-employed in the agriculture and pensions are wholly attributed to the bottom bracket. As said, the bulk of non-wage private income was concentrated in the small-scale agriculture, characterized by the small earning potential (as it was heavily constrained by the small maximum holding size and the maximum number of employees allowed). Other self-employment income and other cash transfers are uniformly distributed among all adults.

In order to check the representativeness of top wages in the enterprise survey, we compare it with the statistics of the 'equalizing tax' (*podatek wyrównawczy*), which we found in the Archives of Modern Records (*Archiwum Akt Nowych*) in Warsaw (subject to the tax were individuals earning 3.5-times the average wage in the 1970s, 2-times the average wage in the 1980s). Namely, the wage survey did not include armed forces and police or senior government officials (Atkinson and Micklewright 1992), while the latter included. The comparison suggests a strong consistency between sources, resulting in practically equivalent top income shares of top 0.1% group (the inverted Pareto coefficient of P99.9-100 is around 1.3 according to both sources). A corresponding picture is obtained from the personal income tax data in socialist Yugoslavia, exploited in Novokmet (2017).

Population Control. For the population control in 1947, we used the same definition as for the interwar period. The population unit in the 1956-1990 period is an individual. Note that this implies that our control total for the population includes, in addition to those employed in the socialised sector, importantly self-employed (largely in the private agriculture) and pensioners. Note accordingly that high-earning individuals from the wage statistics account for the top 5% of the total adult population (rather than of all workers only). The data is taken from the population censuses and the Demographic Yearbook of Poland (*Rocznik Demograficzny*).

Income Control. To arrive at the total income for 1947, we use the official estimate for the national income (*Rocznik Statystyczny* 1949, p. 27, Tab. 1). This figure, however, refers to the Marxist concept of national income,[9] corresponding to the net material product (thus exclusive of services, or 'non-productive' activities such as housing, education, administration, etc.). We increase this figure by 15 percent to obtain the estimate of national income according to SNA, as this proportion has been often found to account for services (according to GUS 1949), services in the interwar period accounted for 17 percent of national income). We take 65 per cent of this adjusted figure to correspond to the total income control. This is somewhat lower proportion than used in the interwar period, because the communist accession to power resulted in the increase of the so-called 'social income' (and thus a fall in personal income) in national income, especially through a rise in retained profits of nationalised enterprises (a fall in the wage fund) needed for investment.

[9] Accounting system in communist countries was the Material Product System (MPS).

The total control income for 1956-1990 is derived from the national accounts reported in the Statistical Yearbooks. The total income is the sum of wages and salaries, farmers' income (money and in kind), self-employment income (*szacunek dochodow netto ludnosci z prowadzenia dzialnosci gospodarczej*), pensions (*emerytury i renty*) and money income from subsidiary economic activities.

O.1.6.1. The shape of the upper tail before and after WW2

Tabulations for 'unearned' income were published in first years after the Second World War (1945, 1946 and 1947). However, both physical and legal persons are grouped, without providing separate presentations as before WW2. Yet, we believe that the picture corresponds mostly to physical persons as only private entities were taxed and most legal big (joint-stock) companies in Poland were nationalised immediately after the war.

Figure OA8 compares the shape of the upper tail of the distribution of unearned income before and after the Second World War by looking at the ratio of average income above the given threshold to that threshold. This concentration measure is useful for comparative purposes as it does not depend on changing income levels through time. Note that higher ratio implies higher concentration at the top, while it is constant if the distribution assumes the Paretian form (inverted Pareto coefficient b). The figure presents roughly 200 thousand top taxpayers obtaining unearned income in 1936, 1946 and 1947.[10] The top concentration before the war is quite similar to that observed immediately after the war. It seems that the war and the occupation, as well as the immediate effects of the introduction of communism, did not dramatically affect top concentration patterns. This would suggest that the fall of top income share between 1936 and 1946-7 was driven by the rise of its denominator, not the fall of nominator, in other words, economic conditions of the entire population relatively improved.[11]

O.1.6.2. Interpreting data for the communist period

It is important to acknowledge the limitations of the official data on income distribution during the socialist period. To be more precise, we largely deal with money incomes (plus some important non-monetary income components as farmers' income in kind), which omits important non-monetary dimensions of inequality during this period. In addition, some features of the socialist economies might have entailed that surveys did not get the distribution of money income right (e.g., such as relatively higher informal sector).

First, there is no doubt that money income was not the only factor influencing dispersion in socialist Poland. Although it may be generally said that this was never the case in historical societies (e.g., the importance of inequalities in personal status and basic rights), it needs to be recognized that this issue was particularly acute in socialist economies. It is sufficient to stress non-monetary features of socialism, such as shortages

[10] It should be noted that comparison is not perfect, as clearly cumulative frequencies do not correspond to same shares of population (in particular, due to the huge human casualties).

[11] Note that this would be in accord with initial speculations of Pareto (1896), as it would suggest unchanging character of inequality, not depending on markedly different political and institutional arrangements.

and queueing, widespread consumer price subsidies and price controls, extensive social benefits in kind, or various non-wage benefits of the communist elite, among others.[12]

The overall distributional impact of these items in socialist Poland is difficult to quantify. Different mechanisms frequently operated in different directions and the general assessment has been that they tended to balance on average (as noted, a view shared by many experts: e.g., Bergson 1944,[13] 1984, Morrisson 1984, Atkinson and Micklewright 1992, Milanovic 1998). Take an example of shortages where the resulting 'biases' offset. Shortages are generally viewed as having had equalizing impact, in the sense that some of its corollaries, such as direct rationing or queuing, affected equally all income groups, and as a result monetary inequality overestimated the true income inequality (Bergson 1984, pp. 1058-9). Another phenomenon linked to shortages, but working in the opposite direction, were 'discriminatory marketing practices' (ibid.). Here, the preferential access to closed shops or certain services by the nomenklatura would suggest that monetary inequality underestimated the true inequality.

The contemporary research on income inequality generally saw the underestimation of elites' income as the most problematic issue of the distributional data in the socialist countries, especially for comparative purposes. Several ingenious attempts at quantifying a potential bias caused by non-wage privileges of the communist elites were made, mainly in comparison to the contemporary capitalist countries in Western Europe, but a substantial gap between 'blocks' remained (e.g., Morrisson 1984, Atkinson and Micklewright 1992). Rutkowski, in consequence, has altogether downplayed claims that: "[a claim] that actual inequality was higher than the measured one […] is based more on conventional wisdom than on reliable research findings" (1996, p. 91; Atkinson 2008, p. 323).

However, it could have been the case that non-monetary privileges were shared by a relatively larger group, such as the Communist party members. For example, one could think of privileged access to better housing (amid long waiting lists), to cars and other consumer goods in short supply (e.g., closed shops), or vacation opportunities and foreign travels, among others.[14] This might have produced more substantial impact on dispersion and implied more sizeable underestimation conveyed by the measured inequality. However, our reading of the indirect evidence in the literature prompt us to conjecture that non-wage privileges were overall disproportionally limited to the communist elite (e.g., Matthews 1978). This is not to say that the Communist party membership did not matter *per se*, but it is just that for the majority of the members, the benefits were not large enough to induce a sizeable effect on income inequality. For example, non-wage privileges such as discriminatory sales practices were rather secretive and, at least in this respect, they were most likely 'elitist'. Relatedly, one interpretation of the pronounced egalitarian demands of the Solidarity movement (primarily for social justice) points to the fact that these were largely targeted against elite's privileges. This could suggest that masses linked privileges mainly with elites of the Gierek era. As Flakierski (1986, p. 135) points out: "it was a reaction to the intolerable elitism of the '70s". At the same time, more than a third of party's members joined Solidarity, while a half of them supported it (Grzymala-Busse 2002, p.44).

---

[12] It must be also remembered that many of mentioned distortions should not be taken as inherently "socialist" (take, for example, in Western countries the widespread fringe benefits of the management; or manifestations of shortages in recurring occurrences of housing waiting list, see Atkinson and Micklewright 1992).

[13] Bergson (1944) is the first seminal assessment of inequality in the communist countries, which specifically looked at the inequality of the earnings distribution in the Soviet Union in the 1920s/30s. Bergson concluded that "inequality in money earnings may be studied with the assurance that they are a most important element in Soviet economic life" (p. 48).

[14] However, these need to be distinguished from enterprise provisions of non-wage benefits, which played a prominent role in socialist economies and were not strictly related to the Communist party membership (Atkinson and Micklewright 1992).

Furthermore, a varying importance of non-monetary aspects of inequality could bias inequality comparisons over time. In this respect, the early 1980s economic turmoil is especially pronounced and marked by strong economic imbalances. For example, the widespread rationing in 1981 meant that money inequality might have overestimated the true inequality (as rationing is an undesirable equalizer), yet increased at the same time importance of privileged access to scarce consumer good. Indeed, this is the reason that we put qualifications to a sharp fall of earnings inequality between 1980 and 1982 (documented in Figure 5).

Second, it could have also been the case that surveys did not get the distribution of money incomes right. Specifically, it was sometimes argued that income is not evenly "misreported" along the distribution. For example, a generally held view is that the relatively large informal ("second" or "shadow") economy in socialist Poland was more pervasive at the lower income levels, which implied that higher incomes—largely obtained from the official economy—were better captured in the survey (note that this issue is pertinent to the ongoing debate on international poverty estimation, e.g., see Chen and Ravallion 2010). For example, it is commonly believed that 'moonlighting' as a part of the second economy was more pervasive at the 'bottom' (since time constraints made it difficult for high-income groups, such as managers or specialists) (Flakierski 1986, p. 79). Note that this would entail that our estimates of the full income distribution for 1983-1989 from the survey overestimate the true inequality of living standards. In this respect, our estimates should be seen as the upper bound.

A similar qualification can be made about our estimates of top income shares for the 1956-1990 period, even though the methodology applied should partly account for underestimation of lower incomes: namely, here we relate the reported income of high-income groups (such as the top 1%) to external estimates of the total income from the national accounts, which partly include income from the informal economy such as "income from other self-employment" (*szacunek dochodow netto ludnosci z prowadzenia dzialnosci gospodarczej*) or "income from subsidiary economic activities", among others.

/the comparison with high incomes reported in the 'equalizing tax' (*podatek wyrównawczy*) statistics (see above) indeed suggest that high incomes in our data source (i.e., employer survey) were measured with a higher precision. The comparison suggests a strong consistency between sources, resulting in practically equivalent top income shares.

Finally, note that internationally used inequality measures, including ours, regularly do not account for social benefits in kind provided for free, such as education or health care. However, a specific research on this dimension did not find significantly different distributional impact in communist and contemporary capitalist countries in Western Europe (e.g. Morrisson 1984; see Atkinson and Micklewright 1992, p. 147).

OA.1.7. Poland 1992-2015

Data. Table OA3 below reports used tax publications for the period 1992-2015. It should be noted that tabulations are presented by ranges of taxable income (after deductions) rather than gross income. But the total income is provided for each interval, both for income before and after employee social security contributions. We apply our preferred income concept and adjust interval thresholds using multiplicative factors. The amount of deductions is negligible and should not affect our estimates in any significant way.

Table OA3: Data sources for the period 1992-2015

Informacja dotycząca rozliczenia... od osób fizycznych za 1992 rok	p. 2
Informacja dotycząca rozliczenia... od osób fizycznych za 1993 rok	p. 4
Informacja dotycząca rozliczenia... od osób fizycznych za 1994 rok	Biuletyn Skarbowy 3/1995: p. 6
Informacja dotycząca rozliczenia... od osób fizycznych za 1995 rok	p. 2; Tab. 1.1 (p. 6)
Informacja dotycząca rozliczenia... od osób fizycznych za 1996 rok	Biuletyn Skarbowy 6/1997: p. 3; Tab. 1.1 (p. 5)
Informacja dotycząca rozliczenia... od osób fizycznych za 1997 rok	Biuletyn Skarbowy 6/1998: p. 7; Tab. 1.1 (p. 9)
Informacja dotycząca rozliczenia... od osób fizycznych za 1998 rok	Biuletyn Skarbowy 5/1999: p. 5; Tab. 1.1 (p. 7)
Informacja dotycząca rozliczenia... od osób fizycznych za 1999 rok	Biuletyn Skarbowy 5/2000: p. 9; Tab. 1.3 (p. 11)
Informacja dotycząca rozliczenia... od osób fizycznych za 2000 rok	p. 4; Tab. 1.3 (p. 6)
Informacja dotycząca rozliczenia... od osób fizycznych za 2001 rok	p. 5; Tab. 1.3, (p. 8)
Informacja dotycząca rozliczenia... od osób fizycznych za 2002 rok	p. 5; Tab. 1.3 (p. 8)
Informacja dotycząca rozliczenia... od osób fizycznych za 2003 rok	p. 5; Tab. 1.3 (p. 8)
Informacja dotycząca rozliczenia... od osób fizycznych za 2004 rok	p. 5; Tab. 4.4 (p. 10); p. 23
Informacja dotycząca rozliczenia... od osób fizycznych za 2005 rok	p. 5 ; Tab. 4.4 (p. 10); p. 39
Informacja dotycząca rozliczenia... od osób fizycznych za 2006 rok	p.5 ; Tab. 4.4 (p. 11); p. 40
Informacja dotycząca rozliczenia... od osób fizycznych za 2007 rok	p. 5 ; Tab. 4.4 (p. 11); p. 40
Informacja dotycząca rozliczenia... od osób fizycznych za 2008 rok	p. 5 ; Tab. 4.4 (p. 11); p. 36
Informacja dotycząca rozliczenia... od osób fizycznych za 2009 rok	p. 5 ; Tab. 4.4 (p. 11); p. 36
Informacja dotycząca rozliczenia... od osób fizycznych za 2010 rok	p. 5 ; Tab. 4.4 (p. 12); p. 46
Informacja dotycząca rozliczenia... od osób fizycznych za 2011 rok	p. 5 ; Tab. 4.4 (p. 11); p. 38
Informacja dotycząca rozliczenia... od osób fizycznych za 2012 rok	p. 5 ; Tab. 4.4 (p. 11); p. 34
Informacja dotycząca rozliczenia... od osób fizycznych za 2013 rok	p. 5 ; Tab. 4.4 (p. 11); p. 33
Informacja dotycząca rozliczenia... od osób fizycznych za 2014 rok	p. 5 ; Tab. 4.4 (p. 10); p. 30
Informacja dotycząca rozliczenia... od osób fizycznych za 2015 rok	p. 5 ; Tab. 4.4 (p. 10); p. 30

The tax law has been reformed several times since 1992. Because each such event changed the definition of reported income, accompanying modifications need to be taken into consideration when analysing the tax statistics. There were two major reforms, in 2001 and 2004. In 2001, taxation of capital revenue (interest and dividends) and income from realized capital gains (i.e., from selling company's shares, stocks, derivatives) were introduced. While the former needs to be taxed using the presumptive tax and is not reported in the statistics, the latter is taxed using the linear scale and thus will appear in the published statistics. Note that both were absent from the reports before 2001. The details of the capital income taxation are outlined below. The reform of 2004 introduced an option for business income from non-agricultural business activity (further referred as business income) to be taxed separately at the flat rate. We deal with the assumptions concerning the imputation of the business income taxed at the flat rate to the top income shares in the next subsection. Similarly, capital gains from sales of financial assets can also be taxed at the linear rate. In addition, in 2009 the linear taxation of income from real estate sale has been introduced.

Our baseline definition of income excludes income from realized capital gains from sales of financial assets (taxed at the flat rate using the PIT 38 form) and from real estate sales (taxed at the flat rate using the PIT 39 form). The main reason why we do not impute these sources is that the taxation of income from capital gains sales of financial assets has been introduced in 2001, and capital gains from real estate sales in 2009. Comparable information for the earlier periods is not available. We therefore face a trade-off between the

coverage of the post-2001 series and cross-time comparability. Since our focus is on the evolution of inequality in Poland, we believe that excluding these sources is a better choice. This is also in line with the World Income Lab guidelines. In the on-going project (Bukowski et al. 2021) we use the universe of individual tax returns to estimate the distribution for the definition of income including realised income from capital gains from sales of financial assets and capital gains from real estate sales. The preliminary results show that the inclusion of these sources has a negligible effect on the estimated level and growth of inequality. This could be because these sources of income are negligible. The tax statistics show that, for instance, between 2004 and 2013 the income from capital gains was less than 1% (min 0.5%, max 2%) of the total income.

Agriculture income, in general, is not taxed using PIT and thus does not appear in the tax data. The only exception is income from the specialized branches of agriculture, which is a category of agriculture activities, which operation and income do not generally depend on land size (Dziemianowicz 2007; Tyszka et al. 2016). Such activities include, for instance, greenhouse horticulture, animal husbandry, poultry farming. The taxation of specialized agriculture has been introduced together with PIT in 1992, however it applies to holdings above a certain size.

Merging income across tax regimes after 2004. Poland engaged in the flat tax reform in 2004. In comparison to some other countries in Central and Eastern Europe that introduced a flat income tax, the extent of the reform in Poland was less comprehensive and consisted 'only' in the introduction of the flat rate option for certain categories of personal income. Most importantly, individuals obtaining business income could after that choose between taxation of this income separately at the flat rate or at the progressive scale with the rest of their income as before.[15]Until 2009 there were three brackets in the progressive schedule with the respective marginal rates of 19, 30 and 40 percent, and afterwards, they were reduced to two with the respective marginal rates of 18 and 32 percent.

All taxable income, even below the tax exemption threshold, should be reported. Taxpayers using only the progressive scale submit only one tax form (PIT 36 or PIT 37, depending on the source of income). In order to use the flat tax for business, an additional form (PIT 36L) must be submitted. Similarly procedure applies for either income from capital gains on sale of financial assets or real estate sales (PIT 38 and PIT 39 respectively). Therefore, one individual might appear several times in the tax reports, but will only appear once in the progressive tax part.

As a result, the personal income tax statistics has provided distinct reports for the tax returns submitted under the respective tax schedules from 2004 onwards. This has raised a number of methodological challenges when merging the data from the two reports. As a first step in the merging procedure, it should be acknowledged that choosing a flat rate option entails a trade-off (Kopczuk 2012), because on the one hand, the high-income individuals could benefit from lower marginal tax rate, but on the other, it would imply a broader tax base since they would thus give up the right to tax allowances and tax credits as well as the option of joint filling for spouses. This trade-off is presented in Figure OA1 below. It is a replication from Kopczuk (2012, Fig. 0), who explains in detail the incentives behind opting for flat tax rate regime, and the following discussion is closely based on his exposition.

[15] This option has been allowed additionally for rental income.

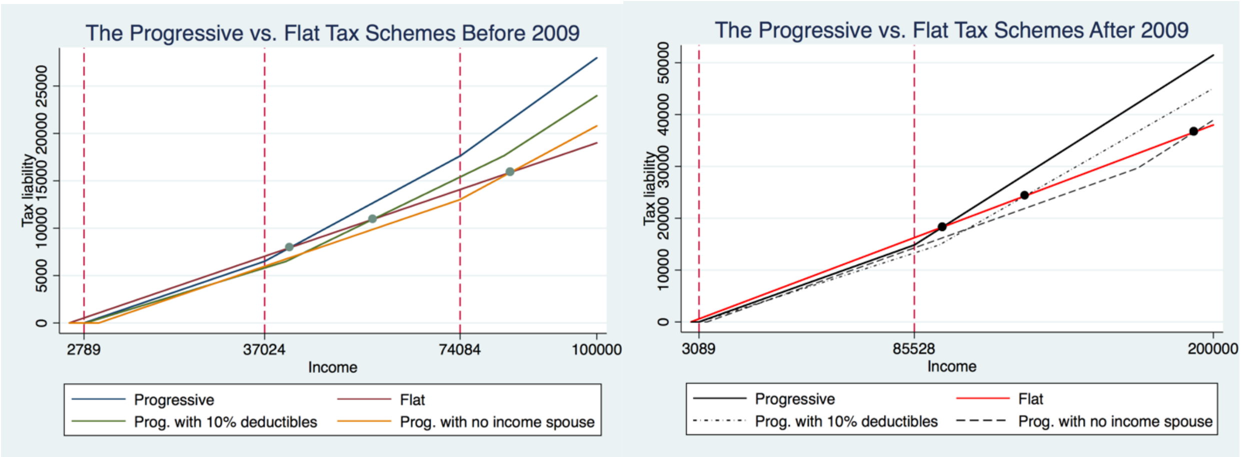

Figure OA1: Tax obligations in the progressive and flat tax regimes

The dashed vertical lines indicate the tax-free threshold and the bracket thresholds of the progressive schedule. Black lines indicate the tax liability under the progressive tax rule when using the tax credit only (the solid line), the tax credit and taxable income deductions (the dash-doted line), and previous plus the benefits of filling jointly with no income spouse (the dashed line). The red line indicates tax liability under the flat rate schedule. The tax optimizing behaviour suggests that taxpayers would choose the flat rate option only if their business income exceeds a certain breakeven point (black points) where the benefits of the lower marginal tax rate outweigh the associated costs of losing tax preferences, and the overall tax liability is consequently reduced. Most importantly, the figures show that the flat rate benefits become dominant only at the income levels above the middle bracket threshold and rise depending on the use of available tax preferences. After the reform of 2009, there are only two income brackets (indicated by the vertical dashed lines). In all the income scenarios outlined above, the breakeven points lie above the top income thresholds. In other words, it is profitable to switch to the flat regime only if the business income exceeds the top bracket threshold.

Merging income across the tax regimes is straightforward after 2009, as we simply join the income taxed using the flat tax to the income from the progressive top bracket. The situation is more complicated before 2009 when the break-even point of switching to the flat tax regime might be located within the middle bracket. Fortunately, we can support some important assumptions by the insight from the descriptive statistics of the income tax microdata. These are provided by Kopczuk (2012) and Bukowski et al. (2021), who use the individual-level personal income tax returns covering the 2002-2015 period.

First, using descriptive statistics from Kopczuk (2012, Table 1), we estimate that 40% of flat tax fillers have their income within the range of the middle bracket of the progressive schedule. Then, by assuming that these individuals earn middle bracket's average income (which is likely to be under-estimation), this results that business income of these flat tax fillers accounts for 15% of the total business income taxed at the flat rate (a proportion that is remarkably constant throughout the years). The remaining 60% of flat tax fillers are placed in the top bracket of the progressive schedule with the remaining 85% of total business income taxed at the flat rate.

This number is also supported once we look at changes in the reported flat tax income just before and after the reform of 2009. The rationale is that the reform motivated people with the business income within the range of the previous middle bracket, to switch from the flat to the progressive regime. Assuming

a counterfactual increase of business income by 6%, the comparison reveals a drop in the flat tax income of around 15%.

Second, the reports of Ministry of Finance in 2017 estimate that around 65% of the individuals reporting their income in the flat regime report also income in the progressive schedule. Before 2009, we assume that 60% of these individuals are reported in the bottom bracket of the progressive schedule, and 40% in the middle bracket. Based on numbers from Kopczuk (2012, Table 1 and Table 2), we move 60% of individuals from the bottom bracket to the top bracket and the remaining 40% to the medium bracket. Similarly, we move 60% of individuals from the middle bracket to the top bracket and leave remaining in the middle bracket. After 2009, we move 80% to the top bracket (with 20% of the bottom bracket's average income) and keep 20% in the bottom. In all cases, we assume that the progressively-taxed income of overlapping individuals is 60% of the bracket's average income.

Joint taxation. Married couples and single parents have a right to submit a joint tax form. An important condition is that neither of spouses (or a single parent) taxes his/her income using the flat rate or the presumptive tax. Since the joint report yields tax benefits, married individuals (and single parents) might be thus more reluctant to use the flat regime or the presumptive tax, than unmarried people (without kids). In the case of married couples, the reported joint taxable income is a sum of each spouse's income divided by two. A similar construction is used for the single parents, with an exception that the sum consists of parent's and child's income (if any). The tax publications report the number of taxpayers in each bracket after the couple split.

Population and Income Control. See the Online Appendix 2.1 and 2.2

Income Control. In our benchmark series, total income is estimated directly by combining survey and tax data. However, as a robustness check, we estimate top income shares for the 1992-2015 period using personal income tax tabulations and independently estimated total income denominator. We add the following items to approach the aggregate that corresponds as closely to the concept of income reported in the tax statistics:

(i) wages and salaries received by households, net of employers' social security contributions, plus (ii) social security benefits in cash, plus (iii) 50% of profits of household unincorporated enterprises (taken as household operating surplus net of depreciation, net of primary income in agriculture and net of imputed rents of owners' occupiers), plus (iv) withdrawals from income of quasi-corporations received by households plus 30% of retained earnings of non-financial corporations.

Income denominator obtained this way results on average in 80 per cent of households' primary incomes. We take only half of the income of household's unincorporated enterprises because the Central Statistical Office publishes the national accounts figures corrected for the concealed activity, which is in the same manner concealed from the tax authorities. Moreover, the scope of the non-observed economy was especially worrisome for the transition economies. According to official estimates, concealed activity in Poland has been the most prevalent in the household sector, for example accounting for as much as 7 per cent of GDP in 1998 (United Nations 2003, p. 188).

It should be noted that in the Polish national accounts, enterprises smaller than ten employees are included in the household sector, while those with ten and above employees in the non-financial corporate sector. We take 'withdrawals from income of quasi-corporations' as a measure of distributed income of unincorporated enterprises in the corporate sector, as the CSO only estimates 'withdrawals' paid by non-financial corporations, and add 30 per cent of retained earnings of non-financial corporations (as unincorporated firms are as 'pass-through' entities taxed with their whole profits under PIT).

For years 1992-1994 we lack comparable external controls for total income to use the method described above. Instead, we use an alternative method to arrive at total income control, which starts from the total income of taxpayers reported in the tax statistics and add the total income of 'non-filers' (Atkinson 2007). Using this approach depends on the proportion of the population that files income tax returns. Today in Poland most of the population actually files personal income tax (either by themselves or by tax remitters such as employers or social insurance institutions), on average close to 90 per cent of our reference for the total population, which makes in theory this method a reliable alternative. For years 1992-4 we estimate total control for income by assuming that the total reported income of filers makes 85 per cent of the total income and consequently the total income of non-filers 15 per cent of the total income. This proportion is chosen based on the proportion of the income of filers in the total income in the late 1990s. The data on sectoral national accounts is available from the CSO of Poland and Eurostat.

Online Appendix OA2:
The Full Distribution of Income, 1983-2015; Methodology and Data Sources

We combine household surveys and income tax data in order to construct new income distribution series in Poland for the 1983-2015 period. More precisely, we use tax data on high-income taxpayers to correct the top of the survey distribution. This way, we aim to provide more reliable estimates of the full income distribution.

The general methodology we use to combine survey and fiscal data consists of several steps:

1. In the first step we use the raw survey tabulations and generalized Pareto interpolation techniques (Blanchet et al. 2017) to estimate series on the distribution of survey income by generalized percentiles (g-percentiles). Survey tabulations simply refer to our estimates of income distributions from the survey microdata (see the next point for the specific surveys we used). These tabulations are then applied in the G-pinter tool to produce g-percentiles (using generalized Pareto interpolation), which provides consistent summary of the distribution (https://wid.world/gpinter/).

2. We do the same for the tax data on top income earners (described in Online Appendix OA.1).

3. Having estimated the percentiles from the two sources, for each year we calculate the correction factors defined as a ratio between the same percentiles from the tax data and from the surveys, for instance, for the 95th percentile $CF95 = P95^{tax}/P95^{survey}$.

4. In the next step, we use the calculated correction factors to correct upwards the survey raw percentiles and obtain corrected estimates of the full distribution of fiscal income. We assume that survey data provide a reasonable description of the income distribution below the 85th percentile. We find this assumption fairly robust by checking different variant series using the 80th or 90th percentile. On the other hand, we take that tax data is accurate above the percentile corresponding to the first available income threshold in tax tabulations (generally corresponding to the 95th percentile). We then apply the piecewise-linear correction factors between these percentiles. More precisely, the factor between survey and fiscal income is 1 for P85 and rises piecewise-linearly to the observed factor in the tax and survey data (we find that the profile of the fiscal/survey uprate ratio (piecewise-linear or linear) between P85 and P95 has limited impact, since the most important correction happens above P95).

In the remaining part of this appendix, we discuss the survey data and definitions of income.

OA.2.1. Survey data

We use the household survey data used for the entire 1983-2015 period. For the 1980s, we use aggregated the Polish Household Budget Survey (HBS) (*Badanie Budżetów Gospodarstw Domowych*) data from Atkinson and Micklewright (1992, Tables PI1 and PI2). The authors provide tabulations of the individual distribution of household income per capita by combining the distribution of income for four types of households (worker, mixed, farmer, and pensioner households) from the official HBS reports. The

tabulations are organized by eight income groups, providing in each the number of individuals and the mean income.[16] For the 1992-2004 period, we use harmonised HBS microdata from the Luxembourg Income Study (LIS). The data are available for 1992, 1995, 1999, 2002 and 2004, (survey years). We impute the data for the missing in-between years in two steps. First, we upgrade thresholds and average incomes in two adjacent survey years by the ratio of average fiscal income per adult in the survey and the missing year (obtained from National Accounts). Second, we apply linear interpolation between two upgraded estimates to obtain thresholds and average income in missing years. For the period 2005-2015 we use the EU Statistics on Income and Living Conditions (EU-SILC) collected by Eurostat.

We follow the Distributional National Accounts (DINA) Guidelines (Alvaredo et al. 2020) and define our unit of analysis as individuals aged 20 and above. This allows us international comparability. It is reasonable to take adult individuals as the control total for population, since adults are the main earners of income. The rationale for choosing an age cut-off of 20 (instead of 15 or 18) is that "young people enter the labour force later today" (Atkinson 2007, p. 27). As Atkinson (2007) pointed out, an age cut-off of 20 provides a lower bound estimate of top income shares (for example, an age-cut-off of 15 would give an upper bound). However, he shows that the effect of choosing the different population cut-off is rather modest and does not affect the distributional estimates in any significant way.

Household income in survey is equally split between all adults who belong to the same household, because the HBS reports a substantial part of the household income only at the household level. However, the tax unit in the tax statistics is individual whose income is not necessarily equal to income of other adults belonging to the same household. A notable exception is when married individuals opt for joint taxation, in which case the tax data relates to income split within couples. We should bear in mind that when combining survey and tax data we make implicit assumptions that high-income individuals in tax data are either singles or they belong to a nuclear family and use joint taxation.

According to the PIT reports, the majority of the eligible taxpayers used joint taxation. In 2000, almost 62% taxpayers used joint taxation, after the linear taxation has been introduced in 2004, 58% of those using progressive schedule used this option, and in 2016 the share fell to 48%. In comparison, the share of married in working age population was 64% in 2000 and 55% in 2016 (GUS 2017). Therefore, a substantial share of marriages used joint taxation, and the downward trend in the share of joint fillings could be due to a falling share of married people throughout this period.

The LIS data on household income for Poland is net of labour and pension income tax withheld at source. We impute pre-tax labour income by grossing the net income using the official tax rates and adjusting for the joint taxation of married couples. As social security contributions are deducted from the taxable income, whenever possible we add them to the grossed-up income. Business income for which tax is paid directly by individuals, is recorded as gross. The EU-SILC data is reported gross of taxes and social security contributions.

As already mentioned, the baseline estimates for the period after 2005 are based on EU-SILC. This is a preferable source as it is available annually and the data on income is reported by individuals from their annual tax forms (the survey and the tax filling took place in the same month - April). As such it is more consistent with the tax data. HBS is based on self-reported income from a previous month, which might be

[16] One should also bear in mind the survey is fully representative since 1993. Previously, it omitted police, army, and non-agricultural private sector (Milanović 1999, Tab. A1).

problematic for seasonal sources of income, such business or agriculture. Therefore, income reported in EU-SILC is more consistent with the tax data, which we use to estimate the top income shares.

As a robustness check, Figure OA2 focuses on the period after 2005 and compares the baseline estimates (EU-SILC) with the alternative series estimated using the HBS survey. The two series are almost indistinguishable until 2007. After 2007, the alternative series show somehow higher level of inequality compared to the baseline: the top 10% income share in HBS is higher by around 1.5 pp, the middle 40%. by around 1pp lower, and the bottom 50% by around 0.5pp lower. Importantly, the two series exhibit almost the same evolution through time. These results are reassuring for three reasons. First, the merger of HBS and EU-SILC in the baseline series does not lead to a discontinuous change after 2004, since the baseline is very similar to the HBS series in the period 2005-2007. Second, the baseline series provide a more conservative picture of the level and growth of income inequality in Poland after 2000. Third, the comparison also validates our method of transformation of net income in HBS to gross numbers, as it shows little difference between the "grossified" HBS series and the (real) gross EU-SILC series. The potential problem with grossing-up the net incomes (labour and pensions) and missing tax deductions should mostly concern the estimates of the bottom 50% income share. With this respect, it is reassuring that the HBS and EU-SILC series are similar to each other across the entire period.

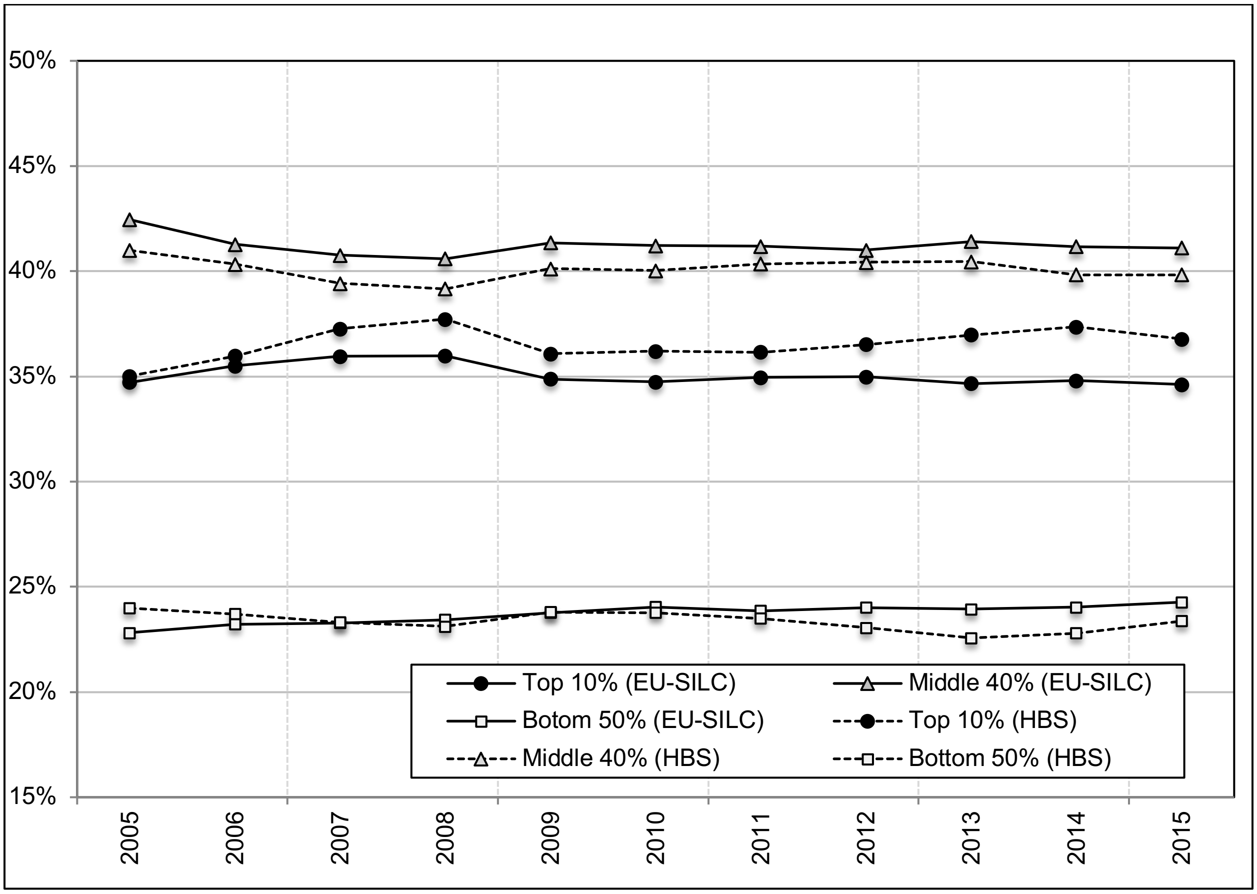

Figure OA2: Comparison of the baseline estimates (EU-SILC) with the alternative estimates (HBS).
Source: own calculations using EU-SILC, HBS and the tax data.

OA.2.2. Definition of income

When joining the survey and tax data, we produce the distribution of the fiscal income. This is the same income concept used to construct top income shares series. Several caveats, however, must be mentioned.

The income reported in the tax data does not cover non-specialized agricultural activities, therefore we implicitly assume that there are no top income taxpayers from these sectors. If this assumption is not correct, we will underestimate the true level of inequalities, as the correction coefficients calculated using the tax data are biased downward. This is because the very top g-percentiles from the tax data do not include income from non-specialized agriculture. Nevertheless, we believe that the scale of the potential bias is negligible for two reasons. First, income from the specialized branches of agriculture is taxed using the standard PIT and thus appears in our tax data. It can be shown from the Farm Accountancy Data Network (FADN) data that the richest farmers in Poland are operating mainly in the specialized sectors (i.e., poultry farming, greenhouse horticulture and cows) (results available upon request). It suggests, therefore, that the high-income farmers are taxed using PIT and thus appear in the tax data. Second, using the FADN data we estimate the upper bound of the potential missing non-specialized agriculture income from the top 5%. The results, which are available upon request, show that missing agriculture income in 2015 is less than PLN 2 billion, while the total income of the top 5% reported in the tax data is PLN 300 billion! In 2004, the estimated missing income is PLN 1.1 billion, compared to nearly PLN 120 billion in the top 5%. These results show the potential distortion coming from omitting rich farmers from non-specialized agriculture is negligible.

It is worth noting that income concept in survey data during the socialist period (1983-1989) is that of post-tax (or disposable) income. However, as personal income taxes were negligible during the socialist period and employees did not contribute to social security from their gross wage, there is no practical difference which of the two concepts is used.[17]

Further distinction needs to be made between fiscal and national income (as standardly defined: GDP minus consumption of fixed capital plus net foreign income (SNA 2008)). A major difference is due to the fact the national income includes in addition tax-exempt capital income, such as undistributed corporate profits or imputed rents. At this stage, we provide only the distribution of fiscal income, but, in general, it has been found that the fiscal correction (using income tax data) accounts for the bulk of upward correction of raw survey inequality, and further adjustment for the distribution of tax-exempt capital income has showed to be of relatively limited impact (see, e.g., the studies on China and Russia; Piketty et al. 2019; Novokmet et al. 2018). But in order to allow an international comparison, we scale fiscal income distribution to the national income totals by proportionally upgrading thresholds and average incomes for each percentile of the fiscal income distribution.

The National Accounts data on population size, average national income and inflation are presented in Table OA4. Our estimates of percentiles and average national income are presented in Table OA5.

[17] Milanović (1999, pp. 322-3), for example, points out that the difference between pre- and post-tax income was less than 1%.

Table OA4: Population, Average National Income and Inflation; Poland 1992-2015

Year	Adults (20+) population (in thd.)	Total population (in thd.)	(1)/(2) %	Average nat. income (PLN)	Average nat. income (2010 PLN)	CPI 2010=100
1992	25,900	38,203	68%	3,839	21,567	18
1993	26,073	38,239	68%	5,273	21,611	24
1994	26,254	38,265	69%	8,000	24,615	33
1995	26,463	38,284	69%	10,910	26,163	42
1996	26,695	38,294	70%	13,763	27,581	50
1997	26,948	38,290	70%	16,627	28,967	57
1998	27,198	38,277	71%	19,199	29,905	64
1999	27,463	38,263	72%	21,075	30,632	69
2000	27,348	38,254	71%	23,448	30,934	76
2001	27,617	38,242	72%	24,157	30,234	80
2002	27,886	38,219	73%	24,687	30,328	81
2003	28,183	38,191	74%	25,067	30,532	82
2004	28,505	38,174	75%	25,610	30,129	85
2005	28,816	38,157	76%	27,743	31,962	87
2006	29,099	38,126	76%	29,437	33,527	88
2007	29,315	38,116	77%	31,752	35,319	90
2008	29,506	38,136	77%	35,192	37,518	94
2009	29,686	38,167	78%	35,962	36,922	97
2010	29,684	38,530	77%	37,450	37,450	100
2011	29,847	38,538	77%	40,448	38,780	104
2012	29,999	38,533	78%	41,734	38,643	108
2013	30,114	38,496	78%	42,403	38,866	109
2014	30,189	38,479	78%	43,597	39,924	109
2015	30,266	38,437	79%	45,177	41,792	108

Table OA5: Percentiles and Average National Income; Poland 1983-2015

Year	Average	P50	P90	P99	P90-100	P99-100
1983	23	21	36	69	50	97
1984	28	26	44	89	63	132
1985	34	30	54	104	75	141
1986	42	38	67	129	94	174
1987	54	49	85	158	117	232
1988	95	85	151	268	203	393
1989	382	340	619	1,183	862	1,682
1992	3,839	3,141	6,060	13,624	10,154	29,774
1993	5,273	4,311	8,353	18,405	14,341	44,568
1994	8,000	6,387	12,551	32,840	23,717	80,195
1995	10,910	8,517	17,019	54,138	34,958	122,201
1996	13,763	10,805	22,463	65,986	43,234	127,753
1997	16,627	13,139	26,707	73,140	51,481	166,322
1998	19,199	14,940	30,317	92,176	61,313	205,896
1999	21,075	16,615	33,016	104,300	65,414	212,856
2000	23,448	18,055	37,750	123,902	76,087	233,221
2001	24,157	18,667	39,553	110,585	77,655	248,314
2002	24,687	18,787	40,528	123,623	81,709	260,302
2003	25,067	18,942	42,202	134,893	83,802	240,909
2004	25,610	19,203	41,658	129,810	87,167	303,435
2005	27,743	20,264	47,024	139,639	96,335	338,032
2006	29,437	21,270	47,907	153,508	104,488	380,540
2007	31,752	22,891	50,481	166,033	114,147	450,430
2008	35,192	25,135	56,527	187,752	126,619	482,907
2009	35,962	26,024	58,636	193,547	125,439	434,418
2010	37,450	27,502	61,078	203,396	130,074	447,597
2011	40,448	29,670	65,223	218,643	141,364	490,169
2012	41,734	30,628	67,303	224,714	145,986	504,183
2013	42,403	31,167	70,031	226,775	146,963	506,030
2014	43,597	32,142	70,231	241,881	151,719	537,755
2015	45,177	33,414	71,919	248,180	156,405	563,009

Note: current PLN

Online Appendix OA3:
Distribution of Earnings

For the interwar period, estimates of the upper part of distribution are based on annual enterprise surveys of workers in medium-sized and large enterprises in processing and energy industries (those with more than 20 employees; divided into three groups: enterprises up to 49 employees, enterprises with 50 to 199 employees, and enterprises above 200 employees). The Central Statistical Office and the Ministry of Industry and Trade conducted the survey quarterly in the months of February, May, August and November. Results were published in the form of tabulations ranged by the weekly wage. Published tabulations also provide earning bands by gender, by the size of the enterprise, by employees covered by collective agreements, by specific industry and by regions.

It should be noted that indicated dispersion of the upper part of the wage distribution should be seen as a lower bound since small enterprises not covered by the survey had generally paid much smaller wages (Landau 1933, p. 118). However, Czajkowski (1934) estimated earnings distribution for all workers in 1934. Dispersion at the top is higher than in the case where only industrial workers in the middle and large enterprises were covered, in the first place because of the now lower median wage. This corresponds to the above-mentioned Landau's observation.

In socialist Poland the enterprise survey was conducted annually in the period from 1949 until 1989. The survey assessed earnings of full-time employees in September in socialized sector covering state-owned and cooperative enterprises. This covered around two-thirds of the total workforce, while excluded were self-employed and those working in the private sector. The predominant part of self-employed and employees in private sector was in agriculture (Atkinson and Micklewright 1992, p. 257). The survey only included full-time workers in the month of September. Definition of earnings referred to gross monthly earnings (inclusive of bonuses and allowances) in the period from 1955 until 1970, while from 1970 the concept of net earnings was used instead (Atkinson and Micklewright 1992, p. 257). However, Figure 5 shows that in 1970, for which both concepts were published, upper percentiles show markedly higher level (as proportion to median) when using the gross concept (Atkinson 2008, p. 320). The private sector is also covered since 1991 (firms with more than six employees; Atkinson 2008, p. 320), and the gross concept of earnings is used.

Online Appendix 4: Tables

Table OA6: Top income shares (in %) in the Partitioned Poland 1890s -1917

Top:	1%	0.5%	0.1%	0.01%	1-0.1%	0.1-0.01%
			Galicia			
1898	11.7	8.9	4.6	1.6	7.1	2.9
1899	11.3	8.4	4.1	1.5	7.1	2.7
1900	12.8	9.7	5.2	2.3	7.6	2.8
1901	14.3	10.9	5.9	2.7	8.4	3.2
1902	13.6	10.1	5.1	2.0	8.5	3.1
1903	13.3	9.9	4.8	1.8	8.5	3.0
1904	13.4	10.0	5.0	1.8	8.5	3.1
1905	12.9	9.6	4.8	1.8	8.1	3.0
1906	12.5	9.4	4.8	-	7.7	-
1907	13.3	9.8	4.9	1.8	8.4	3.0
1908	13.3	9.8	4.7	1.1	8.5	3.6
1909	12.8	9.3	4.4	1.5	8.4	2.9
1910	11.7	8.6	4.0	1.3	7.7	2.7
1911	12.4	9.1	4.3	1.5	8.1	2.8
1912	11.6	8.5	4.1	1.4	7.5	2.7
			Province of Posen and Western Prussia			
1892	10.4	7.4	3.5	-	6.9	-
1893	10.3	7.3	3.4	-	6.9	-
1894	10.3	7.3	3.5	-	6.9	-
1895	10.4	7.4	3.5	-	6.8	-
1896	10.5	7.6	3.6	-	6.9	-
1897	11.0	7.9	3.8	-	7.1	-
1898	11.5	8.5	4.2	-	7.3	-
1899	12.2	9.1	4.8	-	7.4	-
1900	12.2	9.1	4.7	-	7.5	-
1901	11.8	8.7	4.5	-	7.4	-
1902	11.5	8.4	4.1	-	7.4	-
1903	11.5	8.5	4.3	-	-	-
1904	11.9	8.8	4.5	-	-	-
1905	12.4	9.5	5.0	-	7.4	-
1906	12.4	9.5	5.0	-	7.4	-
1907	12.5	9.6	5.1	-	7.3	-
1908	12.4	9.5	5.1	-	7.2	-
1909	12.3	9.5	5.2	-	7.2	-
1910	12.5	9.6	5.2	-	7.4	-
1911	12.8	9.8	5.4	-	7.4	-
1912	13.0	10.0	5.4	-	7.5	-
1913	13.6	10.5	5.7	-	7.9	-
1914	14.3	11.3	6.4	-	7.9	-
1915	17.3	14.2	8.8	-	8.5	-
1916	20.1	17.0	11.1	-	9.0	-
1917	20.0	17.0	11.2	-	8.7	-

Source: authors' computation based on income tax statistics. Distribution of fiscal income among tax units.

Table OA7: Top income shares (in %) in Poland 1925-2015

Top:	5%	1%	0.5%	0.1%	0.01%
1925	-	10.5	7.2	3.1	0.9
1926	-	10.8	7.6	3.3	0.9
1927	-	11.8	8.4	3.6	1
1928	-	11.9	8.6	4	1.2
1929	25.3	12.0	8.5	3.8	1.2
1930	-	-	-	4.3	1.3
1931	-	-	-	-	-
1935	-	-	11.6	5.3	1.6
1936	-	15.6	11.3	5.1	1.5
1992	18.7	7.8	-	-	-
1993	19.4	8.4	-	-	-
1994	22.0	9.9	-	-	-
1995	24.4	11.0	-	-	-
1996	22.9	9.4	-	-	-
1997	22.8	10.3	-	-	-
1998	24.0	11.0	-	-	-
1999	23.3	10.4	-	-	-
2000	24.2	10.5	-	-	-
2001	23.7	10.9	-	-	-
2002	24.6	11.2	-	-	-
2003	24.5	10.3	-	-	-
2004	25.9	12.0	-	-	-
2005	26.7	12.9	-	-	-
2006	27.9	14.0	-	-	-
2007	27.2	14.5	-	-	-
2008	26.0	14.4	-	-	-
2009	25.9	12.8	-	-	-
2010	26.0	12.4	-	-	-
2011	25.5	12.5	-	-	-
2012	25.6	12.2	-	-	-
2013	26.0	12.3	-	-	-
2014	26.0	12.6	-	-	-
2015	25.9	12.9	-	-	-

Source: authors' computation based on income tax statistics. Distribution of fiscal income among tax units.

Table OA8: Income shares (in %) in Poland 1983-2015

	Bottom 50%	Middle 40%	Top 10%	Top 5%	Top 1%	Top 0.1%
1983	31.3	46.8	21.8	13.3	4.2	0.8
1984	30.5	46.9	22.6	14.1	4.7	1.0
1985	31.4	46.5	22.2	13.4	4.2	0.8
1986	31.2	46.6	22.3	13.5	4.2	0.7
1987	31.1	47.2	21.8	13.2	4.3	0.9
1988	32.2	46.4	21.4	12.8	4.1	0.9
1989	30.9	46.6	22.6	13.8	4.4	0.9
1992	29.3	44.3	26.4	17.7	7.8	2.9
1993	28.7	44.2	27.2	18.4	8.5	3.7
1994	27.3	43.0	29.6	20.8	10.0	4.0
1995	25.9	42.0	32.0	23.1	11.2	4.1
1996	26.2	42.4	31.4	21.7	9.3	2.9
1997	26.5	42.6	31.0	21.6	10.0	3.7
1998	26.2	41.9	31.9	22.7	10.7	3.9
1999	26.6	42.3	31.0	22.1	10.1	3.3
2000	25.8	41.8	32.4	22.9	9.9	3.0
2001	25.7	42.2	32.1	22.4	10.3	3.7
2002	25.1	41.8	33.1	23.3	10.5	3.6
2003	24.7	41.9	33.4	23.2	9.6	2.7
2004	24.3	41.7	34.0	24.5	11.8	4.5
2005	22.8	42.5	34.7	25.1	12.2	4.9
2006	23.2	41.3	35.5	26.0	12.9	5.3
2007	23.3	40.8	35.9	26.8	14.2	6.2
2008	23.4	40.6	36.0	26.5	13.7	5.8
2009	23.8	41.3	34.9	25.3	12.1	4.5
2010	24.0	41.2	34.7	25.1	12.0	4.3
2011	23.9	41.2	34.9	25.5	12.1	4.5
2012	24.0	41.0	35.0	25.5	12.1	4.5
2013	23.9	41.4	34.7	25.1	11.9	4.4
2014	24.0	41.2	34.8	25.5	12.3	4.5
2015	24.3	41.1	34.6	25.4	12.5	4.4

Source: authors' computation based on combined household surveys and income tax statistics. Distribution of pre-tax national income (before taxes and transfers, except pensions and unemployment insurance) among equal-split adults. National income from World Inequality Database (https://wid.world/)

Table OA9: Top income shares (in %) in the Prussian Poland 1892-1918

	Top 1%			Top 0.1%		
Province:	West Prussia	Province of Posen	Silesia	West Prussia	Province of Posen	Silesia
1892	10.4	10.3	15.0	3.15	3.63	6.95
1893	10.2	10.2	14.6	3.03	3.54	6.68
1894	10.3	10.2	14.7	3.15	3.52	6.70
1895	10.3	10.3	14.9	3.14	3.62	6.76
1896	10.3	10.6	15.2	3.08	3.85	6.91
1897	11.0	10.9	15.7	3.57	3.87	7.16
1898	11.4	11.6	16.0	3.89	4.37	7.35
1899	11.7	12.6	16.6	4.13	5.24	7.88
1900	11.7	12.6	16.8	4.08	5.20	8.04
1901	11.2	12.3	16.7	3.75	4.92	8.08
1902	11.0	12.0	15.9	3.49	4.60	7.25
1903	-	12.0	-	-	4.78	-
1904	-	12.6	-	-	5.11	-
1905	11.6	13.4	16.2	3.96	5.74	7.52
1906	11.4	13.6	16.4	3.91	5.88	7.71
1907	11.3	13.8	16.3	3.92	6.02	7.77
1908	11.3	13.6	16.3	4.06	5.93	7.85
1909	11.1	13.6	16.1	3.88	6.11	7.90
1910	11.3	13.8	15.9	3.93	6.12	7.54
1911	-	-	-	-	-	-
1912	-	-	-	-	-	-
1913	12.2	14.8	16.3	4.48	6.70	7.75
1914	12.5	15.8	16.5	4.73	7.70	8.05
1915	15.1	19.4	18.1	-	10.46	9.11
1916	17.4	22.6	23.8	-	13.23	14.09
1917	17.2	22.4	23.6	-	13.02	13.52
1918	13.9	-	20.5	-	-	11.22
		-			-	

Source: authors' computation based on income tax statistics.

Table OA10: Income growth and inequality in France 1983-2014 and Russia 1989-2016

Income group (distribution of per adult pre-tax national income)	Poland		France		Russia	
	Total cumulated real growth 1989-2015	Share in total macro growth 1989-2015	Total cumulated real growth 1983-2014	Share in total macro growth 1983-2014	Total cumulated real growth 1989-2016	Share in total macro growth 1989-2016
Full Population	**73%**	**100%**	**35%**	**100%**	**41%**	**100%**
Bottom 50%	**31%**	**13%**	**31%**	**21%**	**-20%**	**-15%**
Middle 40%	**47%**	**30%**	**27%**	**37%**	**15%**	**16%**
Top 10%	**190%**	**57%**	**49%**	**42%**	**171%**	**99%**
Top 1%	458%	24%	33%	21%	429%	56%
Top 0.1%	1019%	9%	98%	21%	1054%	34%
Top 0.01%	2273%	3%	133%	8%	2134%	17%

Source: Poland: Authors' computation (see section 2). Distribution of pre-tax national income (before taxes and transfers, except pensions and unemployment insurance) among equal-split adults. France: see Garbinti et al. (2017) (Table 2b). Russia: see Novokmet et al. (2018a) (Table 2).

Online Appendix 5: Figures

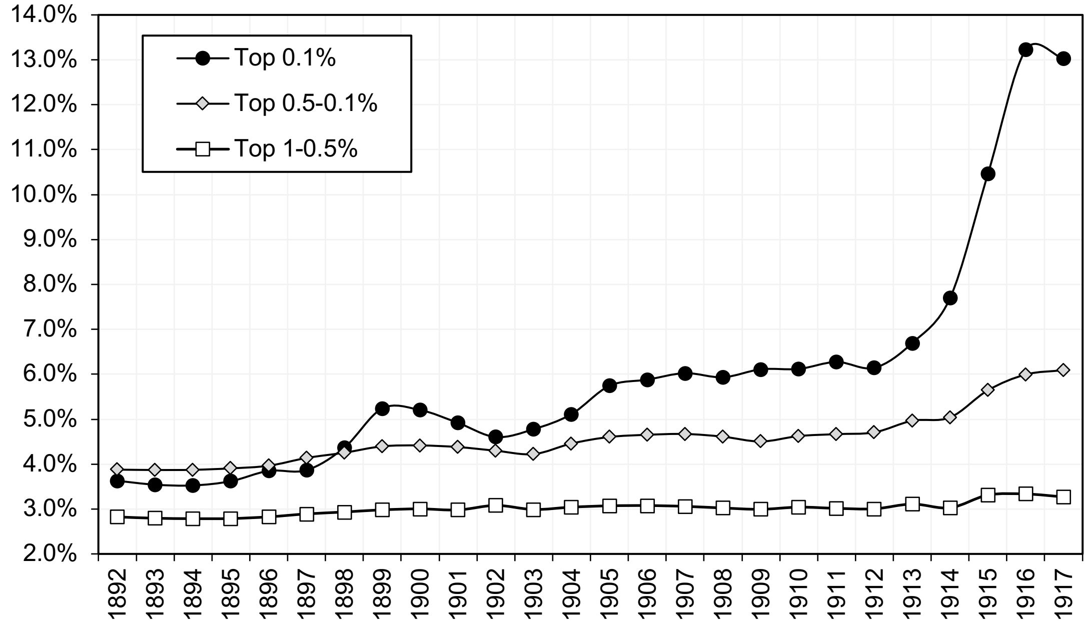

Figure OA1: The Province of Posen – decomposition of the top percentile
Source: authors' computation based on income tax statistics.

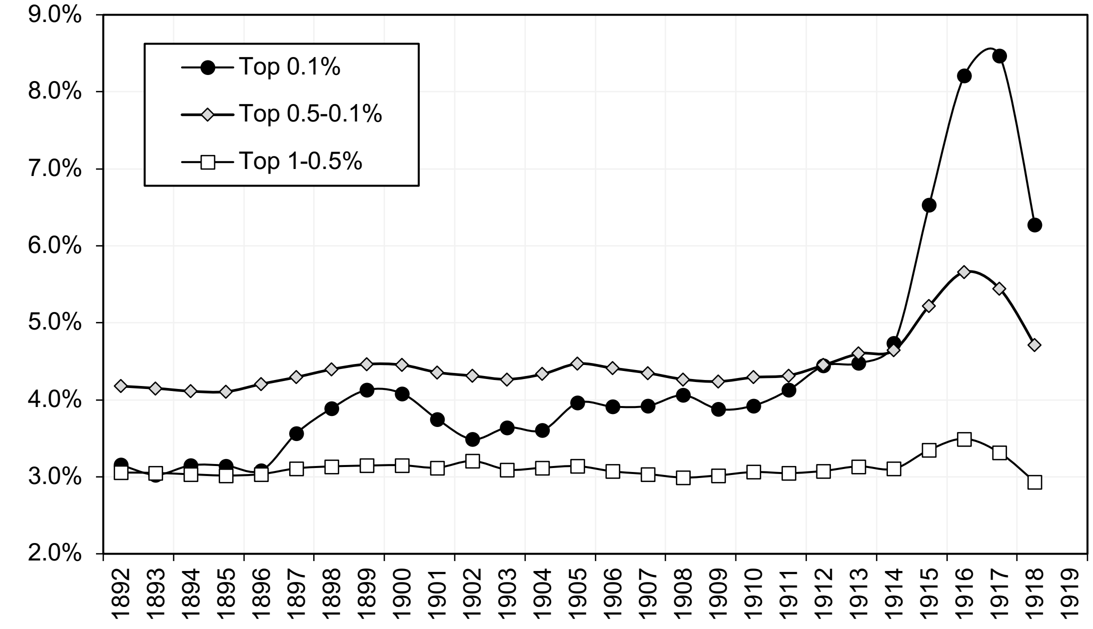

Figure OA2: West Prussia – decomposition of the top percentile
Source: authors' computation based on income tax statistics.

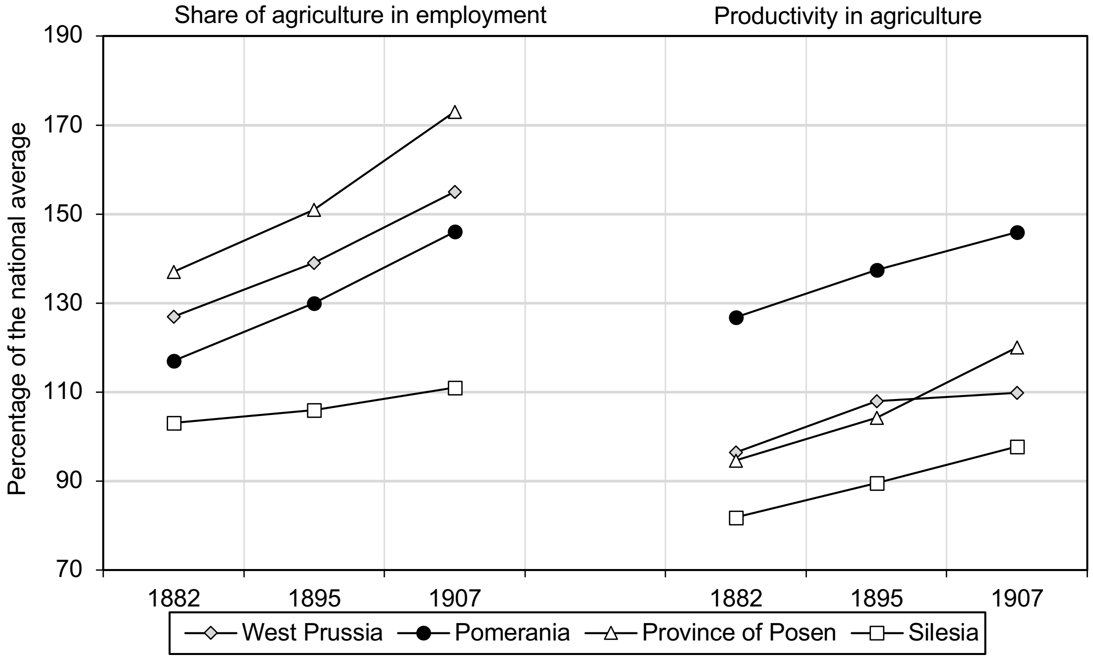

Figure OA5: Shares of agriculture in employment and productivity in agriculture, as a percentage of the national average
Source: the data from Tipton 1976, Table 6.2 (p.106); Grant 2002, Tab. 2 (net value added per full-time labour unit).

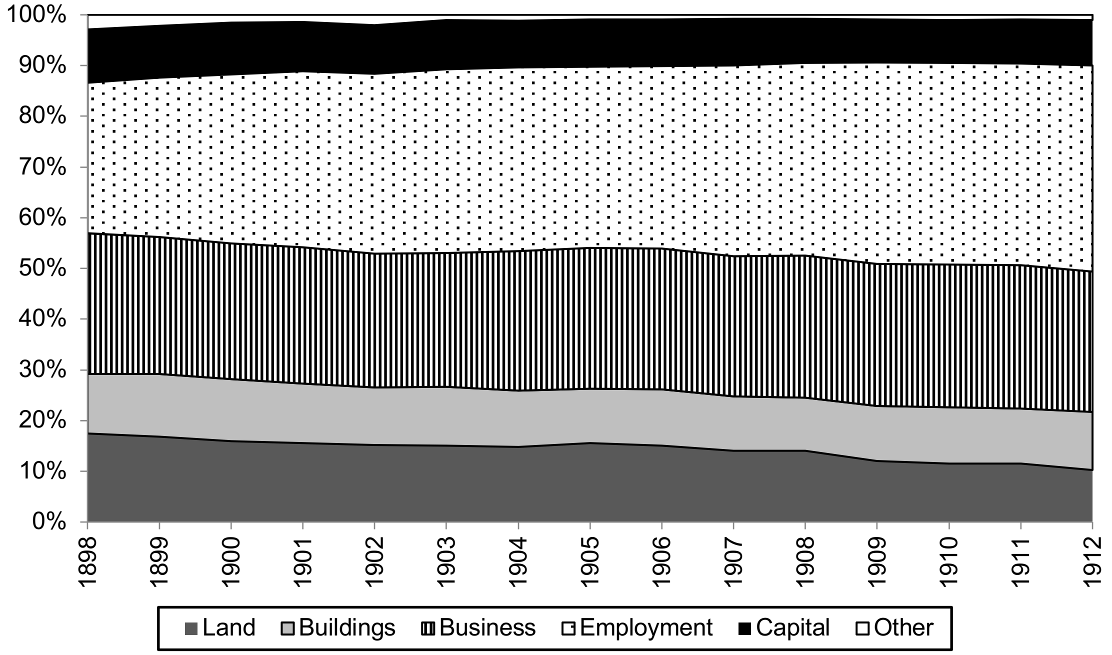

Figure OA6: Galicia: total taxed income by sources.
Source: authors' computation based on income tax statistics.

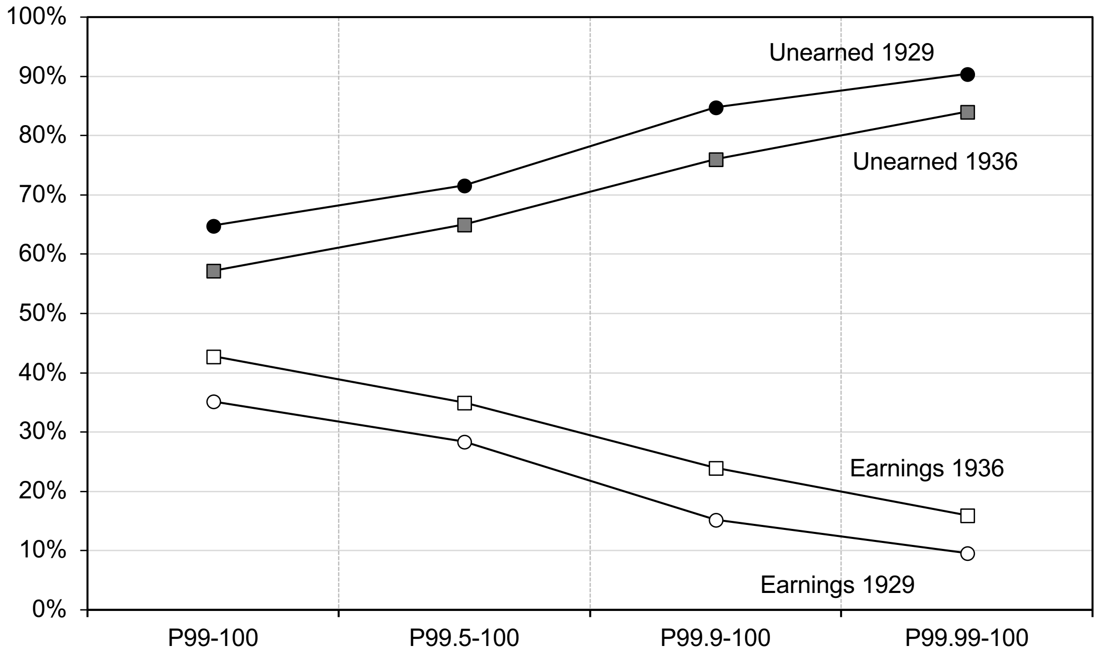

Figure OA7: The composition of top groups by income source
Source: author's computation based on income tax statistics.

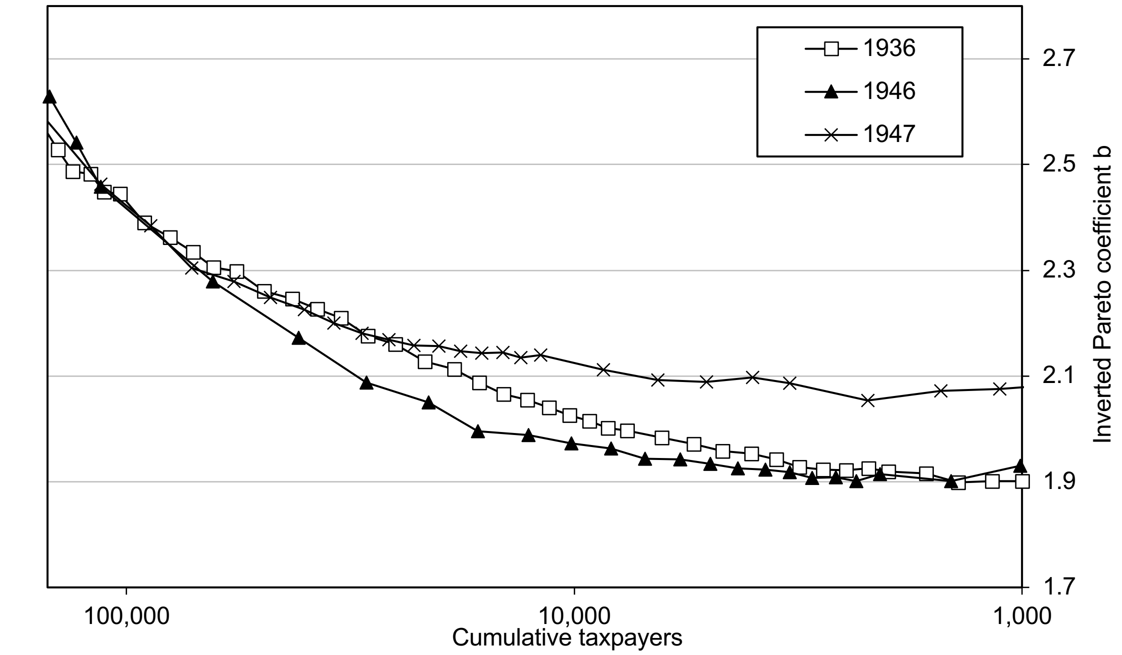

Figure OA8: Inverted Pareto coefficient b for 'unearned' income
Source: authors' computation from income tax statistics
Note: dots on lines present the number of cumulated taxpayers above the specific bracket threshold.

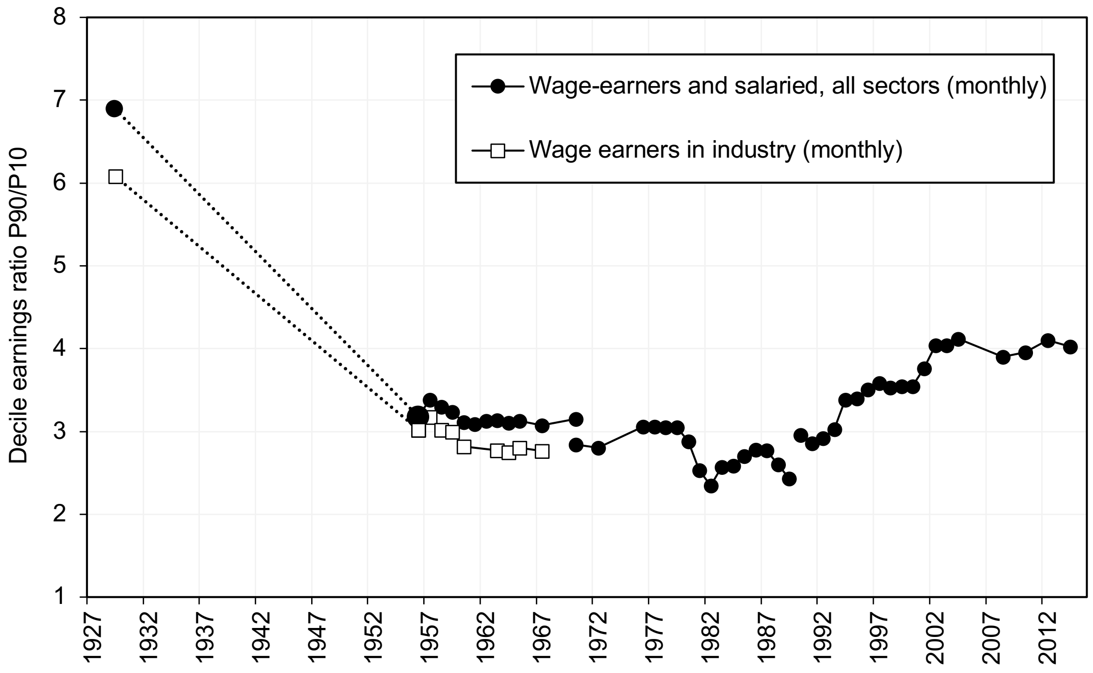

Figure OA9: Long-run evolution of the earnings decile ratio P90/P10
Source: see sources for Figure 8

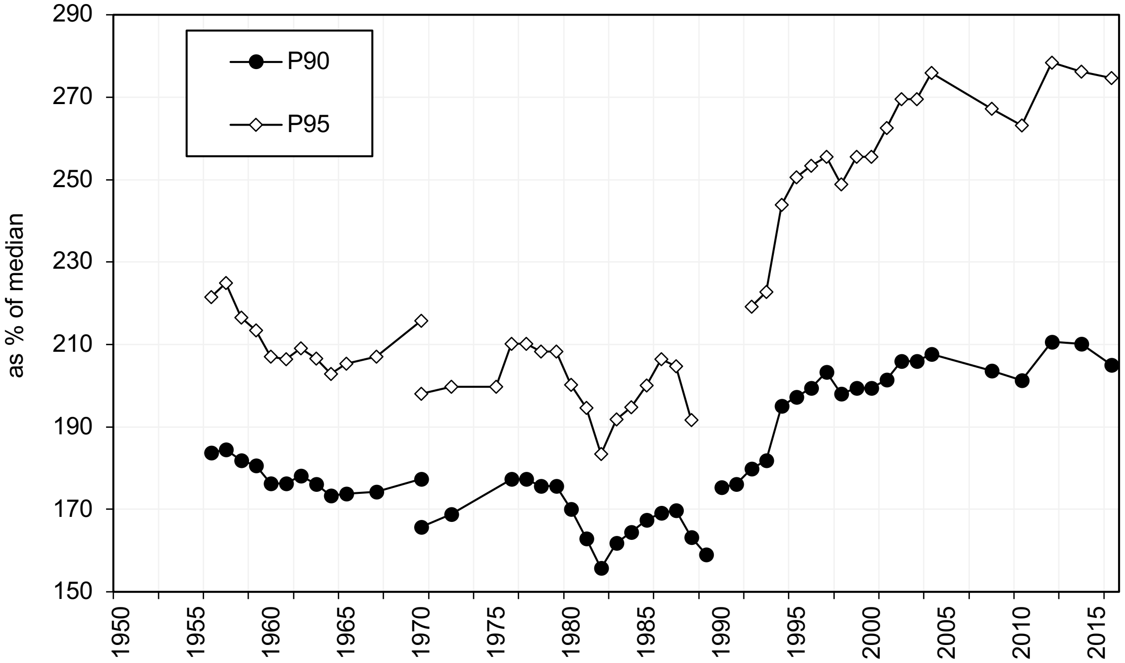

Figure OA10: Development of the upper tail earnings distribution: P95/P50 and P90/P50
Source: see sources for Figure 8

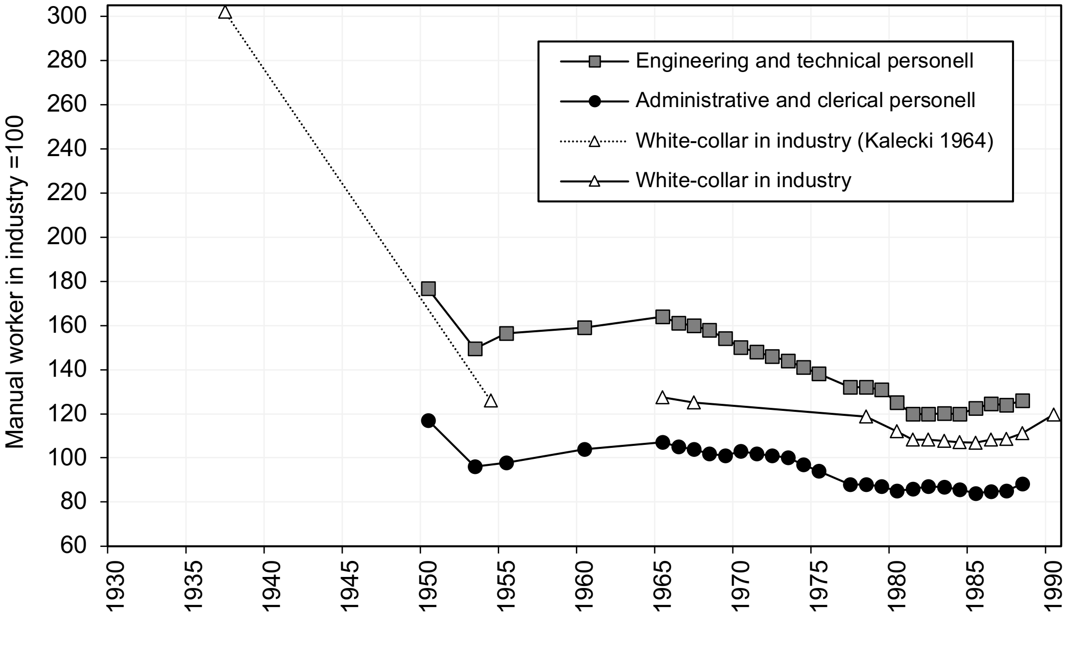

Figure OA11: Occupational earning gaps in industry
Source: Earnings gap between white-collar and manual in 1938 and 1954 from Kalecki (1964); other years from Statistical Yearbook of Poland. Earnings premium for engineering and technical personnel and administrative and clerical personnel until 1980 from Adam 1984, T. 11.6, for the 1980s from Statistical Yearbook.
Note: break in the data in 1975 due to new classification (Adam 1984, p. 203)

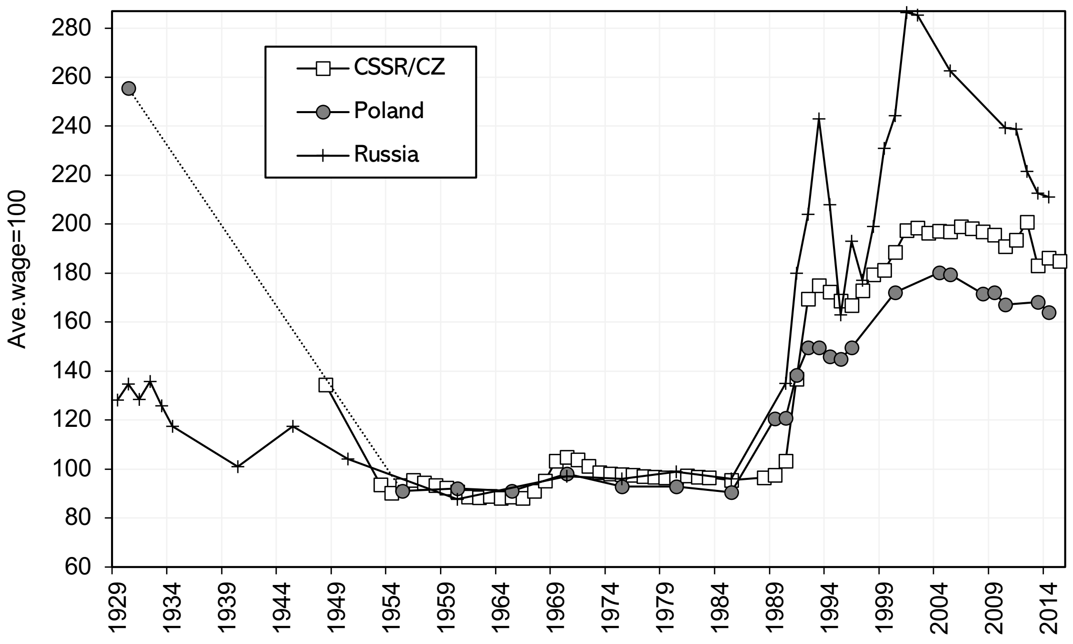

Figure OA12: Development of relative wage in finance

Source: *Poland*. 1929: own estimation from the insurance data of employees in banking and insurance (*St.Pracy* 1931, 4; we combine tabulations for managerial and clerical employees (*personel kierowniczy* and *pracownicy biurowi*)). Average wage in private, non- farm sector estimated from Wisniewski 1934, T.15). Other years from Statistical Yearbook and Statistical Yearbook of Labour Statistics. *Czechoslovakia/Czechia*. 1948 from Adam 1984, p. 193; other years from *Historická statistická ročenka ČSSR* 1985, p. 153, Statistical Yearbook. USSR/Russia. *Trud v SSSR. Statisticheskii sbornik* (Moskva: Finansy i statistika, 1988), Statistical yearbook (section *Trud*).

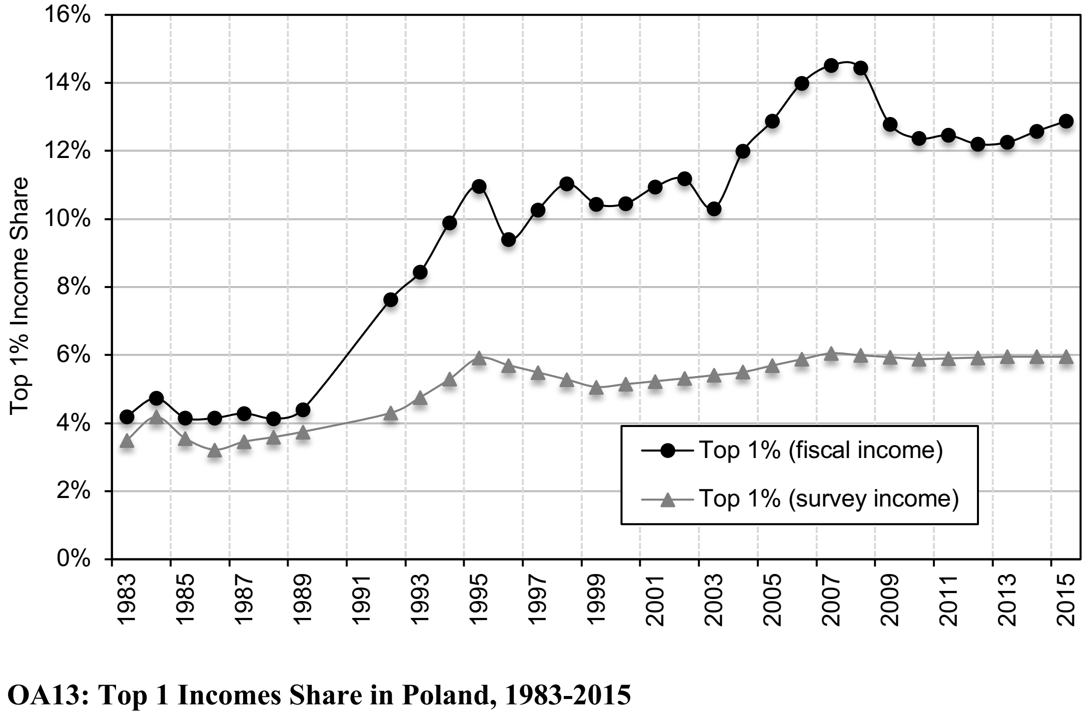

Figure OA13: Top 1 Incomes Share in Poland, 1983-2015

Source: Authors' computation (see Section 2). Distribution of pre-tax national income (before taxes and transfers, except pensions and unemployment. insurance) among equal-split adults

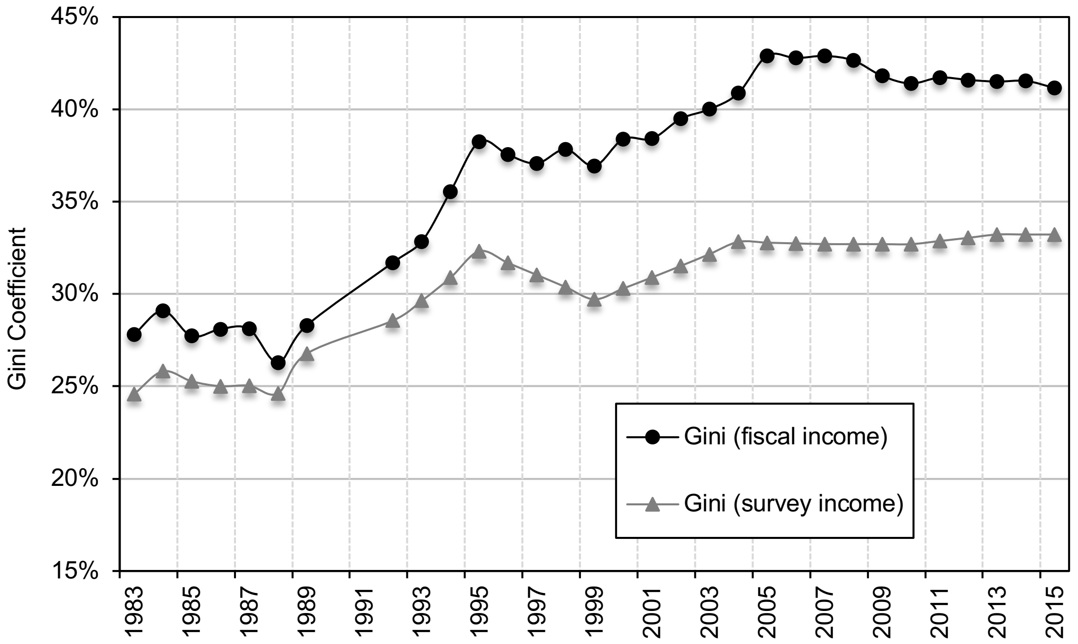

Figure OA14: Gini coefficient in Poland, 1983-2015
Source: Authors' computation (see Section 2). Distribution of pre-tax national income (before taxes and transfers, except pensions and unemployment insurance) among equal-split adults

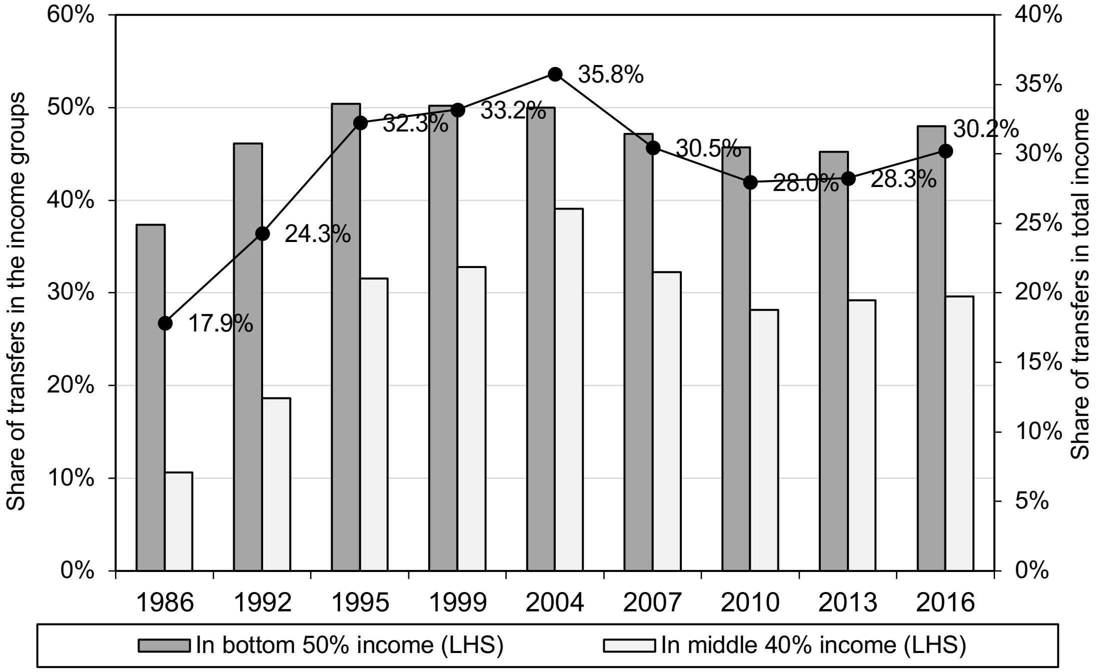

Figure OA15: Share of transfers in the bottom 50 and middle 40 income groups.
Source: Authors' computation (see Section 2). Distribution of income reported in HBS among equal-split adults

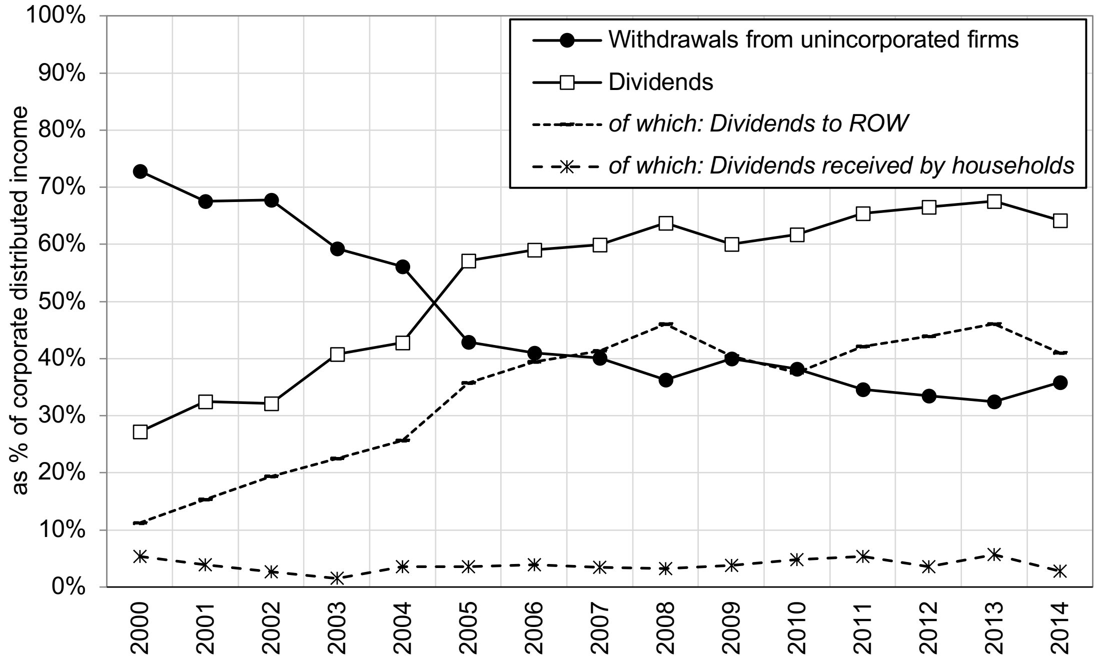

Figure OA16: Distributed income from corporations, 2000-2014
Source: Central Statistical Office of Poland, National Accounts. `Withdrawals' from unincorporated firms is received in total by households

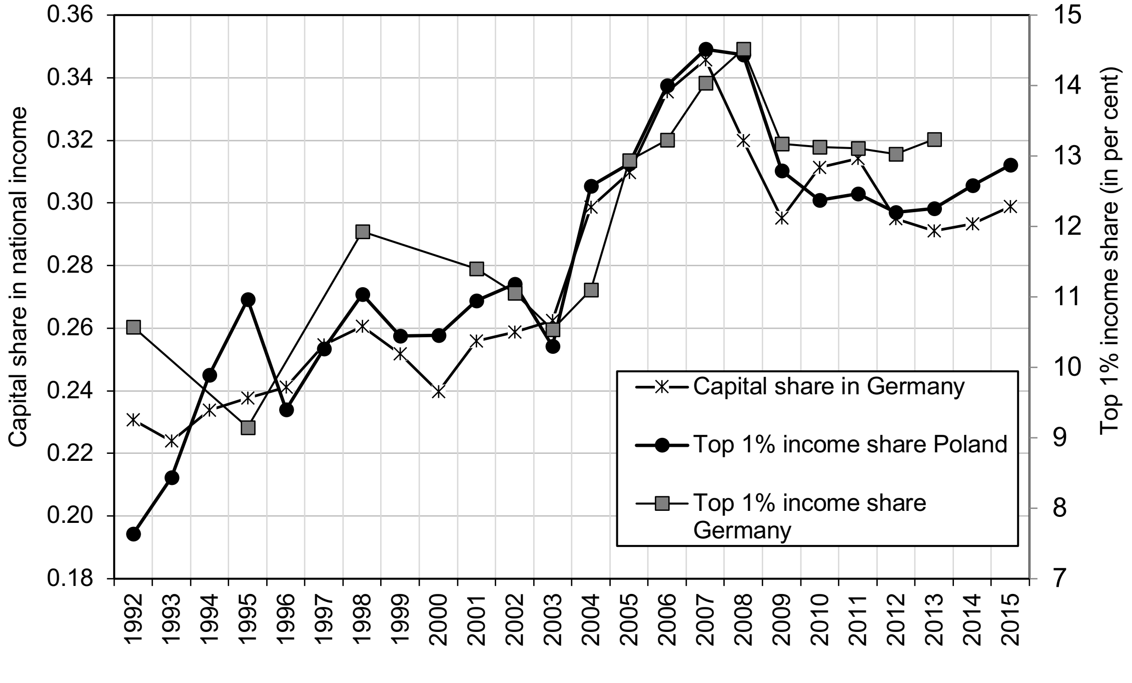

Figure OA17: Capital share in national income in Germany; top 1 income share in Germany and Poland (distribution of fiscal income among tax units).
Source: WID

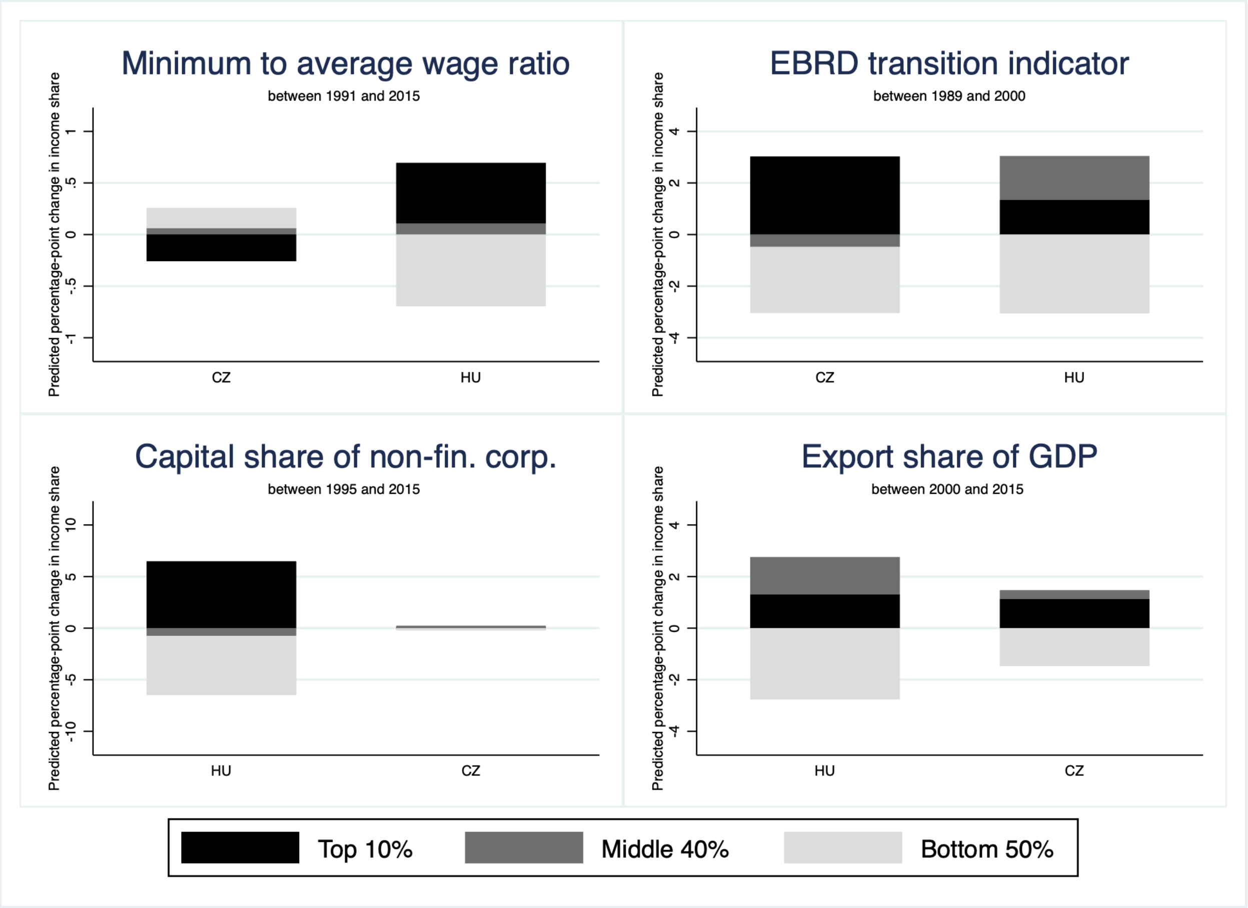

Figure OA18: The predicted (non-causal) effect of the cumulative change in minimum wage, institutional transition, capital share and export share on income shares in Czech Republic and Hungary.

Source: Minimum wage to average wage ratio: OECD and author's computation; EBRD transition indicator: EBRD; Capital share and export share: OECD. Income share: authors' computation and WID. Distribution of fiscal income among tax units Note: the upper left (right) panel shows the estimated non-causal change in income shares due to the actual change in minimum wage to average wage ratio (EBRD transition indicator) between 1991 (1989) and 2015 (2000). the bottom left (right) panel shows the estimated non-causal change in income shares due to the actual change in capital share (export share) between 1995 (2000) and 2015. The estimates are constructed from country-specific correlation coefficients between capital share (export share) and each income share, estimated from a panel fixed effect country-level regression (for Poland, Germany, Hungary and Czech Republic), which includes a full set of year fixed effects. The bars are calculated by multiplying the estimated coefficients for each income share and country by the actual change in the independent variable.

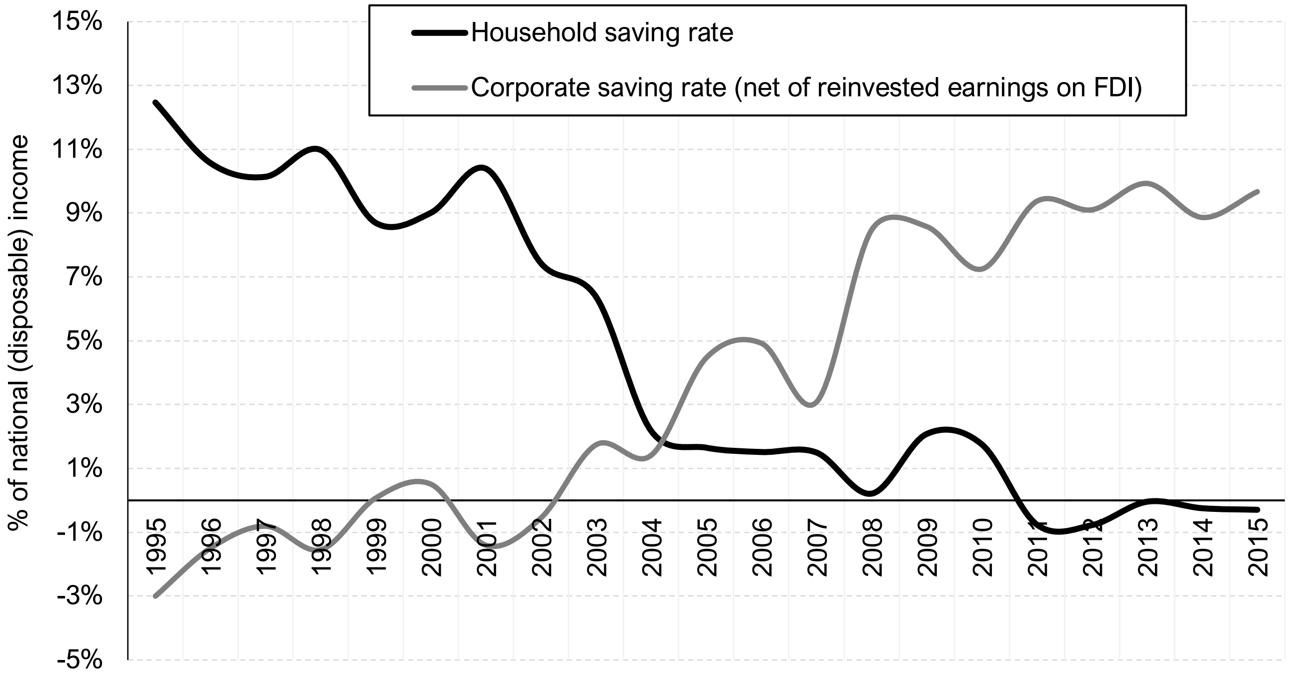

Figure OA19: Sectoral composition of the private saving rate in Poland, 1995-2015
Source: Eurostat. Note: Private saving rate in Poland is composed of saving of the household sector (S.14) and of the non-financial corporate sector (S.13).

Literature

Adam, J. (1984). *Employment/Wage Policies in Poland, Czechoslovakia and Hungary Since 1950*. Springer.

Alvaredo, F., Atkinson, A., Chancel, L., Piketty, T., Saez, E. and Zucman, G. (2016). "Distributional National Accounts (DINA) Guidelines: Concepts and Methods used in WID.world", WID.world Working Paper 2016/02.

Atkinson, A. B (2007). Measuring Top Incomes: Methodological Issues. In Atkinson, A. B., and Piketty, T. (2007). Top Incomes over the Twentieth Century: A Contrast between Continental European and English-Speaking Countries (Eds.), Oxford: Oxford University Press.

Atkinson, A. B. (2008). *The changing distribution of earnings in OECD countries*. Oxford: Oxford University Press.

Atkinson, A. B., and Micklewright, J. (1992). *Economic Transformation in Eastern Europe and the Distribution of Income*. Cambridge: Cambridge University Press.

Atkinson, A. B., and Piketty, T. (2007). *Top Incomes over the Twentieth Century: A Contrast between Continental European and English-Speaking Countries* (Eds.), Oxford: Oxford University Press.

Atkinson, A. B., and Piketty, T. (2010). *Top Incomes: A Global Perspective* (Eds.), Oxford: Oxford University Press.

Bergson, A. (1944). *The Structure of Soviet Wages–a Study in Socialist Economics*. Cambridge: Harvard University Press

Bergson, A. (1984). “Income inequality under Soviet socialism”. *Journal of Economic Literature*, 22, 1052–1099

Blanchet, T., Fournier, J., and Piketty, T. (2017). “Generalized Pareto Curves: Theory and Applications”, WID.world Working Paper 2017/03.

Bukowski, P., Chrostek, P., Novokmet, F. and Skawinski, M. (2021). “The Distributional National Accounts in Poland: 2004-2017”, forthcoming manuscript.

Ciccarelli, C., and Missiaia, A. (2014). “Business fluctuations in Imperial Austria's regions, 1867-1913: new evidence”. LSE Economic History Working Papers No. 186/2014

Cvrcek, T. (2013). Wages, prices, and living standards in the Habsburg empire, 1827–1910. *The Journal of Economic History*, 73(1), 1-37.

Czajkowski, T. (1934), “Proba szacunkowego obliczenia zarobkow robotniczych w Polsce”, *Statystyka Pracy*, (4),

225-236.
Davies, N. (2005), God's Playground A History of Poland: Volume II: 1795 to the Present, Vol. 2, Oxford University Press.
Dell, F. (2007). "Top Income in Germany throughout the Twentieth Century: 1891–1998". In A. B. Atkinson and T. Piketty (eds.), *Top Incomes over the Twentieth Century: A Contrast between Continental European and English-Speaking Countries*, (pp. 365-425), Oxford: Oxford University Press.
Dell, F. (2008). *L'Allemagne inégale: inégalités de revenus et de patrimoine en Allemagne, dynamique d'accumulation du capital et taxation de Bismarck à Schröder 1870-2005*, Doctoral dissertation, Paris, EHESS.
Derengowski, J. (1930). „Płace robotników przemysłowych w Polsce w latach 1924-1929." *Statystyka Pracy*, (1), 118-122.
Dumke, R. (1991). "Income Inequality and Industrialisation: the Kuznets curve reexamined" in Y. Brenner, H. Kaelbe, and M. Thomas (eds.), *Income Distribution in Historical Perspective*, Cambridge: Cambridge University Press.
Dziemianowicz, R. I. (2007). Efektywność systemu opodatkowania rolnictwa. Wydawnictwo Uniwersytetu w Białymstoku.
Finanz-ministerium (various years). *Mitteilungen des K.K. Finanz-Ministeriums*. Wien: Hof und Staatsdruckerei.
Flakierski, H. (1986). Economic Reform & Income Distribution: A Case Study of Hungary and Poland. Armonk, New York/London: M.E Sharpe.
Flakierski, H. (1991). "Social policies in the 1980s in Poland: A discussion of new approaches". In *Economic Reforms and Welfare Systems in the USSR, Poland and Hungary* (pp. 85-109). London: Palgrave Macmillan.
Gerlicz R. (1929). *Praca Najemna na Roli w Większej Własności Ziemskiej*, Warszawa: Wydawnictwo Rady Naczelnej Organizacji Ziemiańskich.
Główny Urząd Statystyczny (1919-1939). *Statystyka Pracy*. Główny Urząd Statystyczny, Warszawa
Główny Urząd Statystyczny. (1949). *Dochód Narodowy Polski 1947*, Glówny Urzad Statystyczny. Statystyka Polski ; seria D, Warszawa.
Główny Urząd Statystyczny. (2017). *Rocznik Demograficzny*. Główny Urząd Statystyczny, Warszawa.
Grant, O. (2002). "Productivity in German agriculture: estimates of agricultural productivity from regional accounts for 21 German regions: 1880/4, 1893/7 and 1905/9", *University of Oxford Discussion Papers in Economic and Social History, 47.*
Gregory, P. (1982). *Russian national Income, 1885-1913*. Cambridge University Press.
Hoffmann, W. (1965). *Das Wachstum der deutschen Wirtschaft seit der Mitte des 19. Jahrhunderts*. Enzyklopädie der Rechts- und Staatswissenschaft. Springer. Berlin Heidelberg New York.
Hoffmann W. G, and Müller, J. H. (1959). *Das deutsche Volkseinkommen 1851-1957*. Tübingen: Mohr.
Jędruszczak, H. (1964). *Płace robotników przemysłowych w Polsce w latach 1924-1939*. Warszawa.
Jezierski, A.(1967). *Handel zagraniczny Krolestwa Polskiego 1815-1914*, Warsaw 1967
Jezierski, A. (1984). *Problemy Rozwoju gospodarczego ziem Polskich w XIX I XX wieku*. Warsaw
Jezierski, A. and Leszczynksa, C. (2003). *Historia Gospodarcza Polski*, Wydawnictwo Key Text, Warsaw
Kaeble, H. (1986). *Industrialization and social inequality in 19th Century Europe*. Leamington Spa: Berg
k. k. Statistische Zentralkommission (various years). *Österreichisches statistisches Handbuch für die im Reichsrate vertretenen Königreiche und Länder : nebst einem Anhange für die gemeinsamen Angelegenheiten der österreichisch-ungarischen Monarchie*
k. k. Statistische Zentralkommission (1890, 1900, 1910). *Die Ergebnisse der Volkszählung den im Reichsrathe vertretenen Königreichen und Ländern*. Wien: Gerold & Sohn
Kalecki M., and Landau, L. (1934). *Szacunek dochodu społecznego w r. 1929*, Instytut Badania Koniunktur Gospodarczych i Cen, Badania nad dochodem społecznym w Polsce, t. 1. Warszawa.
Kalecki M., and Landau, L. (1935). "Social income in 1933 and the foundations of periodic studies on changes of income". In J. Osiatyński (Ed.), *Collected works of Michał Kalecki, Vol. VI, Studies in Applied Economics,* Oxford: Clarendon press, 1996, 436-483.
Klarner, C. (1937), *Dochód społeczny wsi i miast w Polsce w okresie prze silenia gospodarczego 1929—1936*, Lwów.
Kopczuk, W. (2012). "The Polish business "flat" tax and its effect on reported incomes: a Pareto improving tax reform?", *mimeo.*
Königliches Statistisches Landesamt (various years). *Statistik der preussischen Einkommensteuer-Veranlagung*, Berlin.
Königliches Statistisches Bureau (various years). *Statistisches Handbuch für den Preussischen Staat.* Berlin: Königlichen Statistischen Bureau.

Königliches Statistisches Bureau (various years). *Statistisches Jahrbuch für den Preussischen Staat*. Verlag des Königlichen Statistischen Landesamts.

Kuznets, S. (1955). "Economic growth and income inequality". *American Economic Review* 45, 1-28.

Landau, L. (1933). *Płace w Polsce w związku z rozwojem gospodarczym*. Instytut Spraw Spolecznych, Warszawa.

Landau, Z. (1976). "National income in historical research (On material from the period of interwar Poland)", *Acta Poloniae Historica* 33

Łaski, K. (1956). *Akumulacja i spożycie w procesie uprzemysłowienia Polski Ludowej.* Książka i Wiedza.

Luxembourg Income Study (LIS) Database, http://www.lisdatacenter.org (Poland; 1992-2013; access 9/11/2017) Luxembourg: LIS.

Maddison, A. (2001). "The World Economy: A Millenial Perspective, Development Centre of the Organization for Economic Cooperation and Development". OECD, Paris, 3, 162-193.

Mieszczankowski, M. (1960). *Struktura agrarna Polski międzywojennej*. Warszawa: Państwowe Wydawnictwo Naukowe.

Milanović, B. (1998). *Income, inequality, and poverty during the transition from planned to market economy*. Washington, DC: World Bank.

Morrisson, C. (1984). "Income Distribution in East European and Western Countries," *Journal of Comparative Economics*, 8(2), 121–138.

Müller, J. H., and Geisenberger, S. (1972). *Die Einkommensstruktur in verschiedenen deutschen Ländern: 1874-1913 unter Berücksichtigung regionaler Verschiedenheiten* (Vol. 10). Duncker & Humblot.

Nafziger, S., and Lindert, P. H. (2012). *Russian inequality on the eve of revolution*. National Bureau of Economic Research No. w18383

Novokmet, F. (2017). *Between Communism and Capitalism: Essays on the Evolution of Income and Wealth Inequality in Eastern Europe 1890–2015 (Czech Republic, Poland, Bulgaria, Croatia, Slovenia, and Russia)*. PhDdiss. Paris School of Economics

Novokmet, F., Piketty, T., and Zucman, G. (2018). "From Soviets to oligarchs: Inequality and property in Russia 1905-2016." *The Journal of Economic Inequality*, 16(2), 189-223.

Pareto, V. (1896). *Course of political economy*. Lausanne.

Pawłowska-Tyszko, J., Soliwoda, M., Pieńkowska-Kamieniecka, S., and Walczak, D. (2015). *Stan obecny i perspektywy rozwoju systemu podatkowego i ubezpieczeniowego polskiego rolnictwa*. Instytut Ekonomiki Rolnictwa i Gospodarki Żywnościowej-Państwowy Instytut Badawczy.

Petyniak-Sanecki, K. (1939). *Współczesne zagadnienia gospodarcze*, Part II, Lwów.

Philippon, T., and Reshef, A. (2012). "Wages and human capital in the US finance industry: 1909–2006." *The Quarterly Journal of Economics*, 127(4), 1551-1609.

Piketty, T., Yang L. and Zucman, G. (2019). "Capital Accumulation, Private Property and Rising Inequality in China, 1978-2015", American Economic Review, Forthcoming.

Prokopovitch, S. (1926), "The Distribution of National Income", *Economic Journal*, 36, 69–82.

Rutkowski, J. (1996). "High skills pay-off: The changing wage structure during economic transition in Poland", *Economics of Transition*, 4, 89–112.

Rutkowski, J. (2001). "Earnings inequality in transition economies of Central Europe: Trends and patterns during the 1990s." World Bank Social Protection Discussion Paper No. 0117.

Schulze, M. S. (2007). "Regional income dispersion and market potential in the late nineteenth century Hapsburg Empire". LSE Economic History Working Papers No. 106/07

Statistisches Reichsamt (various years). *Statistisches Jahrbuch fr das deutsche Reich*. Statistisches Reichsamt.

Statistisches Reichsamt (1932). „Das deutsche Volkseinkommen vor und nach dem Kriege". *Einzelschriften zur Statistik des deutschen Reichs*, vol. 24. Berlin: Reimar Hobbing.

Szturm De Sztrem, T. (1922). *Walka o płace zarobkowe*. Instytut Gospodarstwa Społecznego, Warszawa

Tipton, F. B. (1976). *Regional variations in the economic development of Germany during the nineteenth century*. Wesleyan University Press.

Wiśniewski, J. (1934). *Rozkład dochodów według wysokości w r. 1929*, Instytut Badań Koniunktur Gospodarczych i Cen, Badania nad dochodem społecznym w Polsce. t. III. Warszawa.

Wolf, N. (2007). "Endowments vs. market potential: What explains the relocation of industry after the Polish reunification in 1918?", Explorations in Economic History, 44(1), 22-42.

Załęski, W. (1901). *Królestwo Polskie pod względem statystycznym: Statystyka zajęć i przemysłu*. Skł. gł. w Księgarni Jana Fiszera.
